# ATRX affects the repair of telomeric DSBs by promoting cohesion and a DAXX-dependent activity

**DOI:** 10.1371/journal.pbio.3000594

**Published:** 2020-01-02

**Authors:** Courtney A. Lovejoy, Kaori Takai, Michael S. Huh, David J. Picketts, Titia de Lange

**Affiliations:** 1 Laboratory for Cell Biology and Genetics, The Rockefeller University, New York, New York, United States of America; 2 Regenerative Medicine Program, Ottawa Hospital Research Institute, Ottawa, Ontario, Canada; 3 Department of Biochemistry, Microbiology and Immunology, University of Ottawa, Ottawa, Ontario, Canada; 4 Department of Cellular and Molecular Medicine, University of Ottawa, Ottawa, Ontario, Canada; The University of Texas, at Austin, UNITED STATES

## Abstract

Alpha thalassemia/mental retardation syndrome X-linked chromatin remodeler (ATRX), a DAXX (death domain-associated protein) interacting protein, is often lost in cells using the alternative lengthening of telomeres (ALT) pathway, but it is not known how ATRX loss leads to ALT. We report that ATRX deletion from mouse cells altered the repair of telomeric double-strand breaks (DSBs) and induced ALT-like phenotypes, including ALT-associated promyelocytic leukemia (PML) bodies (APBs), telomere sister chromatid exchanges (T-SCEs), and extrachromosomal telomeric signals (ECTSs). Mechanistically, we show that ATRX affects telomeric DSB repair by promoting cohesion of sister telomeres and that loss of ATRX in ALT cells results in diminished telomere cohesion. In addition, we document a role for DAXX in the repair of telomeric DSBs. Removal of telomeric cohesion in combination with DAXX deficiency recapitulates all telomeric DSB repair phenotypes associated with ATRX loss. The data reveal that ATRX has an effect on telomeric DSB repair and that this role involves both telomere cohesion and a DAXX-dependent pathway.

## Abstract 2

The chromatin remodeller ATRX is often lost in cells that maintain their telomeres using the telomerase-independent “alternative lengthening of telomeres” (ALT) pathway. This study shows how loss of ATRX alters the repair of telomeric DNA breaks and suggests how this change could promote telomere maintenance in ALT cells.

## Introduction

A subset of human cancers and immortalized cell lines maintains telomeres in the absence of telomerase activity using the alternative lengthening of telomeres (ALT) pathway [1–3]. ALT cells are characterized by frequent telomere sequence exchanges, clustering of telomeres in promyelocytic leukemia (PML) bodies (forming ALT-associated PML bodies [APBs]), long and heterogeneous telomere lengths, and extrachromosomal telomeric repeat DNA detectable as extrachromosomal telomeric signals (ECTSs) and circular telomeric G- or C-strand DNA [1,4–11]. The major genetic alteration associated with ALT is loss of alpha thalassemia/mental retardation syndrome X-linked chromatin remodeler (ATRX), and reintroduction of ATRX results in repression of ALT [12–21]. ATRX is a member of the sucrose nonfermenting (SNF2) family of chromatin remodeling proteins, with diverse functions and numerous interaction partners [22–29]. ATRX associates with the histone chaperone death domain-associated protein (DAXX) to deposit the histone variant H3.3 at telomeres and pericentric heterochromatin in a replication-independent chromatin assembly pathway [30–32]. Mutations in DAXX and H3.3 have been reported in tumors exhibiting hallmarks of ALT, but are considerably less frequent than ATRX mutations and have not been found in ALT cell lines established in vitro [12,14–18,20]. How ATRX loss promotes ALT remains unclear, although various mechanisms have been proposed [19,33].

ALT cells maintain telomeres through homology-directed repair (HDR) [34,35]. Recently, it was shown that ALT cells can synthesize telomeric DNA using an HDR pathway that resembles break-induced replication (BIR) [36–39]. This pathway is thought to extend a telomeric double-strand break (DSB) using the same telomere, the sister telomere, extrachromosomal telomeric DNA, or the telomere of another chromosome as a template. Whether this BIR-like pathway is the only mechanism of telomere extension in ALT or operates in combination with other forms of HDR is not yet clear. How ATRX loss promotes BIR or other types of HDR at telomeres has not been established, nor is it clear how the telomeric DSBs arise that initiate recombination.

Here, we show that ATRX specifically promotes telomere cohesion and study how this change in cohesion affects DNA repair. The canonical cohesin complex, containing structural maintenance of chromosomes 1 (SMC1), structural maintenance of chromosomes 3 (SMC3), Rad21 cohesin complex component (RAD21), and either stromal antigen 1 or 2 (SA1 or SA2), functions in both the accurate segregation of sister chromatids during mitosis and the repair of DSBs in G2 [40]. Efficient sister chromatid–based DSB repair requires both cohesion established during DNA replication and the generation of post-replicative, damage-induced cohesion [41–43]. The DNA damage response promotes cohesion proximally to a DSB by facilitating the recruitment of additional cohesin molecules to the chromatin surrounding the break site [42,44–48]. The physical pairing of sister chromatids by cohesin represses unequal sister chromatid recombination, blocks interchromosomal HDR, and prevents the joining of distal ends [49–55]. By analogy, it is expected that loss of telomere cohesion, and the lack of sister chromatid alignment that results, may be relevant to ALT. In-register HDR or BIR between allelic telomeres would not result in net telomere elongation and is inconsistent with the observation that the copying of a molecular tag from one telomere to another, nonallelic telomere is frequent in ALT [34]. Telomere cohesion is uniquely dependent on the cohesin subunit SA1 [56,57], but no mutations in *SA1* have been found in ALT cell lines [17].

We report that loss of ATRX caused a telomere-specific cohesion defect that enables interactions between nonallelic telomeres. ATRX deletion altered the repair of FokI nuclease domain and telomeric repeat binding factor 1 fusion protein (FokI-TRF1)–induced telomeric DSBs, enhancing telomere recombination, formation of extrachromosomal telomeric DNA (a predicted by-product of BIR), and APBs. However, the effects of ATRX loss are not fully recapitulated by disrupting telomere cohesion through deletion of SA1. We show that ATRX loss can be phenocopied by removal of both telomere cohesion and deletion of DAXX, indicating that the role of ATRX in telomeric DSB repair choice involves both cohesion and DAXX-dependent activities.

## Results

### ATRX deletion alters the repair of telomeric DSBs

To examine the role of ATRX in the repair of telomeric DSBs, we turned to ATRX conditional knockout (KO) mouse embryonic fibroblasts (MEFs) as a means of isolating ATRX deficiency without the confounding effects that may be associated with tumor cell lines or cell lines that have escaped from telomere crisis. MEFs have telomeres that are similar in length to the longest human telomeres, including those in ALT cells. Although MEFs express low levels of telomerase, there are no data indicating that telomerase affects the mechanism of DSB repair in telomeric DNA or the function of ATRX. In human ATRX-deficient cells, human telomerase reverse transcriptase (hTERT) expression does not abolish ALT [58–60] and telomerase does not prevent human or mouse telomeres from undergoing recombination [61–63]. Since *ATRX* is located on the X chromosome, the MEFs used in these studies were derived from either a female embryo with two floxed ATRX alleles (ATRX^F/F^) or a male embryo with a single floxed allele (ATRX^F/y^). MEFs were immortalized with simian virus 40 (SV40) large T antigen, similar to the procedure used for many human ALT cells lines [17,21].

**Fig 1:**
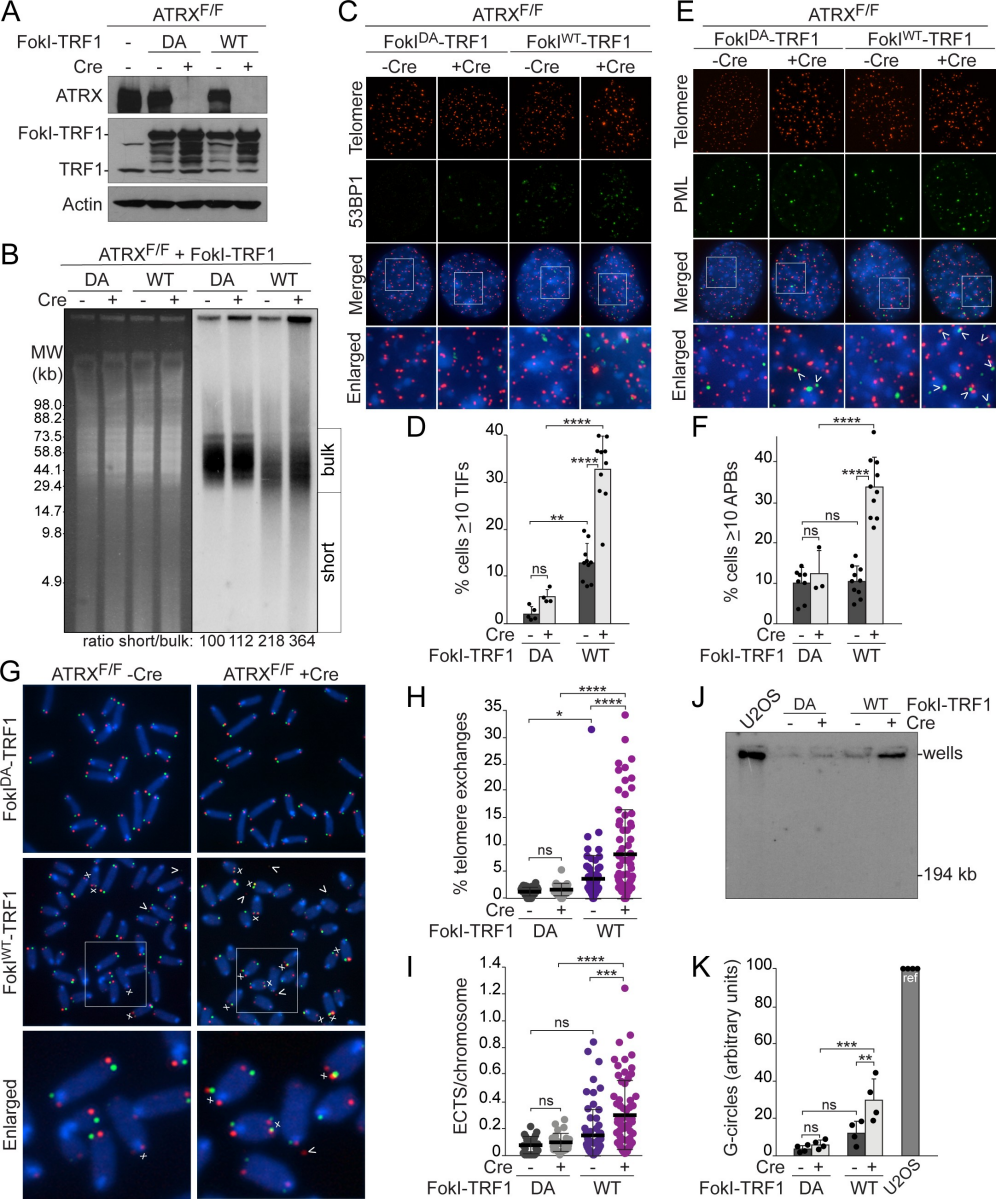
Altered repair of telomeric DSBs after ATRX deletion. **(A)** Immunoblots for FokI-TRF1 (6 days) in ATRX^F/F^ MEFs treated with Cre (4 days). Actin serves as a loading control. **(B)** PFGE of telomeric DNA. Left, ethidium bromide–stained gel and right, detection of telomeric restriction fragments by native in-gel hybridization with a [CCCTAA]_4_ probe for the telomeric overhang. The ratio of short versus bulk telomere signal (mean from 3 experiments) is given below the blot, with values reported relative to FokI^DA^-TRF1 (set at 100). **(C)** TIFs detected by IF-FISH for 53BP1 (IF, green) and telomeres (FISH, red). DNA was stained with DAPI. **(D)** Quantification of the TIF response as in (C). Bars: means and SDs from at least 4 experiments of >100 cells each. **(E)** APBs (arrows) detected by IF-FISH for PML (IF, green) and telomeres (FISH, red). DNA was stained with DAPI. **(F)** Quantification of the percentage of cells with ≥10 APBs, as assayed in (E). Bars: means and SDs of at least 3 experiments of ≥100 cells each. **(G)** CO-FISH on metaphase spreads. Telomeres replicated by leading strand DNA synthesis were labeled by PNA-FISH with an Alexa-647-[TTAGGG]_3_ probe (red) and lagging strand telomeres with a Cy3-[CCCTAA]_3_ probe (green). Chromosome ends with telomere exchanges are indicated by an “x” and ECTSs are marked by arrows. **(H)** Quantification of telomere exchanges detected by CO-FISH. Each data point represents the percentage of chromosome ends with telomere exchanges in one metaphase spread. Bars: means and SDs of >60 metaphases from 8 experiments (23 metaphases from 3 experiments for FokI^DA^-TRF1+Cre). **(I)** Quantification of ECTSs from metaphase spreads as in (G, H). Each data point represents the number of ECTSs per chromosome in one metaphase spread. **(J)** Representative G-circle assay using 30 ng gDNA from ALT^+^ U2OS and ATRX^F/F^ MEFs expressing FokI-TRF1. The amplified products were detected by in-gel hybridization with an end-labeled ^32^P-[TTAGGG]_4_ probe. **(K)** Quantification of G-circle amplification signals from the indicated MEFs, reported relative to ALT^+^ U2OS (set at 100). Bars: means and SDs from 4 experiments. All *p*-values were derived from a one-way ANOVA with Tukey correction. ****p < 0.0001, ***p < 0.001, **p < 0.01, *p < 0.05. The underlying numerical data and statistical analysis for each figure panel can be found in S1 Data. ALT^+^, alternative lengthening of telomeres-positive; APB, ALT-associated PML body; ATRX, alpha thalassemia/mental retardation syndrome X-linked chromatin remodeler; ATRX^F/F^, female embryo with two floxed ATRX alleles; CO-FISH, chromosome orientation fluorescence in situ hybridization; Cre, recombinase acting on Lox sites; DA, nuclease dead FokI-TRF1; DSB, double-strand break; ECTS, extrachromosomal telomeric signal; FISH, fluorescence in situ hybridization; FokI-TRF1, FokI nuclease domain and telomeric repeat binding factor 1 fusion protein; gDNA, genomic DNA; IF, immunofluorescence; MEF, mouse embryonic fibroblast; PFGE, pulsed-field gel electrophoresis; PML, promyelocytic leukemia; PNA-FISH, peptide nucleic acid fluorescence in situ hybridization; SD, standard deviation; TIF, telomere dysfunction–induced foci; U2OS, human osteosarcoma cell line; WT, wild-type FokI-TRF1; 53BP1, tumor protein p53-binding protein 1.

To induce telomeric DSBs, we used the previously characterized FokI^WT^-TRF1 fusion protein [64–66] or a nuclease-dead control (FokI^DA^-TRF1). Localization of FokI-TRF1 to telomeres does not affect the function of shelterin [66]. The fusion proteins were introduced by retroviral infection prior to Cre recombinase-mediated deletion of ATRX and were overexpressed 4- to 5-fold relative to endogenous TRF1 (Fig 1A). As expected, FokI^WT^-TRF1, but not FokI^DA^-TRF1, induced phenotypes indicative of telomeric DSBs, including shorter telomere restriction fragments and telomere dysfunction–induced foci (TIFs) [67], as evidenced by the co-localization of tumor protein p53 binding protein 1 (53BP1) with fluorescence in situ hybridization (FISH)-labeled telomeres (Fig 1B–1D). A small increase in FokI-independent telomere damage was observed in ATRX-deficient cells, a previously reported effect of unknown significance [68–71]. Interestingly, deletion of ATRX resulted in an increased abundance of shortened telomeres and TIFs after expression of FokI^WT^-TRF1 (Fig 1A–1D). Because ATRX deficiency did not alter the S-phase index of immortalized MEFs (S1A Fig), this phenotype is not due to a change in cell cycle progression.

The ATRX-deficient cells with telomeric DSBs showed an increase in the co-localization of telomeres with PML bodies, forming APBs. In contrast, control cells contained few APBs and variable numbers of PML foci, and neither deletion of ATRX nor expression of FokI^WT^-TRF1 alone increased the fraction of cells displaying APBs (Fig 1E and 1F, S1B Fig). The co-localizations induced by ATRX deletion appear to be specific to telomeres, because PML co-localization with centromeres was infrequent and not altered by the induction of telomeric DSBs or ATRX deficiency (S1C–S1F Fig). The APBs observed in ATRX-deficient cells with telomeric DSBs appear smaller than those of human ALT cells [5], perhaps due to the short time period in which they are formed in this experimental setting. It is also possible that MEFs generate APBs with slightly different characteristics than those in human cells. Nonetheless, the structures we observe have the critical hallmark of APBs: the association of telomeres with PML bodies.

ATRX deficiency also increased telomere exchanges in response to telomeric DSBs. As shown previously [66], expression of FokI^WT^-TRF1 modestly induced telomere exchanges in ATRX-proficient cells (Fig 1G and 1H). Loss of ATRX significantly increased the frequency of telomere exchanges detectable by chromosome-orientation (CO)-FISH. In this analysis, a chromosome end was scored as positive for a telomere exchange regardless of whether one or both of the sister chromatids displayed the double labeling indicative of HDR. Although telomere sister chromatid exchanges (T-SCEs) likely occur in ATRX-deficient cells containing telomeric DSBs, many chromosome ends displayed an exchange event on only one of the sister telomeres (Fig 1G and 1H, S1G Fig). One possible explanation is that some of the telomere exchanges involved nonallelic telomeres rather than sister telomeres.

BIR events are not detectable by the standard CO-FISH technique, which removes both strands of conservatively replicated DNA. However, the formation of ECTSs is a predicted outcome when BIR of the proximal DNA end formed by FokI-TRF1 cleavage leaves the distal end unrepaired. Loss of ATRX increased the formation of ECTSs in cells with telomeric DSBs (Fig 1G and 1I). The ECTSs represented both leading- and lagging-strand telomeres, as expected for FokI cutting in both sister telomeres. Although we and others have not observed extrachromosomal, circular telomeric DNA with an intact C-rich strand (C-circles) in MEFs [72,73], extrachromosomal, circular telomeric DNA with an intact G-rich strand (G-circles) arose in ATRX-deficient cells after the induction of telomeric DSBs (Fig 1J and 1K). The formation of APBs, the increased telomere exchanges, and the increased abundance of ECTSs and circular telomeric DNA indicate that ATRX loss changes the repair outcomes of DSBs within telomeric DNA.

### Deletion of ATRX causes a telomere-specific cohesion defect

**Fig 2:**
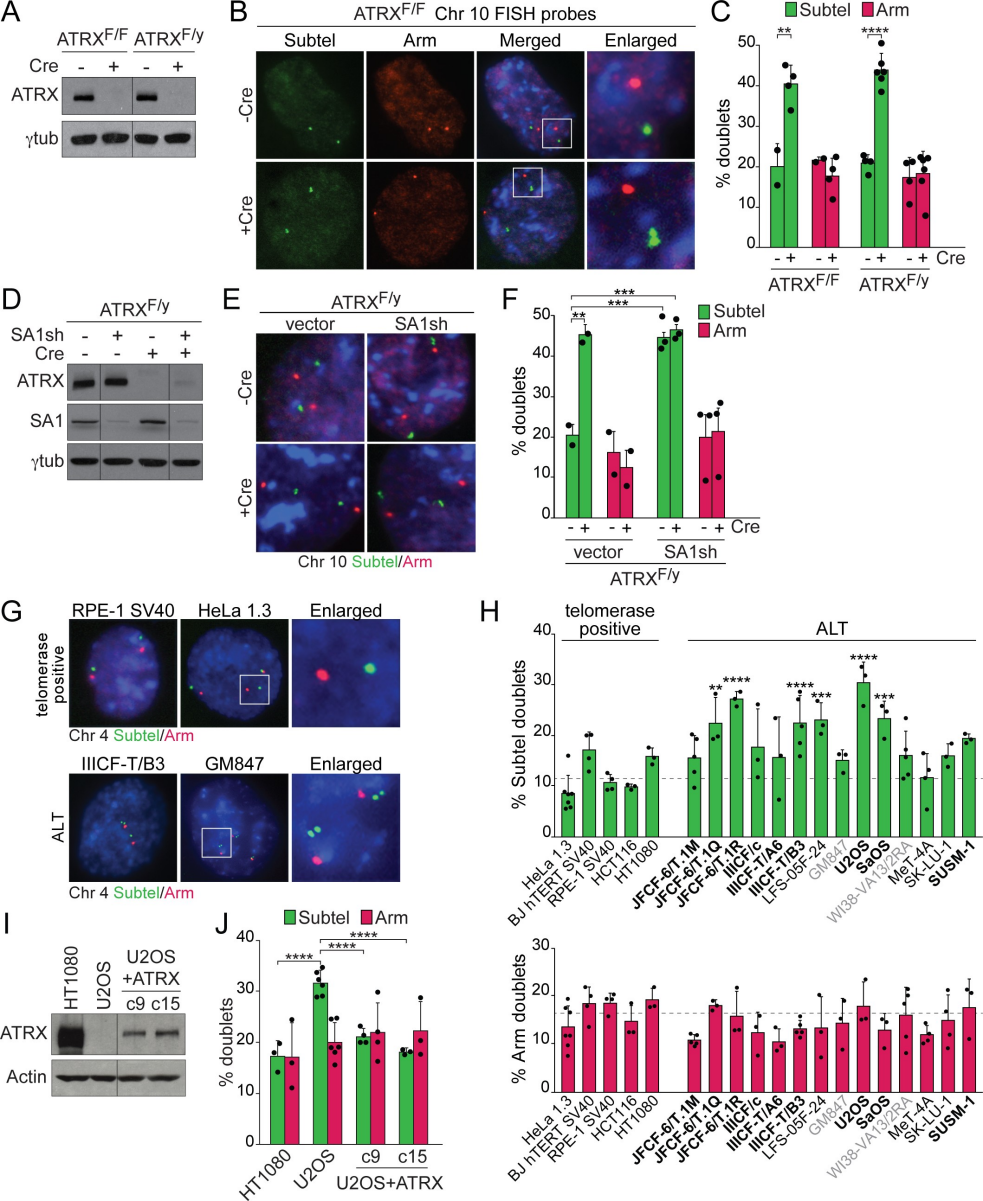
Loss of ATRX causes a telomere-specific cohesion defect. **(A)** Immunoblot showing loss of ATRX in conditional KO MEFs treated with Cre (4 days). γtubulin serves as a loading control. Fragments of the same original image were spliced together to remove unnecessary lanes. **(B)** FISH of the arm (RP23-453P21, red) and subtelomeric (RP23-71E10, green) probes on Chromosome 10 in interphase cells. **(C)** Quantification of the Chromosome 10 FISH signals observed as doublets. Bars from ATRX^F/F^ MEFs: means and SEMs of 2 experiments in which ATRX deletion was mediated by Hit&Run Cre or pWZL-Cre. Bars from ATRX^F/y^ MEFs: means and SDs of at least 4 experiments. **(D)** Immunoblots showing shRNA knockdown of SA1 in ATRX^F/y^ MEFs treated with Cre (4 days). Fragments of the same original image were spliced together to remove unnecessary lanes. **(E)** FISH staining of interphase cells from MEFs described in (D) with probes targeting the arm (red) and subtelomeric (green) regions of Chromosome 10. **(F)** Quantification of the FISH signals observed as doublets. Bars: means and SEMs from 2 experiments utilizing two distinct shRNAs. **(G)** FISH of the arm (RP11-442P12, red) and subtelomeric (RP11-326O23, green) probes on human Chromosome 4 in telomerase-positive and ALT cell lines. **(H)** Quantification of the Chromosome 4 FISH signals observed as doublets. Bars: means and SDs from at least 3 experiments. The *p*-values were derived by comparing each ALT cell line to the collective results of all telomerase-positive cells (dotted line). Bold text denotes ALT cell lines with ATRX mutations, and gray indicates undetectable ATRX protein by immunoblot. **(I)** Immunoblot for ectopic expression of ATRX in 2 U2OS clones. Actin serves as a loading control. Fragments of the same original image were spliced together to remove unnecessary lanes. **(J)** Quantification of the Chromosome 4 FISH signals observed as doublets. Bars: means and SDs of at least 3 experiments collected over approximately 1 month. Pairwise comparisons in panel C were derived from a two-tailed, unpaired *t* test. All other *p*-values were derived from a one-way ANOVA with Tukey or Dunnett’s correction. Symbols as in Fig 1. The underlying numerical data and statistical analysis for each figure panel can be found in S1 Data. ALT, alternative lengthening of telomeres; ATRX, alpha thalassemia/mental retardation syndrome X-linked chromatin remodeler; ATRX^F/F^, female embryo with two floxed ATRX alleles; ATRX^F/y^, male embryo with a single floxed ATRX allele; Cre, recombinase acting on Lox sites; FISH, fluorescence in situ hybridization; KO, knockout; MEF, mouse embryonic fibroblast; pWZL, retroviral vector; SA1, stromal antigen 1; SD, standard deviation; SEM, standard error of the mean; shRNA, short hairpin RNA; U2OS, human osteosarcoma cell line.

A prior report indicated that ATRX diminishes telomere cohesion in mitosis [33], whereas another study documented a minor role for ATRX in promoting cohesion of mitotic chromosomes [74]. We sought to determine the effect of ATRX on telomere cohesion in interphase when DSB repair takes place. To this end, FISH signals from probes specific for the subtelomeric regions of mouse Chromosomes 8 and 10 [57] (and control probes for internal regions of each chromosome arm, S2A Fig) were scored in interphase cells as either a single focus or a doublet. The doublets indicate separation of sister chromatids and thus loss of cohesion. Deletion of ATRX produced a significant increase in telomere doublets with probes for both Chromosomes 8 and 10 in two independent cell lines (Fig 2A–2C, S2B and S2C Fig). Loss of ATRX had no significant effect on the fraction of chromosome arm signals observed as doublets (Fig 2C and S2C Fig). The finding that approximately 40% of telomere FISH signals appear as doublets is consistent with premature sister telomere cohesion loss following replication. Because doublet signals can only be formed after telomere replication, it is expected that the maximal frequency of doublets equals the percentage of cells in S/G2, which is close to 40% (S1A Fig). We therefore conclude that in the absence of ATRX most replicated telomeres lacked cohesion, whereas ATRX had a negligible effect on the cohesion of chromosome arms in interphase.

As expected [57], SA1 depletion also significantly increased the frequency of telomere doublets while having no discernable effect on arm cohesion (Fig 2D–2F). The combined loss of SA1 and ATRX caused no further increase in the frequency of telomere doublets (Fig 2F). However, because both ATRX loss and SA1 depletion lead to the maximal expected level of telomere doublets, the lack of further increase upon combined loss of ATRX and SA1 is not informative with regard to their epistatic relationship.

### Human ALT cells show defects in telomere cohesion

We next examined telomere cohesion in a panel of ALT cell lines and telomerase-positive control cell lines. FISH probes for the subtelomeric or internal arm region of human Chromosome 4 were confirmed to localize appropriately on metaphase spreads (S2G Fig). In interphase cells, the fraction of arm signals observed as doublets was largely consistent between ALT cells and telomerase-positive cells. In contrast, telomere doublets were elevated in many (but not all) ALT cell lines relative to the telomerase-positive controls (Fig 2G and 2H).

Given the variability of the cohesion phenotype in ALT cells and the lack of control cell lines with matching cell type and origin, we directly tested the effect of ATRX on telomere cohesion by depleting ATRX from HeLa 1.3 and reintroducing full-length ATRX into U2OS cells by transfection. Similar to the phenotype observed in MEFs, knockdown of ATRX in HeLa 1.3 cells specifically increased telomere doublets (S2H–S2J Fig). Although it has been reported that reintroduction of ATRX into U2OS impedes proliferation [33], we and others did not observe diminished growth of U2OS cells upon introduction of ATRX [21,75]. Consistent with published reports [19,21], reintroduction of ATRX repressed hallmarks of ALT, as evidenced by a reduction in both C-circles and telomere exchanges (S3A–S3C Fig). Importantly, two independent U2OS clones with stable expression of ATRX showed a significant reduction in telomere cohesion defects compared with the ATRX-deficient U2OS control (Fig 2I and 2J, S3D Fig). These results demonstrate that in both human and mouse cells, loss of ATRX is associated with a telomere-specific cohesion defect.

### Loss of telomere cohesion promotes interactions between nonallelic telomeres

**Fig 3:**
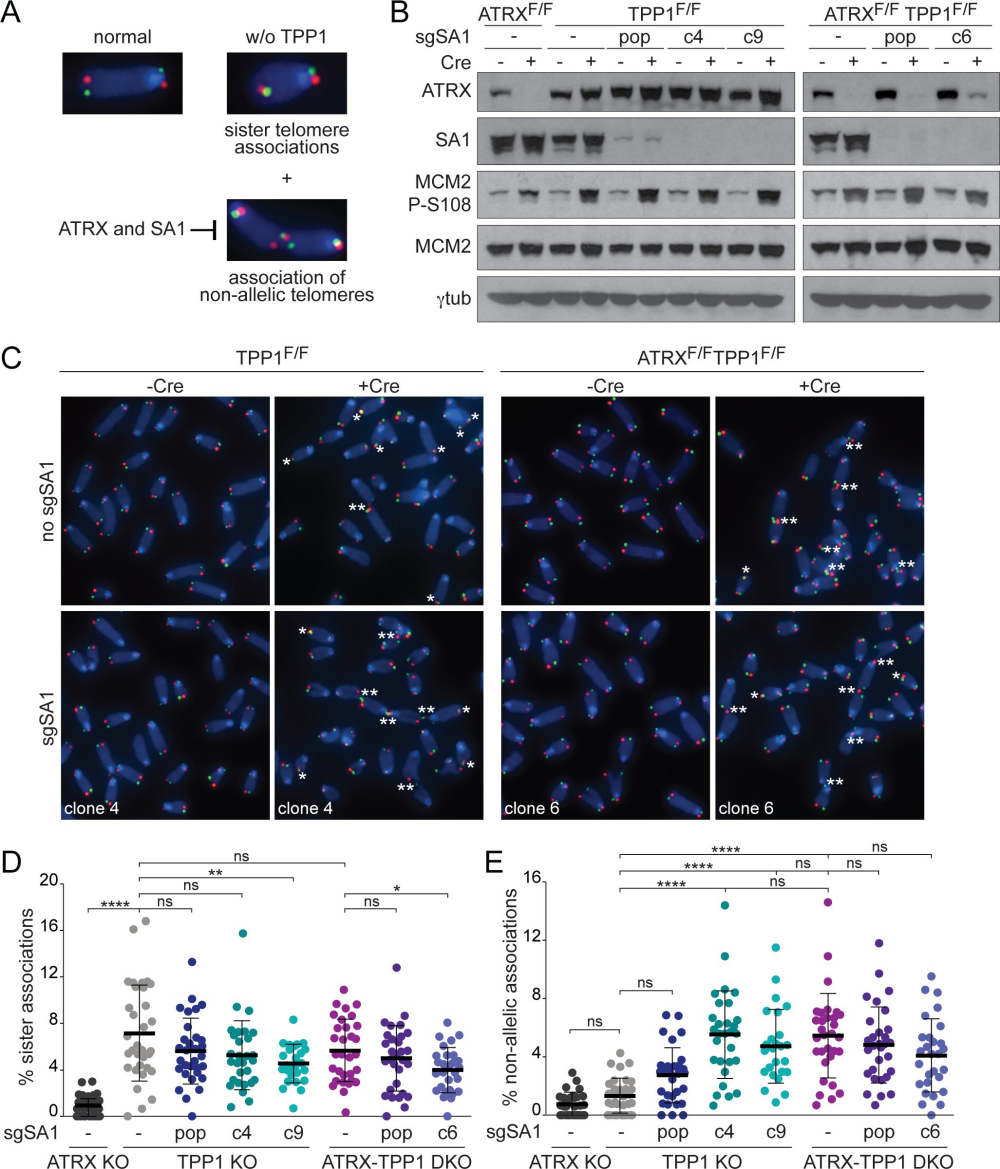
Telomere cohesion defects promote interactions between nonallelic telomeres. **(A)** Representative images of CO-FISH staining on a normal metaphase chromosome, the sister telomere associations frequently observed after deletion of TPP1, and the occasional nonallelic telomere associations observed after TPP1 loss that are suppressed by ATRX- and SA1-mediated telomere cohesion. **(B)** Immunoblots showing Cre-mediated deletion of ATRX (4 days) and SA1 targeting by CRISPR/Cas9 in the indicated MEFs. pop, an SA1-targeted population of cells; c#, SA1 KO clone. Phosphorylation of MCM2 S108 in TPP1-deficient cells is representative of DNA damage signaling and efficient deletion of TPP1. γtubulin serves as a loading control. **(C)** Representative images of CO-FISH staining on metaphase spreads (as in Fig 1G) from the indicated cell lines. Telomere associations are highlighted by asterisks (*, sister association; **, nonallelic association). **(D, E)** Quantification of sister (D) and nonallelic (E) telomere associations in Cre-treated cells detected by CO-FISH. Data points represent the percentage of long arm chromosome ends displaying sister associations and the percentage of all chromatids associated with nonallelic telomeres in one metaphase spread. Bars: means and SDs of >25 metaphases from 3 experiments. All *p*-values were derived from a one-way ANOVA with Tukey correction. Symbols as in Fig 1. The underlying numerical data and statistical analysis for each figure panel can be found in S1 Data. ATRX, alpha thalassemia/mental retardation syndrome X-linked chromatin remodeler; c#, SA1 KO clone number; Cas9, CRISPR associated protein 9; CO-FISH, chromosome orientation fluorescence in situ hybridization; Cre, recombinase acting on Lox sites; CRISPR, clustered regularly interspaced short palindromic repeats; KO, knockout; MCM2, minichromosome maintenance complex component 2; MEF, mouse embryonic fibroblast; ns, not significant; P-S108, phospho-serine 108; pop, SA1-targeted population; SA1, stromal antigen 1; SD, standard deviation; sgSA1, single guide RNA for SA1; TPP1, ACD shelterin complex subunit and telomerase recruitment factor.

Loss of telomere cohesion is predicted to increase the frequency of nonallelic repair of telomeric DSBs, but the sequence identity of all telomeres makes it difficult to develop an assay for recombination between nonallelic telomeres. To monitor the occurrence of physical interactions between nonallelic telomeres, we exploited a specific aspect of the telomere dysfunction phenotype resulting from loss of the ACD shelterin complex subunit and telomerase recruitment factor, TPP1. Deletion of TPP1 results in a prominent sister telomere association phenotype [76], which is scored based on the juxtaposition of the telomeres of the long arms of metaphase chromosomes (Fig 3A). Nonallelic (non-sister) telomere associations are infrequent after TPP1 loss, allowing for detection of an increase in nonallelic telomere interactions following loss of telomere cohesion.

Using MEFs from which ATRX and TPP1 could be individually or jointly deleted, we examined the effect of ATRX loss on the interactions of telomeres lacking TPP1. Telomere associations were infrequent in ATRX-deficient cells (Fig 3B–3E), whereas deletion of TPP1 induced frequent sister telomere associations and only occasional nonallelic telomere associations. Importantly, ATRX deletion significantly increased the nonallelic telomere associations in TPP1 null cells (Fig 3B–3E).

Deletion of SA1 with clustered regularly interspaced short palindromic repeats (CRISPR)/CRISPR associated protein 9 (Cas9; Fig 3B, S4A and S4B Fig) also produced a markedly increased association of nonallelic telomeres in TPP1-deficient cells (Fig 3C–3E). Loss of SA1 did not further increase the frequency of nonallelic telomere associations in ATRX-TPP1 double knockout (DKO) cells (Fig 3E), suggesting ATRX and SA1 function in the same pathway. Similar to ATRX-deficient cells, telomere associations were infrequent in SA1 KO cells but induced after deletion of TPP1 (Fig 3D and 3E, S4C and S4D Fig). These data demonstrate that loss of telomere cohesion can promote interactions between nonallelic telomeres.

### Lack of telomere cohesion explains a subset of the ATRX phenotypes

**Fig 4:**
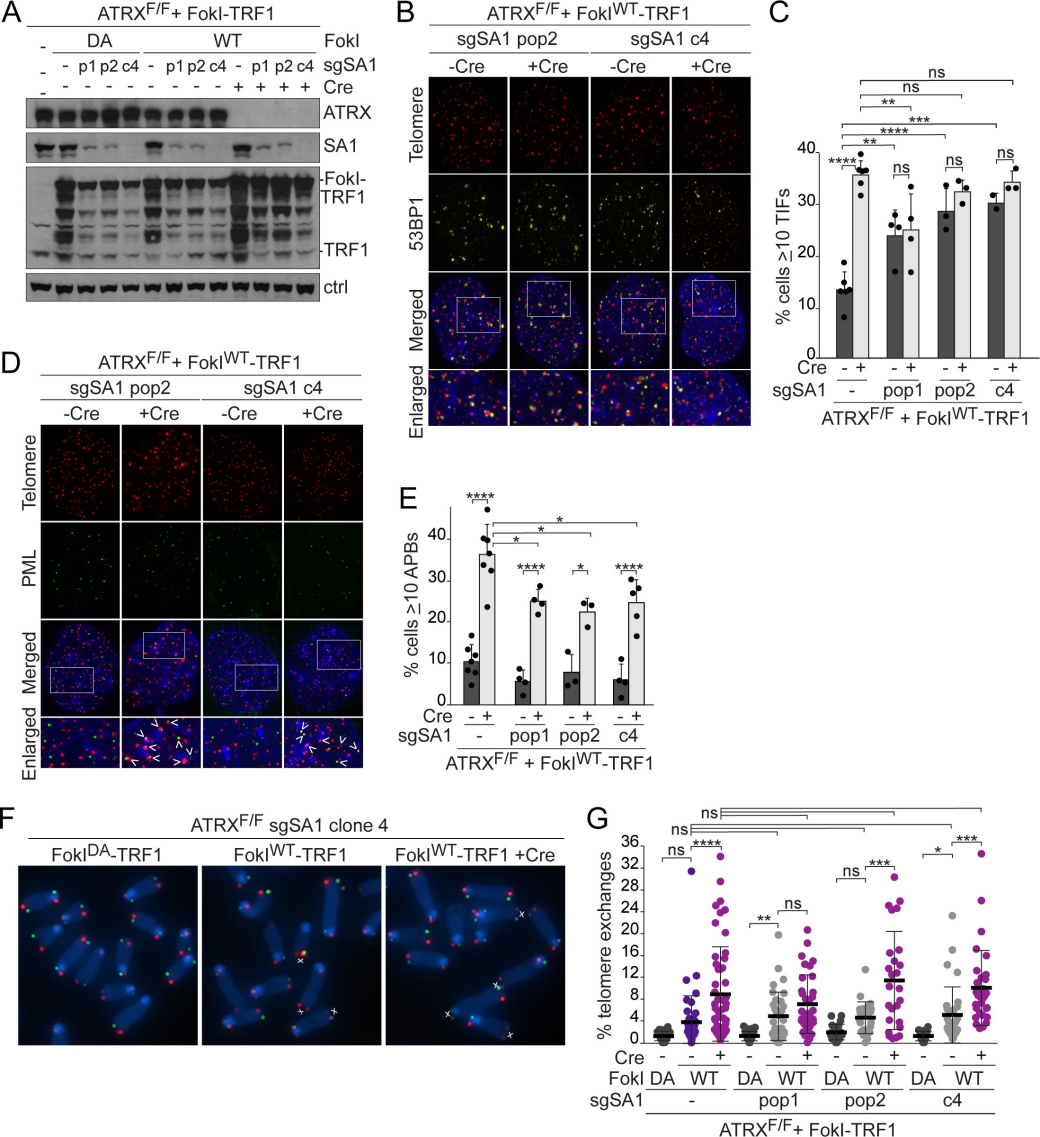
SA1 deletion exacerbates the TIF response to telomeric DSBs but does not induce ALT phenotypes. **(A)** Immunoblots showing expression of FokI-TRF1 (6 days) in control and CRISPR/Cas9-targeted SA1 KO ATRX^F/F^ MEFs infected with Cre (4 days). p1/p2, two separate SA1-targeted populations of cells; c4, SA1 KO clone; Ctrl, nonspecific band used as a loading control. **(B)** TIFs in SA1 KO ATRX^F/F^ MEFs expressing FokI^WT^-TRF1 detected by IF-FISH as in Fig 1C. **(C)** Quantification of the TIF response in FokI^WT^-TRF1–expressing cells, as assayed in (B). Bars: means and SDs from at least 3 experiments of >100 cells each. **(D)** APBs in SA1 KO ATRX^F/F^ MEFs expressing FokI^WT^-TRF1 detected by IF-FISH as in Fig 1E. **(E)** Quantification of the percentage of cells with ≥10 APBs in MEFs expressing FokI^WT^-TRF1, as assayed in (D). Bars: means and SDs from at least 3 experiments of >100 cells each. **(F)** CO-FISH staining of metaphase spreads from SA1 KO c4 ATRX^F/F^ MEFs expressing FokI^WT^-TRF1 as in Fig 1G. **(G)** Quantification of telomere exchanges detected by CO-FISH in control and SA1 KO MEFs expressing FokI-TRF1. Each data point represents the percentage of chromosome ends with telomere exchanges in one metaphase spread. Bars: means and SDs of >25 metaphases from at least 3 experiments. All *p*-values were derived from a one-way ANOVA with Tukey correction. Symbols as in Fig 1. The underlying numerical data and statistical analysis for each figure panel can be found in S1 Data. ALT, alternative lengthening of telomeres; APB, ALT-associated PML body; ATRX^F/F^, female embryo with two floxed ATRX alleles; Cas9, CRISPR associated protein 9; CO-FISH, chromosome orientation fluorescence in situ hybridization; Cre, recombinase acting on Lox sites; CRISPR, clustered regularly interspaced short palindromic repeats; DA, nuclease dead FokI-TRF1; DSB, double-strand break; FISH, fluorescence in situ hybridization; FokI-TRF1, FokI nuclease domain and telomeric repeat binding factor 1 fusion protein; IF, immunofluorescence; KO, knockout; MEF, mouse embryonic fibroblast; ns, not significant; PML, promyelocytic leukemia; SA1, stromal antigen 1; SD, standard deviation; sgSA1, single guide RNA for SA1; TIF, telomere dysfunction–induced foci; WT, wild-type FokI-TRF1; 53BP1, tumor protein p53-binding protein 1.

To determine whether the altered telomeric DSB repair observed in ATRX-deficient MEFs is attributable to the loss of telomere cohesion, we examined the repair outcomes in SA1-deficient cells. Similar to the loss of ATRX, removal of SA1 significantly increased both the percentage of cells with TIFs and the number of TIFs per cell following expression of FokI^WT^-TRF1 (Fig 4A–4C and S5A Fig). Deletion of ATRX did not further increase the telomere damage in SA1 KO cells (Fig 4A–4C). The exacerbated TIF response in SA1 KO and ATRX KO cells after the induction of telomeric DSBs is consistent with reports that cohesion defects reduce the efficiency of DSB repair [41,49,54,77,78].

However, SA1 loss was not sufficient for the induction of APBs or telomere exchanges in response to telomeric DSBs. Deletion of SA1 did not significantly alter the fraction of cells with APBs (Fig 4D and 4E), while deletion of ATRX reproducibly induced APB formation in control and SA1 KO MEFs (Fig 4D and 4E, S5B Fig). The induction of APBs following ATRX deletion was reduced in SA1 KO cells compared with SA1-proficient cells. This effect is likely due to the significant reduction in the number of PML foci per cell in SA1 KO MEFs (S5C Fig). Finally, loss of SA1 did not exacerbate the telomere exchanges associated with expression of FokI^WT^-TRF1 (Fig 4F and 4G), whereas deletion of ATRX significantly increased the frequency of telomere exchanges independent of SA1 status (Fig 4F and 4G). These results suggest that loss of ATRX-mediated telomere cohesion reduces telomeric DSB repair and promotes interchromosomal telomeric interactions; however, loss of cohesion-independent functions of ATRX are required to induce the full repertoire of phenotypes associated with ATRX deletion.

### Telomeric DSB repair is altered by the combined loss of DAXX and telomere cohesion

**Fig 5:**
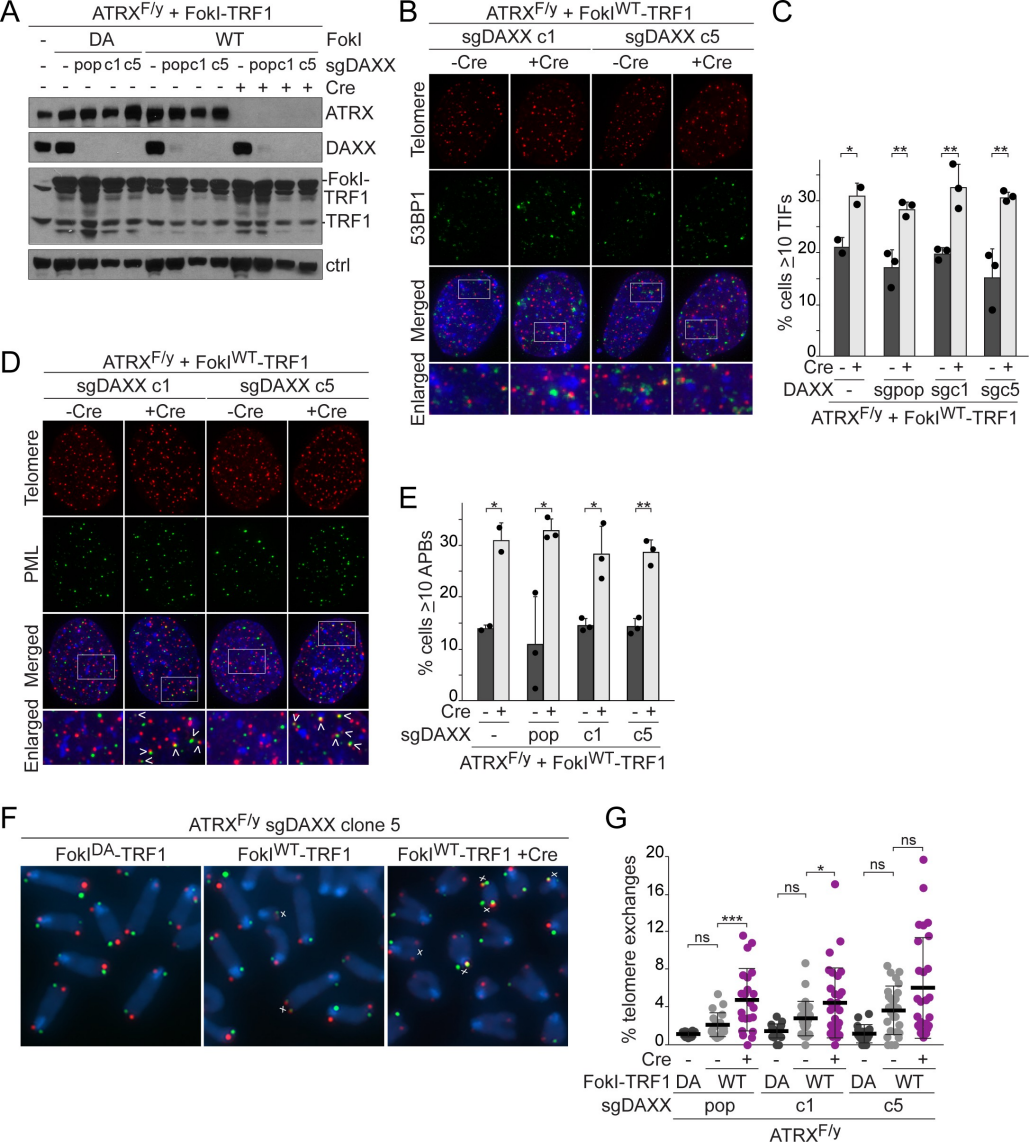
Deletion of DAXX does not alter telomeric DSB repair. **(A)** Immunoblots showing FokI-TRF1 expression (6 days) in control and CRISPR/Cas9-targeted DAXX KO ATRX^F/y^ MEFs treated with Cre (4 days). pop, a DAXX-targeted population of cells; c1/c5, DAXX KO clones; Ctrl, nonspecific band used as a loading control. **(B)** Detection of TIFs in 2 DAXX KO ATRX^F/y^ clones expressing FokI^WT^-TRF1 by IF-FISH as in Fig 1C. **(C)** Quantification of the TIF response in FokI^WT^-TRF1–expressing cells, as assayed in (B). Bars: means and SDs from 3 experiments for DAXX KO MEFs (means and SEMs from 2 experiments for control MEFs) of >100 cells each. **(D)** APBs in 2 DAXX KO ATRX^F/y^ clones expressing FokI^WT^-TRF1 detected by IF-FISH as in Fig 1E. **(E)** Quantification of the percentage of cells with ≥10 APBs in control and DAXX KO MEFs expressing FokI^WT^-TRF1, as assayed in (D). Bars: means and SDs from 3 experiments of >100 cells each for DAXX KO MEFs (means and SEMs from 2 experiments for control MEFs). **(F)** CO-FISH staining of metaphase spreads from DAXX KO c5 ATRX^F/y^ MEFs expressing FokI-TRF1, as in Fig 1G. **(G)** Quantification of telomere exchanges detected by CO-FISH in DAXX KO ATRX^F/y^ MEFs expressing FokI-TRF1. Each data point represents the percentage of chromosome ends with telomere exchanges in one metaphase spread. Bars: means and SDs of >20 metaphases from 2–3 experiments. Pairwise comparisons in panels C and E were derived from a two-tailed, unpaired *t* test. All other *p*-values were derived from a one-way ANOVA with Tukey correction. Symbols as in Fig 1. The underlying numerical data and statistical analysis for each figure panel can be found in S1 Data. APB, ALT-associated PML body; ATRX^F/y^, male embryo with a single floxed ATRX allele; Cas9, CRISPR associated protein 9; CO-FISH, chromosome orientation fluorescence in situ hybridization; Cre, recombinase acting on Lox sites; CRISPR, clustered regularly interspaced short palindromic repeats; DA, nuclease dead FokI-TRF1; DAXX, death domain-associated protein; DSB, double-strand break; FISH, fluorescence in situ hybridization; FokI-TRF1, FokI nuclease domain and telomeric repeat binding factor 1 fusion protein; IF, immunofluorescence; KO, knockout; MEF, mouse embryonic fibroblast; ns, not significant; PML, promyelocytic leukemia; SD, standard deviation; SEM, standard error of the mean; sgDAXX, single guide RNA for DAXX; TIF, telomere dysfunction–induced foci; WT, wild-type FokI-TRF1; 53BP1, tumor protein p53-binding protein 1.

**Fig 6:**
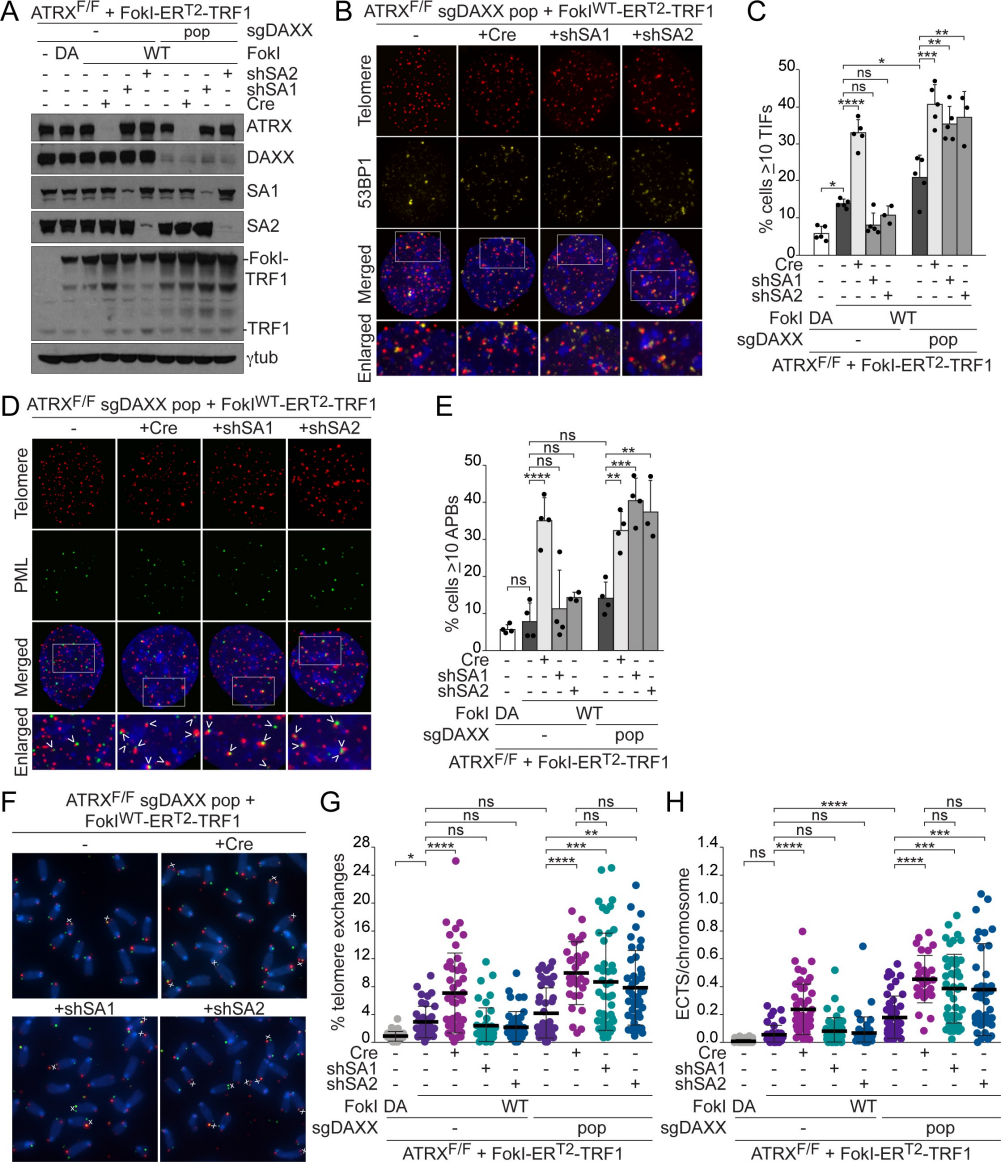
The combined loss of DAXX and telomere cohesion phenocopies ATRX loss. **(A)** Immunoblots showing FokI-ER^T2^-TRF1 expression (6 days) in control and a CRISPR/Cas9-targeted DAXX-deficient population (pop) of ATRX^F/F^ MEFs. Cre-mediated deletion of ATRX and shRNA knockdown of SA1 and SA2 are also shown (4 days). γtubulin serves as a loading control. **(B)** Detection of TIFs in DAXX-targeted cells expressing FokI^WT^-ER^T2^-TRF1 by IF-FISH for 53BP1 and telomeres as in Fig 1C. **(C)** Quantification of the TIF response in FokI-ER^T2^-TRF1–expressing cells, as assayed in (B). Bars: means and SDs from at least 3 experiments of >100 cells each. **(D)** APBs in DAXX-targeted MEFs expressing FokI^WT^-ER^T2^-TRF1 detected by IF-FISH as in Fig 1E. **(E)** Quantification of the percentage of cells with ≥10 APBs in cells expressing FokI-ER^T2^-TRF1, as assayed in (D). Bars: means and SDs from at least 3 experiments of >100 cells each. **(F)** CO-FISH staining of metaphase spreads from DAXX-targeted ATRX^F/F^ MEFs expressing FokI^WT^-ER^T2^-TRF1 as in Fig 1G. **(G)** Quantification of telomere exchanges detected by CO-FISH in control and DAXX-deficient ATRX^F/F^ MEFs expressing FokI-ER^T2^-TRF1. Each data point represents the percentage of chromosome ends with telomere exchanges in one metaphase spread. Bars: means and SDs of >40 metaphases from 5 experiments (29 metaphases from 3 experiments for sgDAXX pop+Cre). **(H)** Quantification of the ECTSs from metaphase spreads described in (F, G). Each data point represents the number of ECTSs per chromosome in one metaphase spread. All *p*-values were derived from a one-way ANOVA with Tukey correction. Symbols as in Fig 1. The underlying numerical data and statistical analysis for each figure panel can be found in S1 Data. APB, ALT-associated PML body; ATRX, alpha thalassemia/mental retardation syndrome X-linked chromatin remodeler; ATRX^F/F^, female embryo with two floxed ATRX alleles; Cas9, CRISPR associated protein 9; CO-FISH, chromosome orientation fluorescence in situ hybridization; Cre, recombinase acting on Lox sites; CRISPR, clustered regularly interspaced short palindromic repeats; DA, nuclease dead FokI-ER^T2^-TRF1; DAXX, death domain-associated protein; ECTS, extrachromosomal telomeric signal; FISH, fluorescence in situ hybridization; FokI-ER^T2^-TRF1, tamoxifen-inducible nuclear localized FokI-TRF1 fusion protein; IF, immunofluorescence; MEF, mouse embryonic fibroblast; ns, not significant; PML, promyelocytic leukemia; SA1, stromal antigen 1; SA2, stromal antigen 2; SD, standard deviation; sgDAXX, single guide RNA for DAXX; shRNA, short hairpin RNA; shSA1, short hairpin RNA for SA1; shSA2, short hairpin RNA for SA2; TIF, telomere dysfunction–induced foci; WT, wild-type FokI-ER^T2^-TRF1; 53BP1, tumor protein p53-binding protein 1.

Because SA1 deficiency did not fully reproduce the phenotypes observed after ATRX deletion, we examined the combined contribution of DAXX and cohesion defects to the repair of telomeric DSBs. Deletion of DAXX alone did not appear to substantially alter the repair of telomeric DSBs. The absence of DAXX, either due to CRISPR/Cas9 editing in clones or in a population of cells (Fig 5A, S6A and S6B Fig), had no effect or a minimal effect on the percentage of cells with TIFs and the number of TIFs per cell following expression of FokI^WT^-TRF1 (Figs 5B, 5C, and 6C, S6C Fig). Furthermore, deletion of DAXX did not obviously increase the fraction of cells with APBs (Figs 5D, 5E and 6E) or the frequency of telomere exchanges (Figs 5F, 5G and 6G). Deletion of ATRX from DAXX-deficient cells reproducibly exacerbated the telomere damage phenotype, promoted APB formation, and elevated telomere exchanges (Figs 5C, 5E, 5G, 6C, 6E, 6G and S6C–S6E Fig). Furthermore, DAXX deletion did not induce telomere cohesion defects in interphase cells, nor did it promote nonallelic telomere associations after loss of TPP1 (S7 Fig). These results indicate that ATRX promotes telomere cohesion independently of DAXX and that loss of DAXX alone does not alter the repair of telomeric DSBs.

We next treated a DAXX-deficient population of ATRX^F/F^ MEFs with short hairpin RNA (shRNA) to SA1 to disrupt replication-established telomere cohesion or with an shRNA to SA2 to prevent damage-induced cohesion at telomeric DSBs (Fig 6A). Knockdown of SA1 in DAXX-proficient cells did not exacerbate the TIF response to tamoxifen-inducible nuclear localized FokI-TRF1 fusion protein (FokI^WT^-ER^T2^-TRF1), in contrast to what was observed in the SA1 CRISPR KO cells (Fig 4C), likely due to partial shRNA-mediated knockdown. The SA2 shRNA also had no effect on the TIF response in the presence of DAXX. However, knockdown of SA1 or SA2 elevated the percentage of cells with TIFs in DAXX-deficient cells containing telomeric DSBs, and did so to a similar extent as that observed with ATRX deletion (Fig 6B and 6C). Importantly, the combination of DAXX deficiency and depletion of either SA1 or SA2 also promoted APB formation in cells with telomere DSBs (Fig 6D and 6E, S6F Fig). Furthermore, CO-FISH showed that the combined loss of DAXX and either SA1 or SA2 increased telomere exchanges (Fig 6F and 6G). While DAXX deletion alone appears sufficient to elevate ECTSs in cells containing telomeric DSBs, the frequency of ECTSs is further increased by deletion of ATRX or depletion of either SA1 or SA2 (Fig 6F and 6H). Thus, in the absence of DAXX, a telomere cohesion defect produced the full spectrum of events associated with telomeric DSBs in ATRX-deficient cells: delayed telomeric DSB repair, APBs, telomere exchanges, and an increase in ECTSs. These data suggest that ATRX controls telomeric DSB repair through the combination of DAXX- and cohesion-mediated activities.

## Discussion

**Fig 7:**
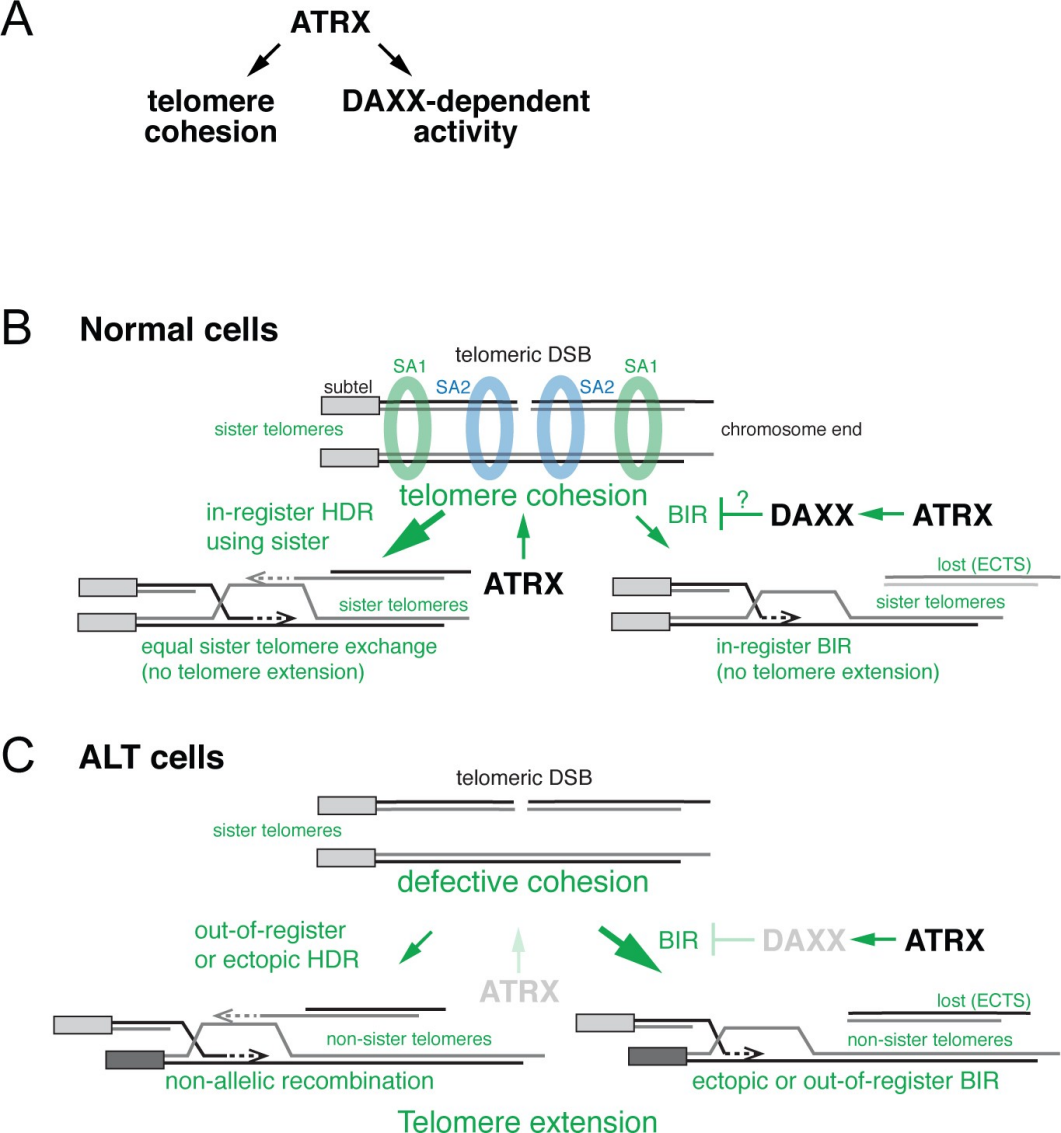
Model for how loss of ATRX promotes ALT. **(A)** Proposed role for ATRX in repressing ALT through telomere cohesion and a DAXX-mediated activity. **(B)** Repair of telomeric DSBs in cells with intact ATRX and DAXX. Telomere cohesion is established during replication in an SA1-dependent manner and at a DSB by SA2-dependent cohesion. The close proximity and proper alignment of the chromatids by cohesin favors use of the sister telomere for DSB repair and ensures that repair results in equal sister telomere exchange or BIR using the sister telomere in-register, thus providing no mechanism for telomere extension. **(C)** ATRX loss changes telomeric DSB repair and promotes ALT through two separate pathways. First, the disruption of ATRX-mediated telomere cohesion allows unequal sister telomere recombination and the use of nonallelic telomeres for DSB repair. These types of interactions are necessary for telomere elongation and are consistent with the recombination events observed in ALT cells. Second, disruption of a DAXX-dependent function of ATRX is proposed to change the repair outcomes of telomeric DSBs, promoting BIR over other DSB repair pathways. ALT, alternative lengthening of telomeres; ATRX, alpha thalassemia/mental retardation syndrome X-linked chromatin remodeler; BIR, break-induced replication; DAXX, death domain-associated protein; DSB, double-strand break; HDR, homology-directed repair; SA1, stromal antigen 1; SA2, stromal antigen 2.

Loss of function mutations in ATRX are the predominant genetic alteration in ALT cells. Here, we report that ATRX controls the fate of DSBs in telomeric DNA. In the absence of ATRX, telomeres with DSBs are repaired less efficiently, show increased interactions with PML bodies, give rise to more extrachromosomal telomeric DNA (a predicted outcome of BIR (Fig 7)), and show a higher rate of HDR as evidenced by telomere sequence exchanges. We further show that ATRX controls DSB repair within telomeres through two distinct pathways: the establishment and/or maintenance of telomere cohesion and a DAXX-dependent function (Fig 7A).

ATRX depletion was previously shown to give rise to a mild centromeric cohesion defect in mitotic cells [74]. However, our data reveal that ATRX is required for the cohesion of replicated telomeres in human and mouse cells in interphase. Lack of telomere cohesion is also frequent in ATRX-deficient ALT cells and could be restored by introduction of exogenous ATRX.

At post-replicative DSBs, both replication-established and damage-induced cohesion are required for efficient repair, and promote the use of the sister chromatid versus a homologous chromosome as the template for DNA repair [41,42,44–46,50,54,55]. Accordingly, telomere cohesion defects resulting from loss of ATRX or SA1 diminished the efficiency of telomeric DSB repair and increased inappropriate interactions between nonallelic telomeres. The overall increase in telomere exchanges that we observe with cohesion defects (ATRX loss or SA1 loss in the context of DAXX deficiency) is consistent with our assay measuring sister chromatid recombination resulting from both in-register and out-of-register telomere recombination, and possibly interchromosomal telomere recombination. Furthermore, if telomere cohesion indeed prevents telomeric DSBs from undergoing such ectopic interactions, as well as out-of-register interactions with the sister telomere, then loss of cohesion at telomeres lacking ATRX can potentially explain ALT (Fig 7B and 7C). The altered behavior of telomeric DSBs resulting from cohesion defects is predicted to promote ALT by permitting net telomere elongation. For instance, a BIR-like pathway using the sister telomere as an in-register template will not result in net telomere elongation, whereas BIR-mediated repair using the sister telomere out-of-register or a non-sister telomeric template will lead to telomeric DNA extension (Fig 7B and 7C).

We have not been able to establish the mechanism by which ATRX promotes telomere cohesion. ATRX has no reported physical interaction with SA1, and telomeric chromatin immunoprecipitation (ChIP) showed no change in the association of SA1 or shelterin with telomeres in cells lacking ATRX (S2D–S2F Fig). Potentially, ATRX could have a role in establishing cohesion between sister telomeres rather than promoting the association of cohesin proteins with telomeric DNA. ATRX is required for SMC1 occupancy at certain non-telomeric loci [24,79], but whether SMC1 is relevant to telomere cohesion in interphase is unclear.

ATRX was also reported to promote the resolution of sister telomeres in mitosis [33]. The authors suggested that persistent telomere cohesion in mitosis promotes recombination between sister telomeres and thus ALT. However, it is unclear how such recombination would lead to ALT because persistent sister telomere cohesion should repress the type of recombination or BIR events that can elongate telomeres.

Our data show that loss of telomere cohesion is not sufficient to fully reproduce the effects of ATRX deficiency on the repair of telomeric DSBs. We identify a DAXX-dependent function of ATRX as the cooperating event. The combined loss of DAXX and telomere cohesion results in the full spectrum of telomeric DSB repair phenotypes associated with ATRX deletion. Potentially, the formation of H3.3 containing nucleosomes by ATRX/DAXX during the repair of telomeric DSBs could alter repair pathway choices. In this regard, it is noteworthy that the absence of ATRX, DAXX, or H3.3 in G2 diminishes long tract gene conversion (LTGC) at non-telomeric DSBs [80]. Because LTGC competes with other HDR pathways, including BIR, the loss of DAXX may result in more frequent BIR of telomeric DSBs (Fig 7B and 7C). In combination with loss of cohesion at telomeric DSBs, DAXX deficiency could promote the type of BIR that can elongate telomeres.

While DAXX deletion is observed in some cancers with ALT, it is a relatively rare event [12,14–16,18,20,81]. This is consistent with our finding that ATRX has both DAXX-dependent and DAXX-independent roles in controlling DSB repair pathway choice at telomeres. Our findings predict that cell lines and tumors that have developed ALT due to loss of DAXX may also have a deficiency in telomere cohesion or the generation of cohesion at DSBs. Consistent with this proposal, a search of cancer genomics datasets revealed a co-occurrence of AT-rich interaction domain 1A (ARID1A) mutations with DAXX mutations or copy number alterations in glioma and pancreatic neuroendocrine tumors (PanNETs), among others [82–85]. ARID1A inactivation was recently found to induce telomere damage and defects in telomere cohesion due to loss of SA1 [86]. Although sequencing data have not identified mutations in cohesin subunits, transcriptional inactivation could clearly be one avenue through which telomere cohesion defects arise in DAXX-deficient tumors.

In summary, we speculate that ALT is promoted by ATRX loss because of the resulting diminished cohesion between the telomeric DSB and the sister telomere. This loss of cohesion is needed to allow the telomeric DSB to interact with nonallelic telomeric DNA or with the sister telomere in an out-of-register manner such that BIR (or other forms of HDR) can extend telomere length. We further speculate that there is a second requirement for ALT, assigned to loss of DAXX or ATRX, that leads to a shift towards BIR over other forms of DSB repair. Although BIR is the simplest way to explain the net increase in telomeric DNA needed for ALT, it remains to be determined whether it is the only pathway for the maintenance of telomeres in ALT cells. A further change that is likely to be required to allow cells using the ALT pathway to escape telomere crisis is a deficiency in the cyclic GMP-AMP synthase (cGAS)–stimulator of interferon genes (STING) pathway. Because ALT generates extrachromosomal DNA (e.g., ECTSs) that will frequently become cytoplasmic after mitosis, ALT cells are likely to require loss of the cGAS-STING pathway that is activated by cytoplasmic DNA [87]. This may explain why the deletion of ATRX by itself does not readily induce ALT in cell culture models [17,21,88,89].

## Materials and methods

### Cell culture and viral transduction

The conditional ATRX KO mice have been described previously [90]. Compound genotypes were generated by intercrossing ATRX^F/F^ with TPP1^F/+^ mice [76]. Primary MEFs were isolated from E13.5 embryos and immortalized at passage three with pBabeSV40-LT (a gift from Greg Hannon). Genotyping was performed by Transnetyx (Cordova, TN) and sex determination for ATRX MEFs was performed by PCR using multiplexed primer pairs for *Sry* and *Myog* [91]. ALT cell lines used in this study have been described previously [17].

Cre recombinase was introduced by three retroviral (pMMP) infections with Hit&Run Cre at 12–14-hour intervals. Time points are indicated in days, with time zero set at 12 hours after the first infection. FokI-TRF1 and FokI-ER^T2^-TRF1 were introduced by three retroviral (pLPC or pLPC+blasticidin resistance gene) infections and selected with 2 μg/mL puromycin or 6 μg/mL blasticidin. FokI-TRF1–expressing cells were harvested approximately 6 days after selection. FokI-ER^T2^-TRF1 was induced with 1 μM 4-hydroxytamoxifen (4-OHT) for 2 hours; the cells were washed twice with PBS and harvested 24 hours after removal of 4-OHT, unless otherwise stated. Mouse SA1 and SA2 shRNAs (MilliporeSigma, Burlington, MA; TRCN0000109016, TRCN0000448553, TRCN0000108979) were introduced by 2 lentiviral (pLKO.1) infections at 24-hour intervals and selected in 2 μg/mL puromycin (BAC FISH), or 3 lentiviral infections at 12-hour intervals without selection (DAXX KO cells). Human ATRX (MilliporeSigma; TRCN0000013590, TRCN0000013592) and SA1 shRNAs (MilliporeSigma; TRCN0000140749, TRCN0000145197) were introduced by 3 lentiviral (pLKO.1) infections at 12-hour intervals and harvested at 3 days without selection. Stable expression of full-length ATRX (pEGFP-ATRX-HA) was achieved by transfection (FuGENE HD), followed by selection and maintenance in 500 μg/mL G418.

### CRISPR/Cas9 genomic editing

The target sequence for CRISPR/Cas9-mediated DAXX KO was identified using Benchling (https://benchling.com): sgDAXX-C2, 5′-CAATGATGCTGTCATCGG-(PAM)-3′. The target sequence was incorporated into the *BsmBI*-linearized lentiCRISPRv2 plasmid [92] and co-transfected with pPAX2 and pCMV-VSV-G into 293(F)T packaging cells using calcium phosphate precipitation. Supernatants were collected 46 hours after transfection and filtered, mixed 1:1 with growth media, and supplemented with 4 μg/mL polybrene. Target cells were infected two times at 12-hour intervals and selected with 2 μg/mL puromycin. Populations and clones (isolated by limiting dilution) surviving selection were screened for DAXX deletion by immunoblotting. TOPO-cloned (Thermo Fisher Scientific, Waltham, MA) PCR products were sequenced to characterize the edited alleles (DAXX forward primer: 5′-CAGAGCTGCTCGAAACTGAG-3′, DAXX reverse primer: 5′-ACGCCATCATGTGGTTGG-3′). A second DAXX-targeted population was generated by electroporation of target cells with sgDAXX-C2 incorporated into the *BbsI*-linearized pU6-(BbsI)_CBh-Cas9-T2A-mCherry plasmid (Addgene, Cambridge, MA, 64324) using the Amaxa MEF2 kit (Lonza, Basel, Switzerland). Cells were allowed to recover for 48 hours and subjected to a second round of electroporation. A bulk population was isolated by fluorescence-activated cell sorting (FACS) and screened for DAXX depletion by immunoblotting.

The target sequences for CRISPR/Cas9-mediated SA1 KO were also identified using Benchling: sgSA1-C6, 5′-GGATGGACTCTTCACGAT-(PAM)-3′ and sgSA1-C8, 5′-TGGTCTGTACAATTCGTC-(PAM)-3′. The target sequences were incorporated into the *BbsI*-linearized pU6-(BbsI)_CBh-Cas9-T2A-mCherry plasmid (Addgene, 64324) and introduced into target cells by electroporation, as described above. Clones and bulk populations were isolated by FACS and screened for SA1 deletion by immunoblotting. TOPO-cloned (Thermo Fisher Scientific) PCR products were sequenced to verify gene targeting (SA1 forward primer: 5′-TCCCAGTTAATACAGAGCTGTCC -3′, SA1 reverse primer: 5′-CCAGGTTCCCAGTATAATTCAA-3′).

### Immunoblotting

Cells were lysed in 2X Laemmli buffer (100 mM Tris-HCl, pH 6.8, 20% glycerol, 4% SDS, 0.01% bromophenol blue, 2.5% β-mercaptoethanol), sheared with a 28 gauge insulin needle, and denatured at 100°C. Lysates were resolved by SDS/PAGE and processed for immunoblotting. The following primary antibodies were used: ATRX (Santa Cruz Biotechnology, Dallas, TX, H-300 or Abcam, ab97508), DAXX (MilliporeSigma, D7810), SA1 (Abcam, ab4457), SA2 (Abcam, ab201451), TRF1 (de Lange #1449), MCM2 (Abcam, ab4461), Phospho-MCM2 S108 (Bethyl Laboratories, Montgomery, TX, A300-094A), γtubulin (MilliporeSigma, GTU-88), Actin (Santa Cruz Biotechnology, I-19), and HA.11 (BioLegend, San Diego, CA, 901514).

### BAC probes and FISH

BAC clones (Children’s Hospital Oakland Research Institute, Oakland, CA) from a mouse library (RP23-310L10, RP23-326G18, RP23-453P21, and RP23-71E10) or a human library (RP11-442P12 and RP11-326O23) were used to generate chromosome-specific arm and subtelomere probes for FISH analyses. Probes were labeled by nick translation with biotin-16-dUTP or digoxigenin-11-dUTP (Roche, Basel, Switzerland). Cells were harvested by trypsinization, fixed in methanol/acetic acid (3:1), and dropped onto glass slides. RNase-treated slides were dehydrated consecutively in 70%, 90%, and 100% ethanol and allowed to air dry. Cells were denatured by heating the slides for 2 minutes on an 80°C heat block, and dehydrated in a series of ethanol washes, as above. Slides were incubated with hybridizing solution (2× SSC, 10% dextran sulfate, 50% formamide, containing 200 ng of each labeled BAC probe mixed with 8 μg Cot-1 DNA [Thermo Fisher Scientific]) at 37°C for 16–18 hours and washed with 1× SSC at 60°C. Signals were detected with FITC-conjugated avidin and rhodamine-conjugated anti-digoxigenin antibodies. FISH signals from probes specific for the chromosome arm were scored in all cells, while subtelomeric FISH signals were scored only in cells where all chromosome arm probes displayed a single focus (for the purpose of analyzing premature sister telomere cohesion loss).

### IF-FISH

IF-FISH with 53BP1 (Abcam, ab175933), PML (MilliporeSigma, 05–718), or ACA (anti-centromere antibody, Antibodies, Davis, CA, 15–235) was performed as described previously [93], with minor modifications. After the secondary antibody incubation, cells were fixed in 3% paraformaldehyde/2% sucrose for 10 minutes. Hybridizing solution containing the PNA probe Cy3-OO-(CCCTAA)_3_ or Alexa 647-OO-(TTAGGG)_3_ (PNA Bio, Newbury Park, CA) was added to each coverslip and the cells were denatured for 5 minutes. Co-localization of foci was quantified by an automated foci and co-localization analysis macro-generated by Leonid Timashev (Rockefeller University), as described previously [66]. Briefly, user-entered contrasting and threshold values are applied to all samples within an experiment. After foci are identified in the channels of interest, they are overlaid, and co-localization is determined based on the overlap of a minimum and maximum number of pixels (default 2–1,000). The foci and co-localizations meeting the criteria are overlaid onto DAPI staining, and only those within the nucleus are scored.

### CO-FISH

CO-FISH was performed as described previously [66], with minor modifications. Cells were labeled with BrdU/BrdC for 12–14 hours and digested with 800 U Exonuclease III (Promega, Madison, WI) for 10 minutes at 37°C and 10 minutes at rt. Slides were hybridized with an Alexa 647-OO-(TTAGGG)_3_ PNA probe followed by a Cy3-OO-(CCCTAA)_3_ PNA probe in hybridization solution.

### Analysis of telomeric DNA by in-gel hybridization

Mouse genomic DNA was analyzed as previously described [94]. The single-stranded telomere signals in the native gel were quantified with ImageQuant TL 8.1 (GE Healthcare Life Sciences, Marlborough, MA) software and the ratio of short versus bulk telomeric signals (regions denoted by boxes in Fig 1B) are reported relative to cells expressing FokI^DA^-TRF1 (set at 100).

### G-circle assay

DNA was isolated from cells using the Quick-genomic DNA Miniprep Kit (Zymo Research, Irvine, CA), digested with *HinfI*, and quantified by Hoechst fluorimetry. Thirty nanograms DNA (10 μL) was combined with 30 μL of reaction buffer (containing 0.2 mg/mL BSA, 0.1% Tween 20, 1 mM each dATP, dCTP, dTTP, 1× phi29 buffer, 7.5 U phi29) and incubated at 30°C for 16 hours followed by 20 minutes at 65°C. Reaction products were electrophoresed in 0.6% agarose (Lonza)-0.5× TBE at 50 V for 16 hours. The gel was dried under vacuum for 1 hour at rt and >1 hour at 50°C. G-circle products were hybridized with a γ^32^P-ATP end-labeled [TTAGGG]_4_ telomeric probe. The signals were quantified with ImageQuant TL 8.1 software and are reported relative to the U2OS ALT cell line (set at 100).

### FACS analysis

MEFs were pulsed with 10 μM BrdU (MilliporeSigma) for 30 minutes, harvested by trypsinization, fixed in 70% ethanol, and stored at −20°C. Fixed cells were then denatured with 2N HCl for 30 minutes, stained with FITC-conjugated anti-BrdU antibodies (BD Biosciences, San Jose, CA) according to the manufacturer’s instructions, and treated with 5 μg/mL propidium iodide in PBS. Flow cytometry was performed with a BD Accuri C6 (BD Biosciences) and data were analyzed with FlowJo v10.5 (www.flowjo.com).

### Statistics and reproducibility

Datasets were analyzed using Prism 8 (www.graphpad.com). Pairwise comparisons were derived from a two-tailed, unpaired *t* test. All other *p*-values were derived from a one-way ANOVA with Tukey or Dunnett’s correction. Error bars represent SD unless otherwise noted. Significance levels are given as follows: ****p < 0.0001, ***p < 0.001, **p < 0.01, *p < 0.05, ns (not significant): *p* > 0.05. Experiments were repeated at least three times, except where otherwise stated, with similar results. The underlying numerical data and statistical analysis for each figure panel are available in S1 Data.

## Supporting information

S1 Raw Images Original (unadjusted and uncropped) image files for all immunoblots and gels depicted in each figure panel. (PDF)

S1 Data Excel spreadsheet containing, in separate sheets, the underlying numerical data and statistical analysis for each figure panel. (XLSX)

S1 Fig Characterization of FokI-TRF1–induced telomeric damage after ATRX deletion. **(A)** FACS profiles of ATRX^F/F^ MEFs treated with BrdU 4 days after Cre to monitor the S phase index. **(B)** Quantification of PML foci per cell in ATRX^F/F^ MEFs, as assayed in Fig 1E. Bars: means and SDs of >400 cells. **(C)** Co-localizations in ATRX^F/F^ MEFs detected by IF-FISH for PML (IF, green) and telomeres (FISH, red), and IF for PML (green) and centromeres (anti-centromere antibody, ACA, red). DNA was stained with DAPI. **(D)** Quantification of the fraction of telomeres co-localizing with ACA, as assayed in (C). Each data point represents the fraction of telomeres co-localizing with ACA in one cell. Bars: means and SDs of >300 cells. **(E-F)** Quantification of the percentage of cells with ≥10 PML-ACA (E) or PML-TelC (F) co-localizations, as assayed in (C). Bars: means and SDs of 3 experiments of >100 cells each. **(G)** Quantification of chromosome ends with reciprocal or single telomere exchanges in ATRX^F/F^ MEFs detected by CO-FISH, from Fig 1G. Pairwise comparisons in panel G were derived from a two-tailed, unpaired *t* test. All other *p*-values were derived from a one-way ANOVA with Tukey correction. Symbols as in Fig 1. The underlying numerical data and statistical analysis for each figure panel can be found in S1 Data. ATRX, alpha thalassemia/mental retardation syndrome X-linked chromatin remodeler; ATRX^F/F^, female embryo with two floxed ATRX alleles; BrdU, bromodeoxyuridine; Cre, recombinase acting on Lox sites; FACS, fluorescence-activated cell sorting; FISH, fluorescence in situ hybridization; FokI-TRF1, FokI nuclease domain and telomeric repeat binding factor 1 fusion protein; IF, immunofluorescence; MEF, mouse embryonic fibroblast; PML, promyelocytic leukemia; SD, standard deviation; TelC, C-rich telomere probe [CCCTAA]_3_
(TIF)

S2 Fig Shelterin and SA1 remain associated with telomeric DNA despite loss of cohesion. **(A)** FISH of the arm (red) and subtelomeric (green) probes for mouse Chromosomes 10 and 8 on metaphase spreads from ATRX conditional KO MEFs. **(B)** FISH of the arm (red) and subtelomeric (green) probes on Chromosome 8 in interphase cells from MEFs described in Fig 2A. **(C)** Quantification of the Chromosome 8 FISH signals observed as doublets. Bars: means and SDs of 3 experiments. **(D)** Telomeric ChIP in ATRX^F/F^ MEFs expressing Flag-HA_2_-TPP1 (anti-Flag) and Myc-POT1 (anti-Myc). PI, pre-immune serum. **(E)** Immunoblots for ATRX and TPP1 (HA) 4 d after Hit&Run Cre or approximately 40 PDs after pWZL-Cre. γtubulin serves as a loading control. **(F)** Quantification of the telomeric ChIP signals from 2–3 independent experiments, as assayed in (D). Bars: means and SEMs. The background (PI) was subtracted and ChIP signals were normalized to the input. **(G)** FISH of the arm (red) and subtelomeric (green) probes on human Chromosome 4 in a metaphase spread from HeLa 1.3 cells. **(H)** Immunoblot for ATRX and SA1 after shRNA depletion in HeLa 1.3. γtubulin serves as a loading control. **(I)** FISH of the arm (red) and subtelomeric (green) probes on Chromosome 4 in interphase cells described in (H). **(J)** Quantification of the Chromosome 4 FISH signals observed as doublets. Bars: means and SEMs from 2 independent shRNAs for both ATRX and SA1. Pairwise comparisons in panel (C) were derived from a two-tailed, unpaired *t* test. All other *p* values were derived from a one-way ANOVA with Tukey correction. Symbols as in Fig 1. The underlying numerical data and statistical analysis for each figure panel can be found in S1 Data. ATRX, alpha thalassemia/mental retardation syndrome X-linked chromatin remodeler; ATRX^F/F^, female embryo with two floxed ATRX alleles; ChIP, chromatin immunoprecipitation; Cre, recombinase acting on Lox sites; FISH, fluorescence in situ hybridization; Flag-HA_2_-TPP1, epitope-tagged ACD shelterin complex subunit and telomerase recruitment factor; KO, knockout; MEF, mouse embryonic fibroblast; Myc-POT1, epitope-tagged protection of telomeres 1; PD, population doubling; PI, pre-immune serum; pWZL, retroviral vector; SA1, stromal antigen 1; SD, standard deviation; SEM, standard error of the mean; shRNA, short hairpin RNA.
(TIF)

S3 Fig Repression of ALT hallmarks in U2OS by re-introduction of full-length ATRX. **(A)** Representative dot blot detecting C-circles with an end-labeled ^32^P-[CCCTAA]_4_ probe, and quantification of C-circle abundance in cells described in Fig 2I. Values are presented relative to U2OS (set at 100). Bars: means and SDs of 3 experiments. All *p* values were derived from a one-way ANOVA with Tukey correction. Symbols as in Fig 1. **(B)** CO-FISH staining on metaphase spreads from the indicated cell lines, as in Fig 1I. Chromosome ends displaying telomere exchanges are indicated with an x, ECTSs are marked by an arrow, and sister associations are denoted by an asterisk. **(C)** Quantification of telomere exchanges detected by CO-FISH. Each data point represents the percentage of chromosome ends with telomere exchanges in one metaphase spread. Bars: means and SDs. **(D)** FISH staining of cell lines described in Fig 2I with probes targeting the arm (red) and subtelomeric (green) regions of Chromosome 4. The underlying numerical data and statistical analysis for each figure panel can be found in S1 Data. ALT, alternative lengthening of telomeres; ATRX, alpha thalassemia/mental retardation syndrome X-linked chromatin remodeler; C-circle, extrachromosomal, circular telomeric DNA with an intact C-rich strand; CO-FISH, chromosome orientation fluorescence in situ hybridization; ECTS, extrachromosomal telomeric signal; FISH, fluorescence in situ hybridization; SD, standard deviation; U2OS, human osteosarcoma cell line.
(TIF)

S4 Fig Generation of SA1 KO MEFs. **(A)** Schematic of the mouse SA1 locus, identifying features relevant to CRISPR/Cas9-mediated gene editing. **(B)** DNA sequences of the edited SA1 alleles in CRISPR/Cas9-derived KO clones obtained by TOPO (Thermo Fisher Scientific) cloning of PCR products using the primers shown in (A). Edits associated with each allele are specified. Bold text denotes the exon 10 sequence and regular text identifies the intron sequence. **(C-D)** Quantification of sister (C) and nonallelic (D) telomere associations in control and SA1 KO cells (no Cre) detected by CO-FISH (as in Fig 3). Data points represent the percentage of long arm chromosome ends displaying sister associations and the percentage of all chromatids associated with nonallelic telomeres in one metaphase spread. Bars: means and SDs of 19–20 metaphases from 2 experiments. All *p*-values were derived from a one-way ANOVA with Tukey correction. Symbols as in Fig 1. The underlying numerical data and statistical analysis for each figure panel can be found in S1 Data. Cas9, CRISPR associated protein 9; CO-FISH, chromosome orientation fluorescence in situ hybridization; Cre, recombinase acting on Lox sites; CRISPR, clustered regularly interspaced short palindromic repeats; KO, knockout; MEF, mouse embryonic fibroblast; ns, not significant; PCR, polymerase chain reaction; SA1, stromal antigen 1; SD, standard deviation.
(TIF)

S5 Fig Characterization of ALT-like phenotypes in SA1 KO MEFs. **(A-C)** Quantification of the number of TIFs per cell (A), telomere-PML co-localizations (APBs) per cell (B), or PML foci per cell (C) in MEFs expressing FokI^WT^-TRF1, as assayed in Fig 4. Bars: means and SDs of >300 cells. All *p*-values were derived from a one-way ANOVA with Tukey correction. Symbols as in Fig 1. The underlying numerical data and statistical analysis for each figure panel can be found in S1 Data. ALT, alternative lengthening of telomeres; APB, ALT-associated PML body; FokI^WT^-TRF1, wild-type FokI nuclease domain and telomeric repeat binding factor 1 fusion protein; KO, knockout; ns, not significant; MEF, mouse embryonic fibroblast; PML, promyelocytic leukemia; SA1, stromal antigen; SD, standard deviation; TIF, telomere dysfunction–induced foci.
(TIF)

S6 Fig Generation and characterization of DAXX KO MEFs. **(A)** Schematic of the mouse DAXX locus, identifying features relevant to CRISPR/Cas9-mediated gene editing. **(B)** DNA sequences of the edited DAXX alleles in 2 CRISPR/Cas9-derived ATRX^F/y^ KO clones obtained by TOPO (Thermo Fisher Scientific) cloning of PCR products using the primers shown in (A). Deletions associated with each allele are specified. **(C-E)** Quantification of the number of TIFs per cell (C), telomere-PML co-localizations (APBs) per cell (D), or PML foci per cell (E) in FokI^WT^-TRF1–expressing cells, as assayed in Fig 5. Bars: means and SDs of approximately 300 cells for DAXX KO MEFs and approximately 200 cells for control MEFs. **(F)** Quantification of the number of PML foci per cell in control and a DAXX-targeted population of cells expressing FokI-ER^T2^-TRF1, after Cre-mediated deletion of ATRX or shRNA depletion of SA1/SA2 (as assayed in Fig 6D). Bars: means and SDs of >330 cells. Pairwise comparisons in panels C, D, and E were derived from a two-tailed, unpaired *t* test. All other *p*-values were derived from a one-way ANOVA with Tukey correction. Symbols as in Fig 1. The underlying numerical data and statistical analysis for each figure panel can be found in S1 Data. APB, ALT-associated PML body; ATRX, alpha thalassemia/mental retardation syndrome X-linked chromatin remodeler; ATRX^F/y^, male embryo with a single floxed allele; Cas9, CRISPR associated protein 9; Cre, recombinase acting on Lox sites; CRISPR, clustered regularly interspaced short palindromic repeats; DAXX, death domain-associated protein; FokI-ER^T2^-TRF1, tamoxifen-inducible FokI-TRF1 fusion protein; FokI^WT^-TRF1, wild-type FokI nuclease domain and telomeric repeat binding factor 1 fusion protein; KO, knockout; MEF, mouse embryonic fibroblast; ns, not significant; PCR, polymerase chain reaction; PML, promyelocytic leukemia; SA1, stromal antigen 1; SA2, stromal antigen 2; SD, standard deviation; shRNA, short hairpin RNA; TIF, telomere dysfunction–induced foci.
(TIF)

S7 Fig DAXX deletion does not induce telomere cohesion defects. **(A)** FISH of the arm (red) and subtelomeric (green) probes on Chromosome 10 in interphase cells from DAXX KO MEFs described in Fig 5 and S6 Fig. **(B)** Quantification of the Chromosome 10 FISH signals observed as doublets. Bars: means and SDs of 3 experiments. **(C)** Immunoblots showing phosphorylation of MCM2 S108 in TPP1-deficient cells, representative of DNA damage signaling and efficient deletion of TPP1. Cre-mediated deletion of ATRX and CRISPR/Cas9-targeting of SA1 and DAXX are also shown. γtubulin serves as a loading control. **(D)** Representative images of sister and nonallelic telomere associations detected by CO-FISH staining (as in Fig 1G) on metaphase spreads. Telomere associations are marked by asterisks (*, sister association; **, nonallelic association). **(E-F)** Quantification of sister (E) and nonallelic (F) telomere associations detected by CO-FISH as in (D). Data points represent the percentage of long arm chromosome ends displaying sister associations and the percentage of all chromatids associated with nonallelic telomeres in one metaphase spread. Bars: means and SDs from 3 experiments. Pairwise comparisons in panel B were derived from a two-tailed, unpaired *t* test. All other *p*-values were derived from a one-way ANOVA with Tukey correction. Symbols as in Fig 1. The underlying numerical data and statistical analysis for each figure panel can be found in S1 Data. ATRX, alpha thalassemia/mental retardation syndrome X-linked chromatin remodeler; Cas9, CRISPR associated protein 9; CO-FISH, chromosome orientation fluorescence in situ hybridization; Cre, recombinase acting on Lox sites; CRISPR, clustered regularly interspaced short palindromic repeats; DAXX, death domain-associated protein; FISH, fluorescence in situ hybridization; KO, knockout; MCM2, minichromosome maintenance complex component 2; MEF, mouse embryonic fibroblast; ns, not significant; SA1, stromal antigen 1; SD, standard deviation; TPP1, ACD shelterin complex subunit and telomerase recruitment factor.
(TIF)

## Abbreviations

ALT: alternative lengthening of telomeres
APB: ALT-associated PML body
ARID1A: AT-rich interaction domain 1A
ATRX: alpha thalassemia/mental retardation syndrome X-linked chromatin remodeler
ATRX^F/F^: female embryo with two floxed ATRX alleles
ATRX^F/y^: male embryo with a single floxed ATRX allele
BIR: break-induced replication
C-circle: extrachromosomal, circular telomeric DNA with an intact C-rich strand
Cas9: CRISPR associated protein 9
cGAS: cyclic GMP-AMP synthase
ChIP: chromatin immunoprecipitation
CO-FISH: chromosome orientation fluorescence in situ hybridization
Cre: recombinase acting on Lox sites
CRISPR: clustered regularly interspaced short palindromic repeats
DAXX: death domain-associated protein
DKO: double knockout
DSB: double-strand break
ECTS: extrachromosomal telomeric signal
FACS: fluorescence-activated cell sorting
FISH: fluorescence in situ hybridization
FokI-TRF1: FokI nuclease domain and telomeric repeat binding factor 1 fusion protein
FokI^DA-^TRF1: nuclease dead FokI-TRF1 fusion protein
FokI-ER^T2^-TRF1: tamoxifen-inducible nuclear localized FokI-TRF1 fusion protein
FokI^WT^-TRF1: wild-type FokI-TRF1 fusion protein
G-circle: extrachromosomal, circular telomeric DNA with an intact G-rich strand
gDNA: genomic DNA
HDR: homology-directed repair
hTERT: human telomerase reverse transcriptase
IF: immunofluorescence
KO: knockout
LTGC: long tract gene conversion
MCM2: minichromosome maintenance complex component 2
MEF: mouse embryonic fibroblast
PanNET: pancreatic neuroendocrine tumor
PCR: polymerase chain reaction
PD: population doubling
PFGE: pulsed-field gel electrophoresis
PI: pre-immune serum
PML: promyelocytic leukemia
PNA-FISH: peptide nucleic acid fluorescence in situ hybridization
POT1: protection of telomeres 1
pWZL: retroviral vector
RAD21: Rad21 cohesin complex component
SA1: stromal antigen 1
SA2: stromal antigen 2
sgDAXX: single guide RNA for DAXX
sgSA1: single guide RNA for SA1
shRNA: short hairpin RNA
SMC1: structural maintenance of chromosomes 1
SMC3: structural maintenance of chromosomes 3
SNF2: sucrose nonfermenting
STING: stimulator of interferon genes
SV40: simian virus 40
T-SCE: telomere sister chromatid exchange
TIF: telomere dysfunction–induced foci
TPP1: ACD shelterin complex subunit and telomerase recruitment factor
TRF1: telomeric repeat binding factor 1
U2OS: human osteosarcoma cell line
4-OHT: 4-hydroxytamoxifen
53BP1: tumor protein p53-binding protein 1
