# Supplementary material for: ATRX affects the repair of telomeric DSBs by promoting cohesion and a DAXX-dependent activity
Source: PLoS Biol. 2020 Jan 2;18(1):e3000594. doi: 10.1371/journal.pbio.3000594 (PMC6959610; doi:10.1371/journal.pbio.3000594)

Fig 1A
Scanned image of film

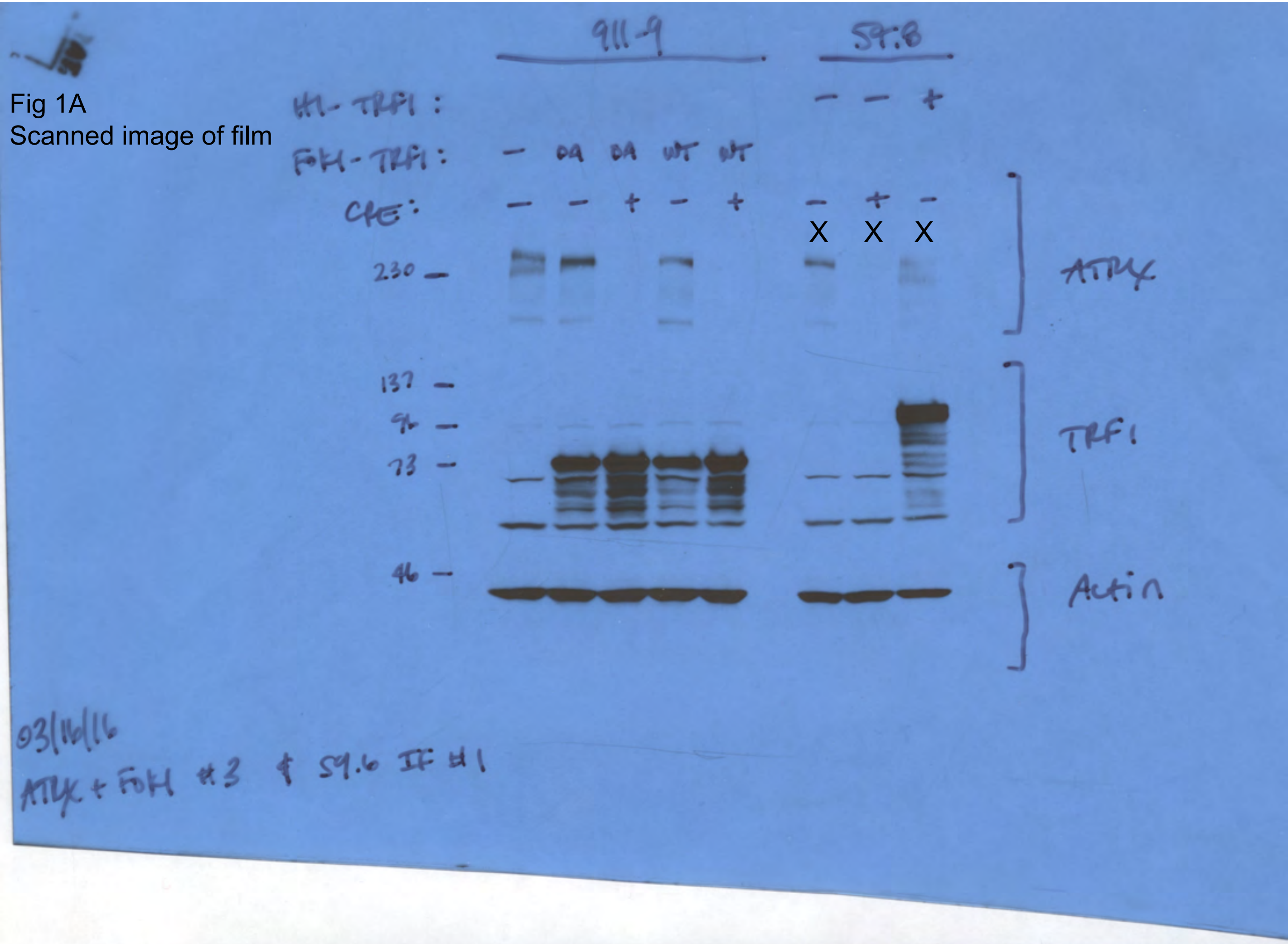

Fig 1A
Scanned image of film

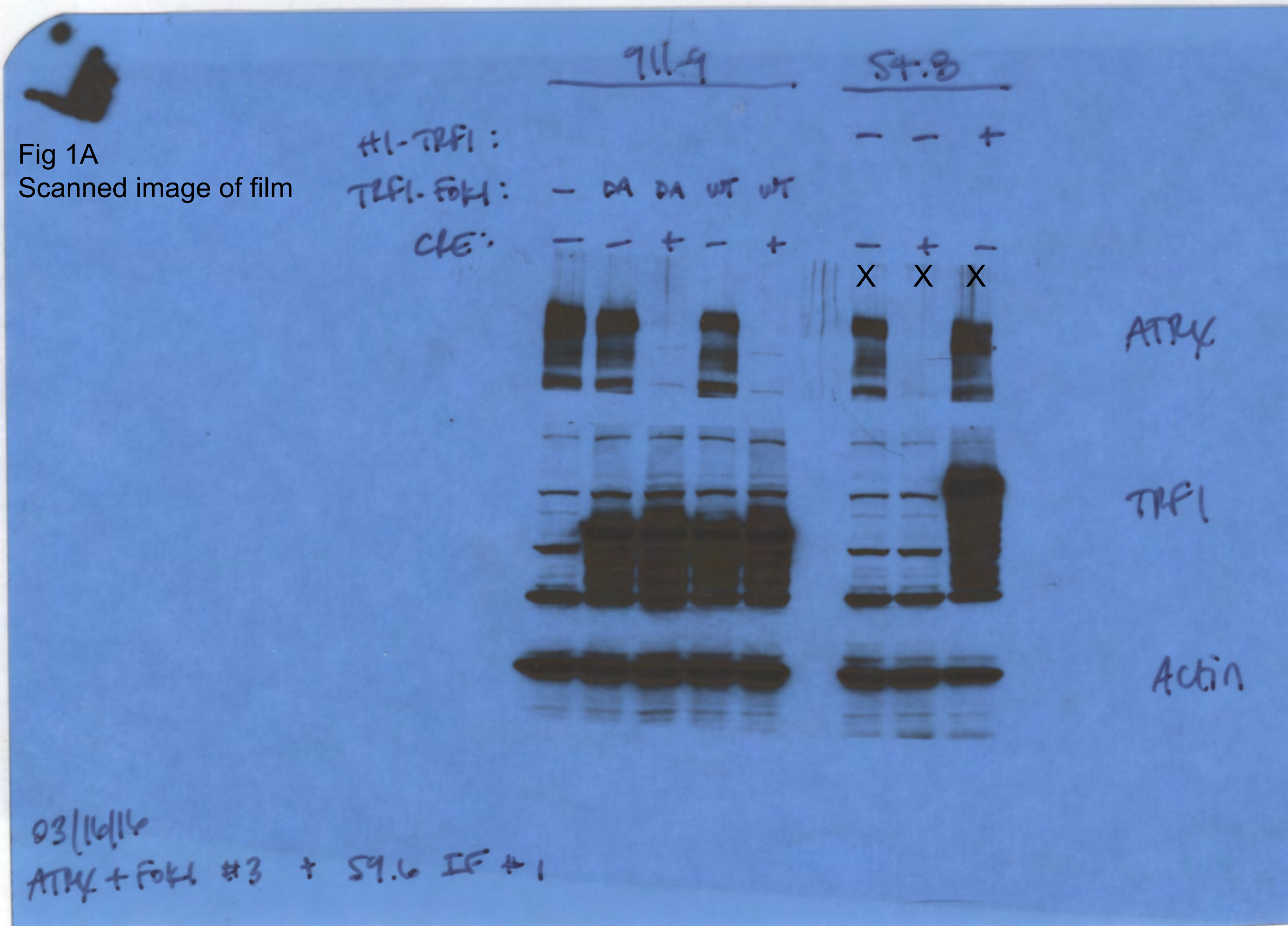

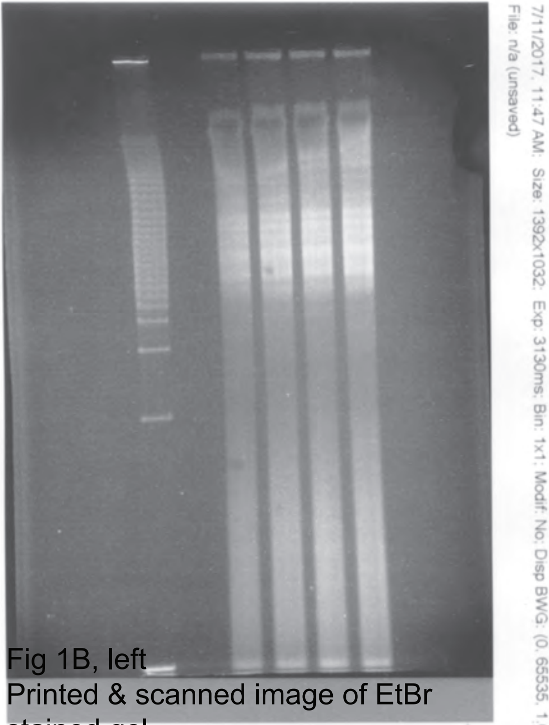

Fig 1B, left
Printed & scanned image of EtBr stained gel

Fig 1B, right
Phosphoimager scan
of native gel

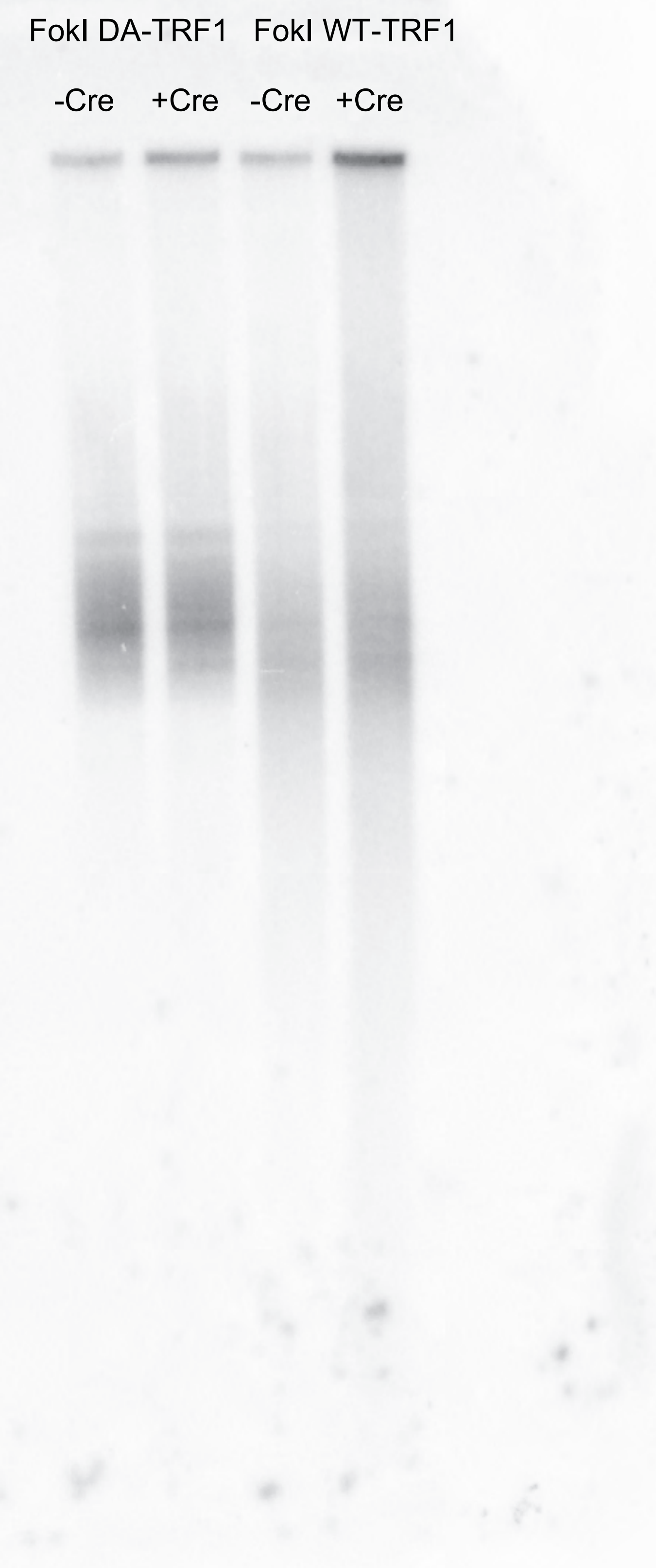

Fig 1J
Phosphoimager scan of native gel

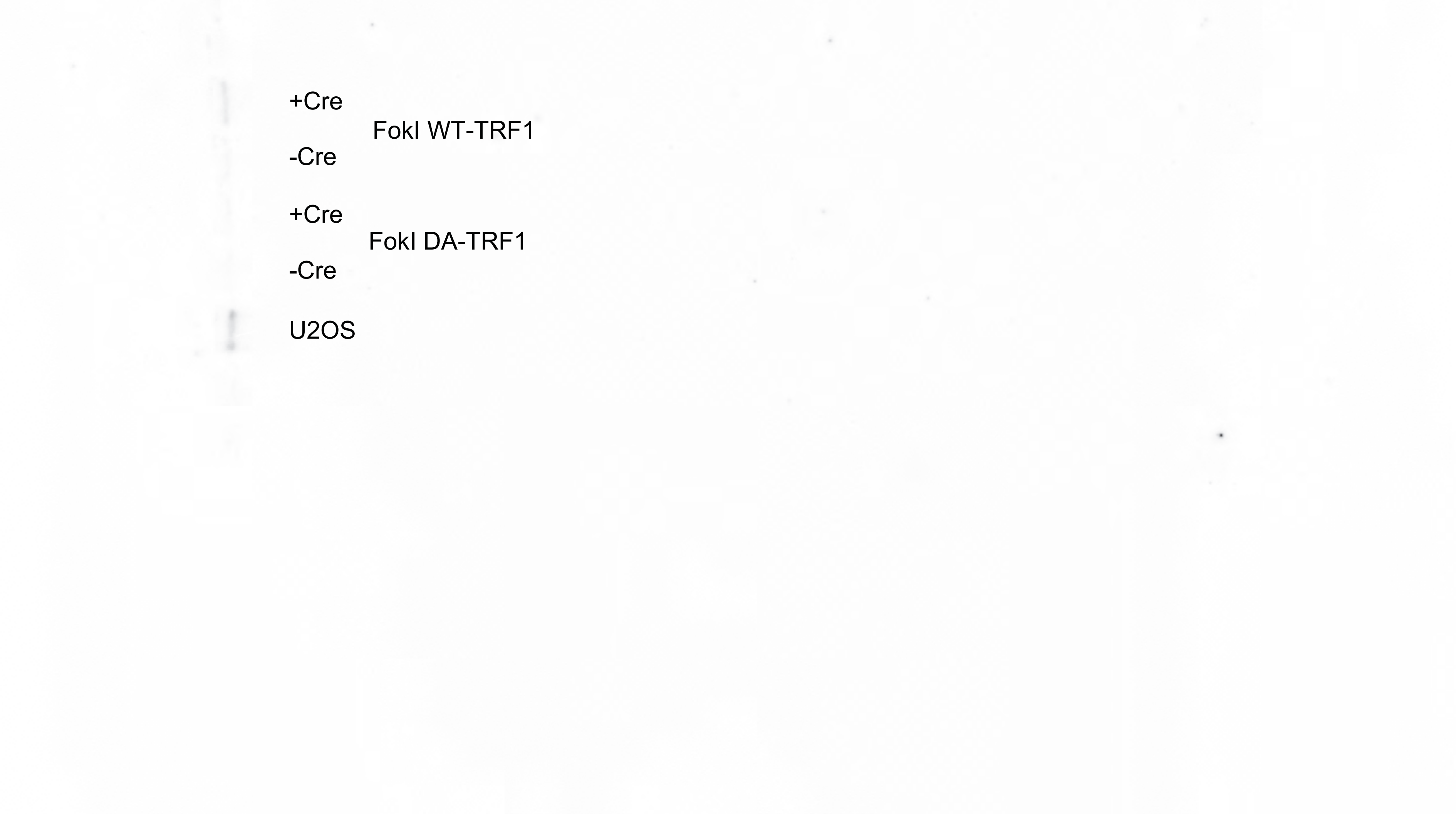

Fig 2A
Scanned image of film

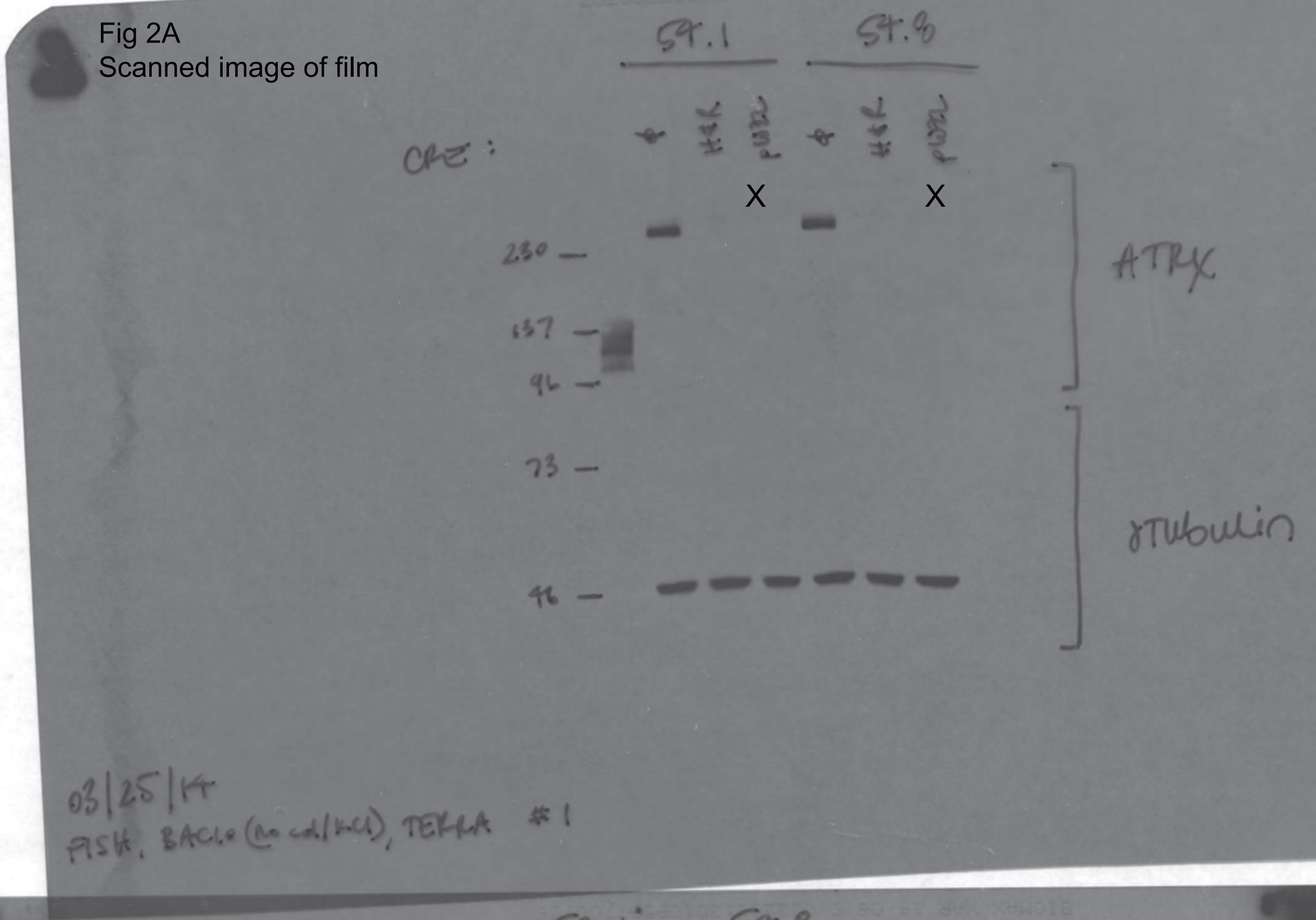

Fig 2A
Scanned image of film

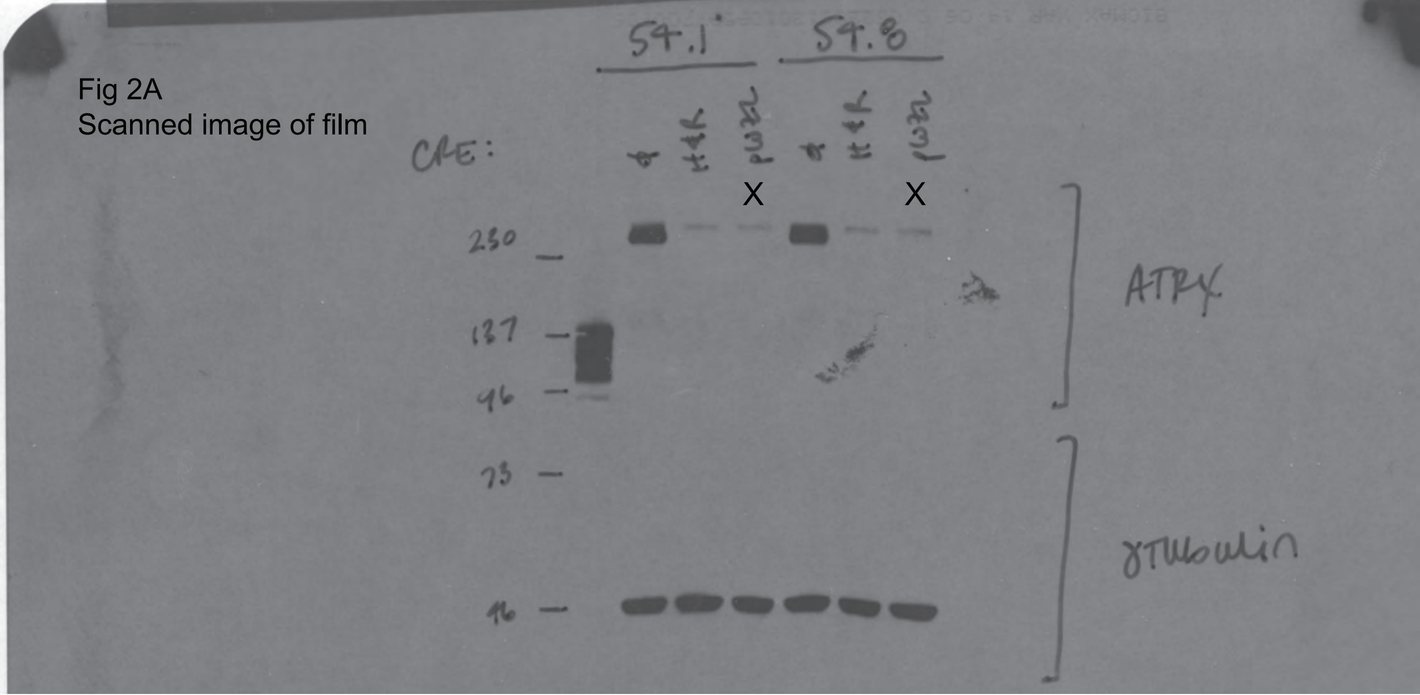

Fig 2D
Scanned image of film

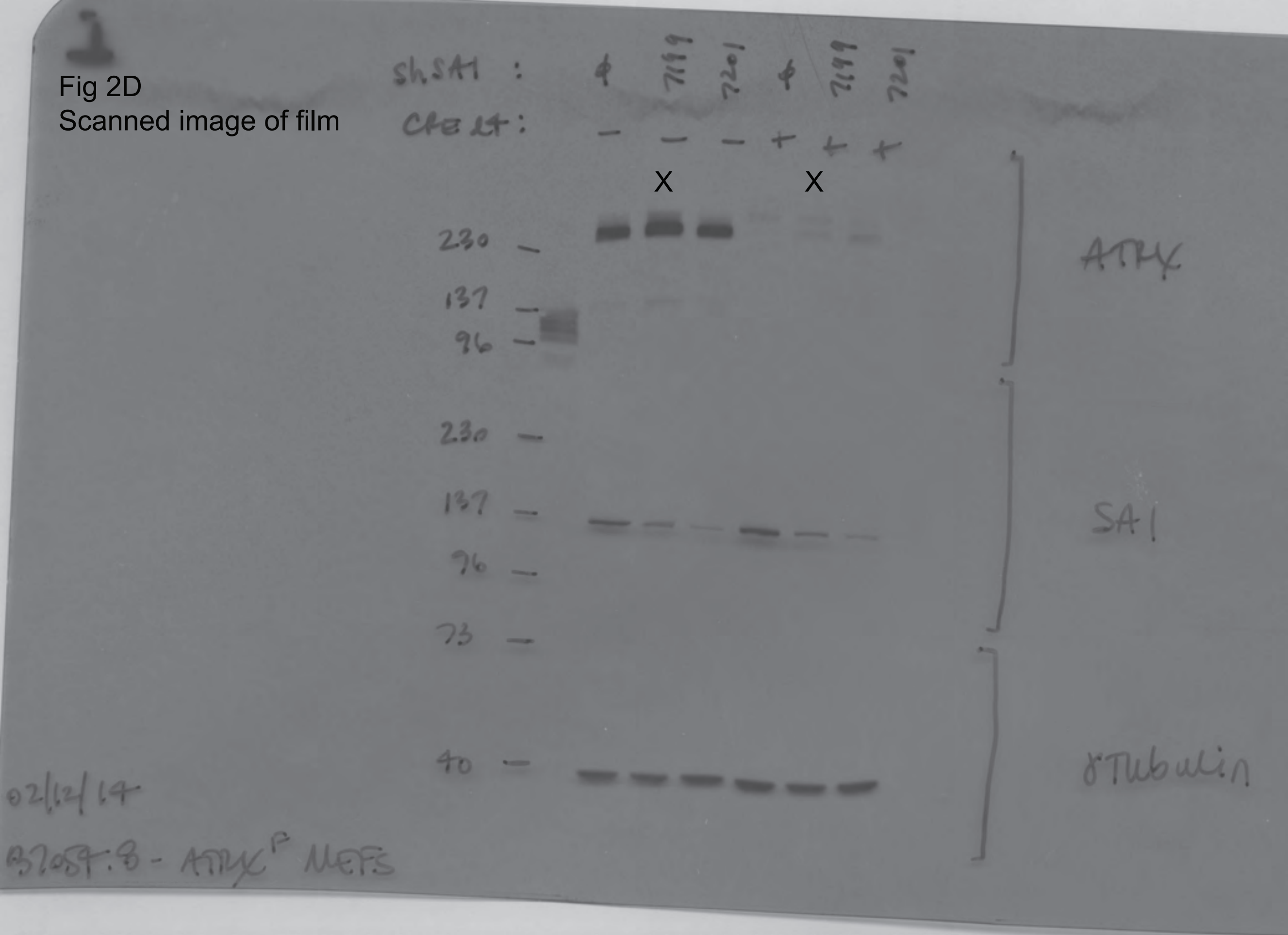

Fig 2D
Scanned image of film

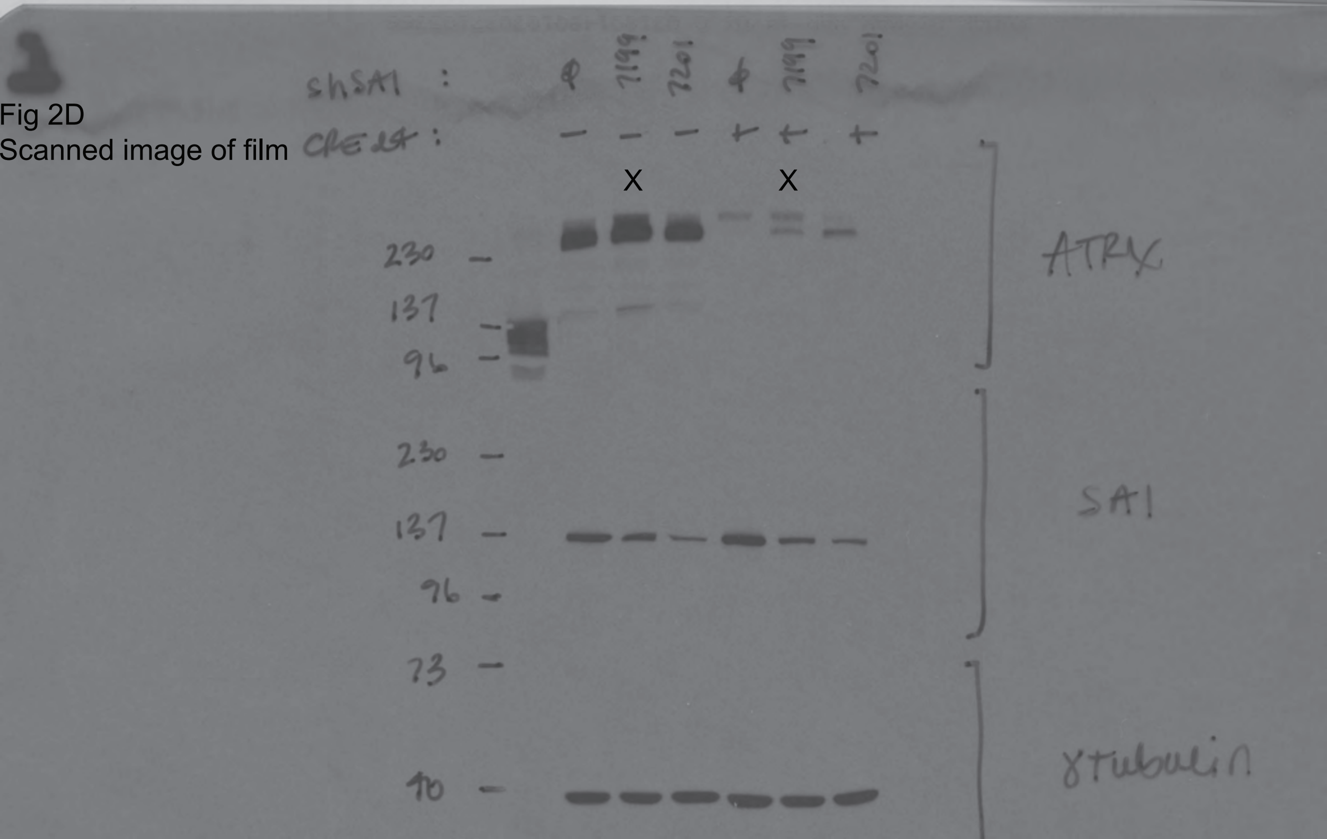

Fig 2I
Scanned image of film

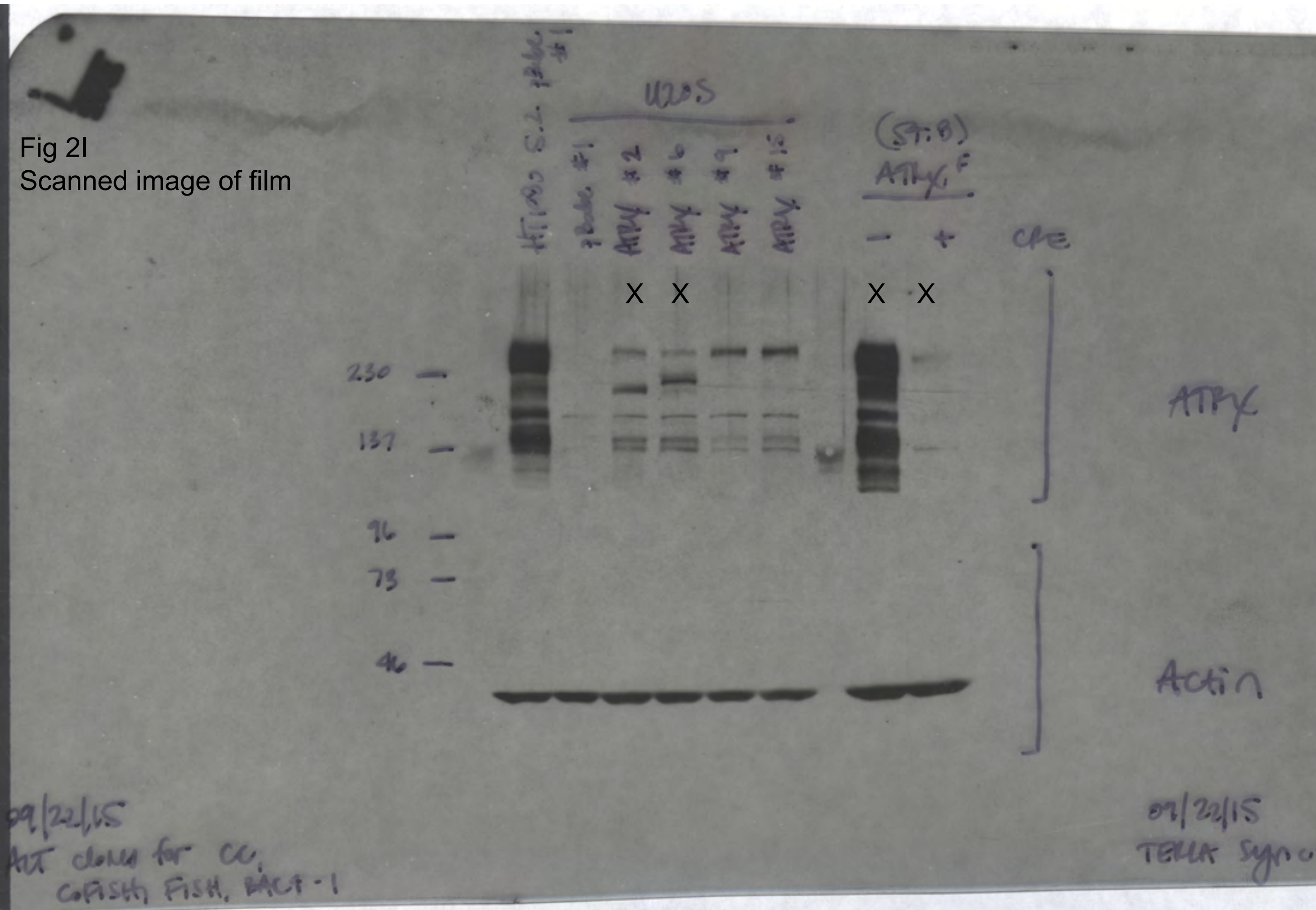

Fig 2I
Scanned image of film

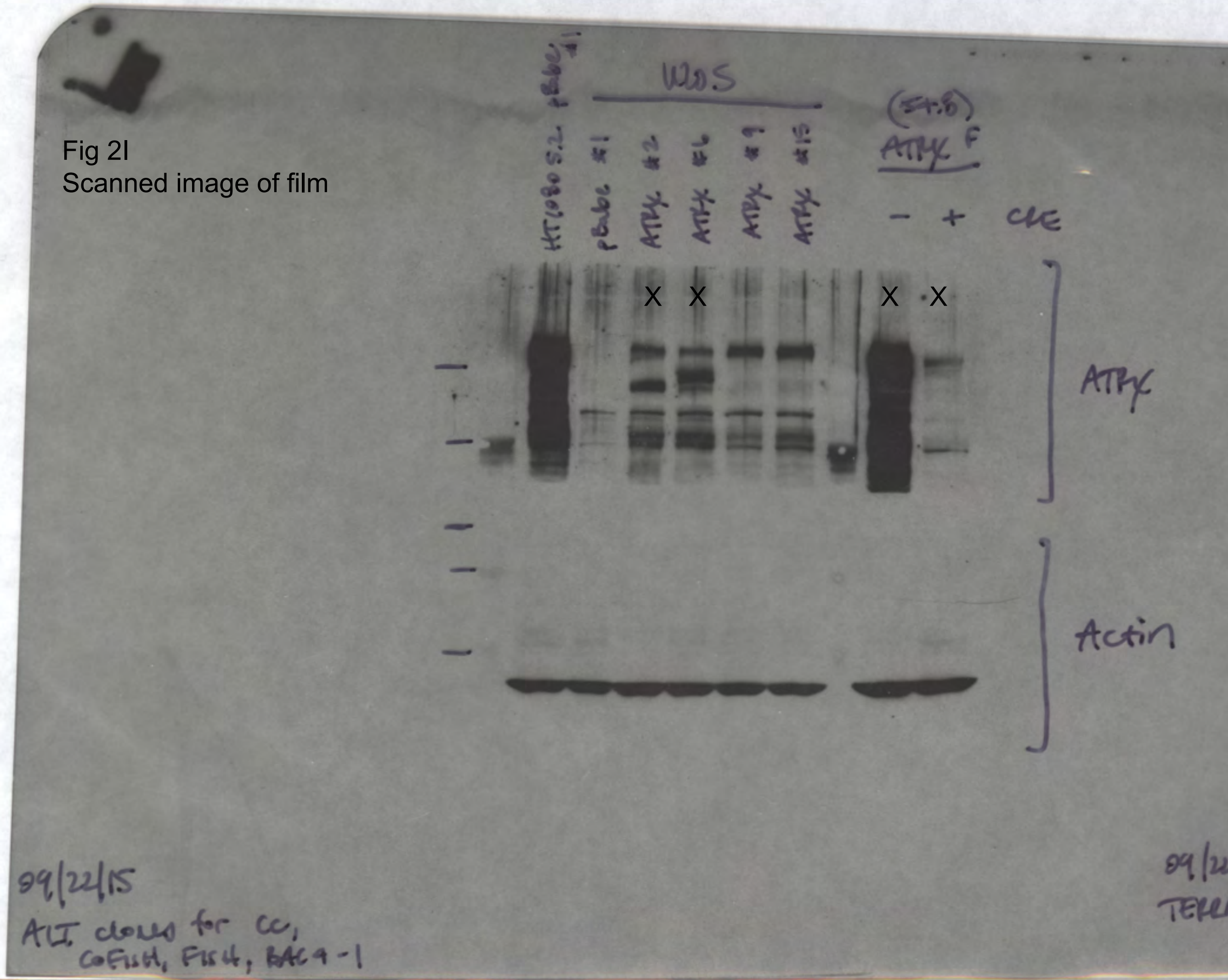

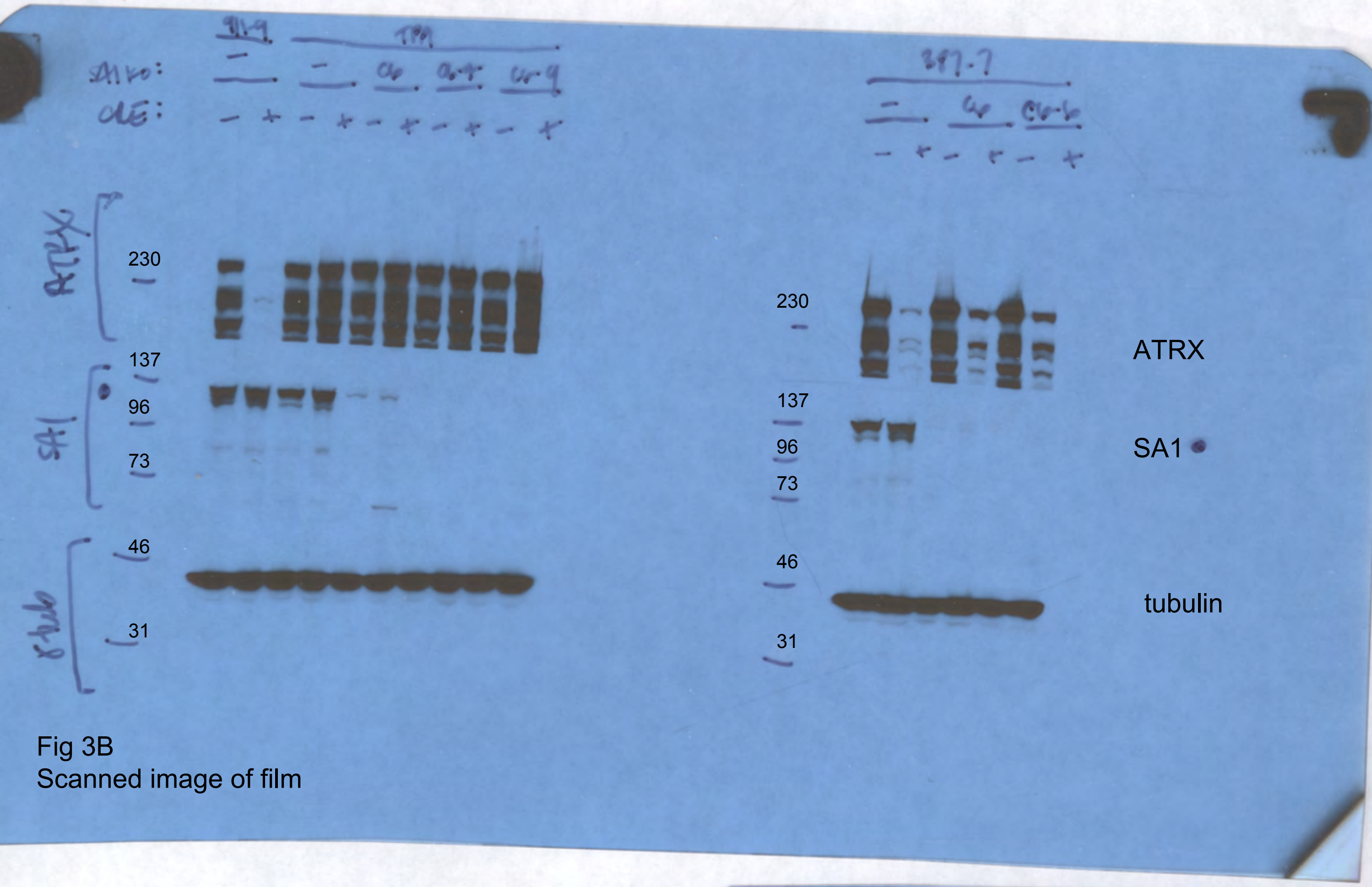

Fig 3B
Scanned image of film

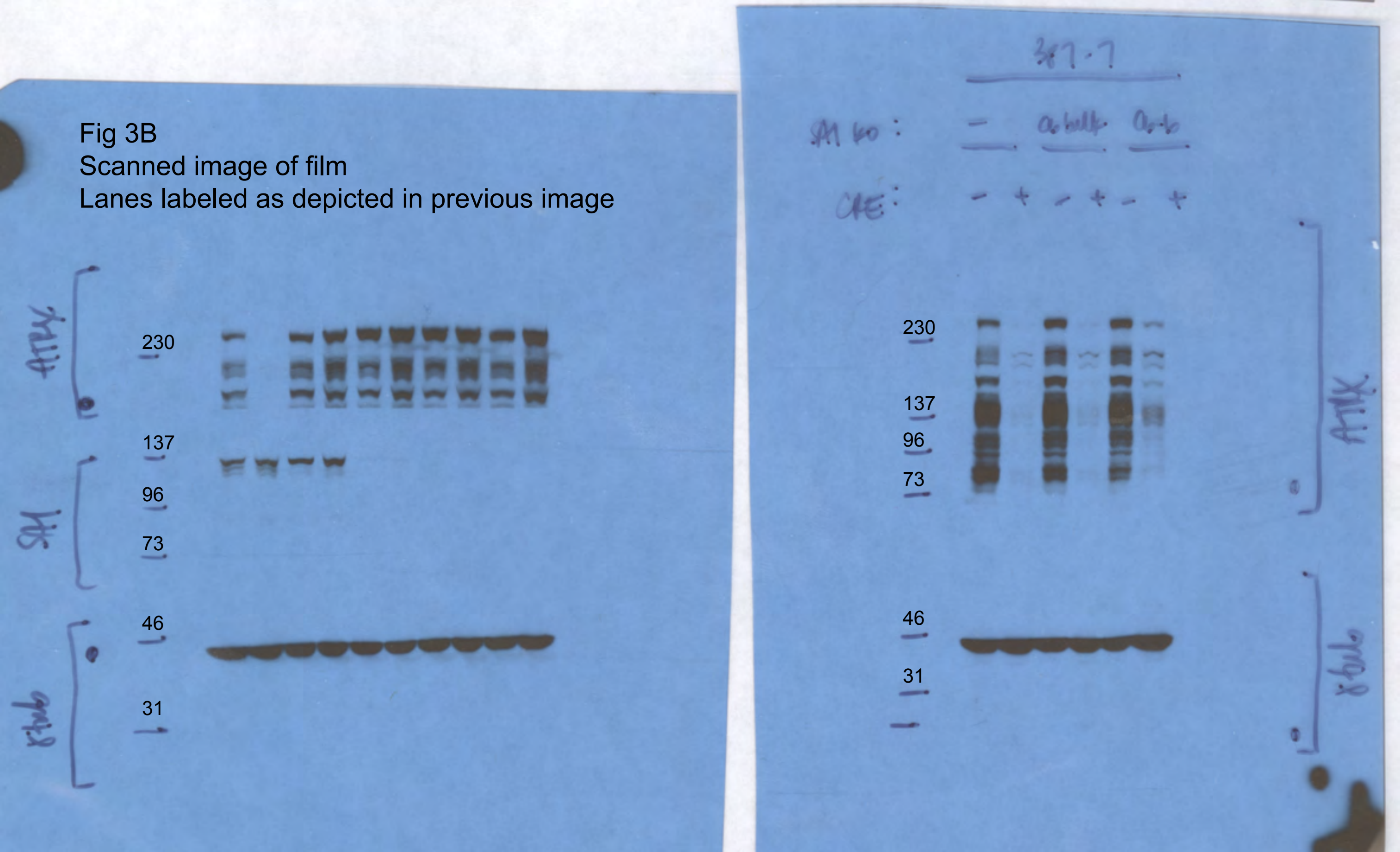

Fig 3B
Scanned image of film
Lanes labeled as depicted in previous image

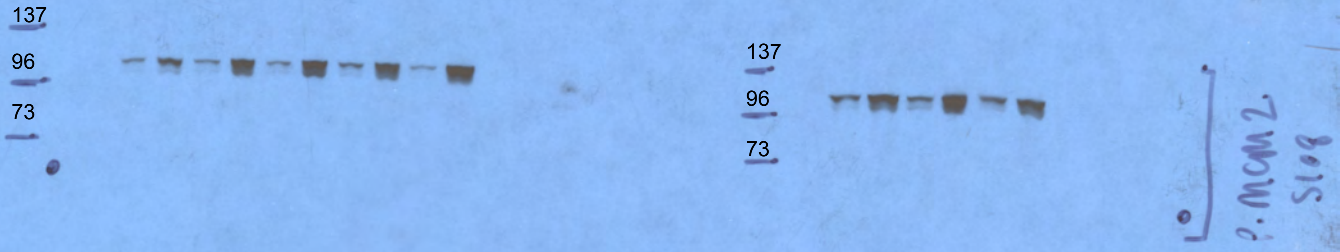

Fig 3B
Scanned image of film
Lanes labeled as depicted in first image of 3B

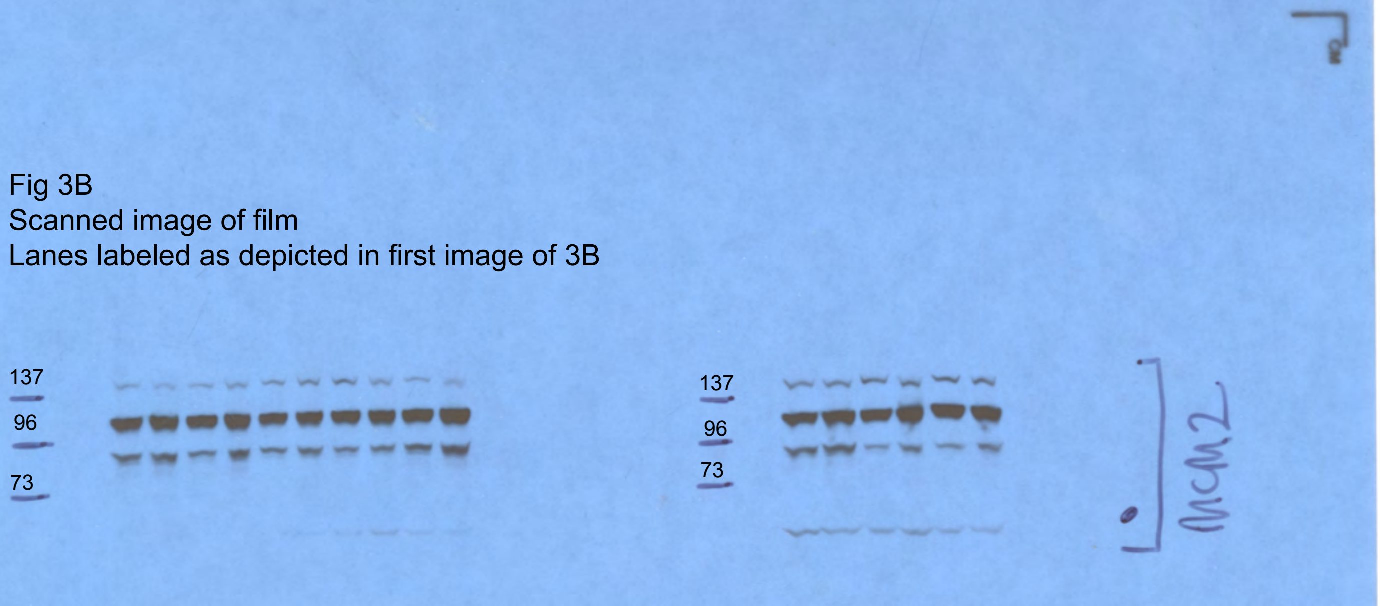

Fig 3B
Scanned image of film
Lanes labeled as depicted in first image of 3B

Fig 4A
Scanned image of film
Laned labeled as indicated below

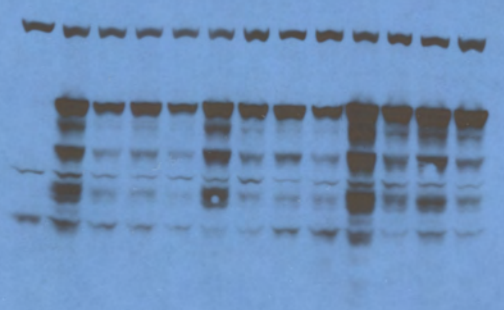

Fig 4A
Scanned image of film

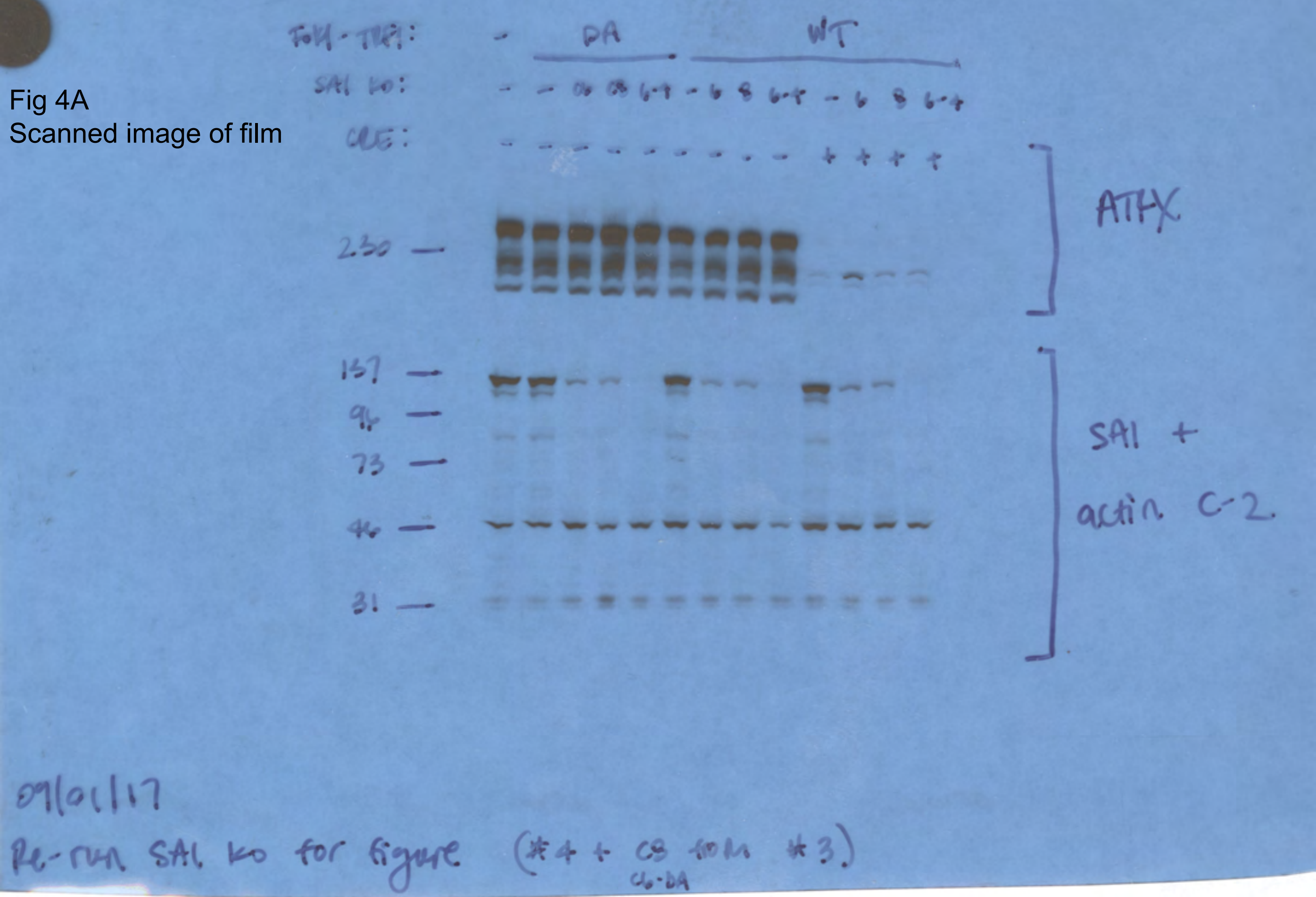

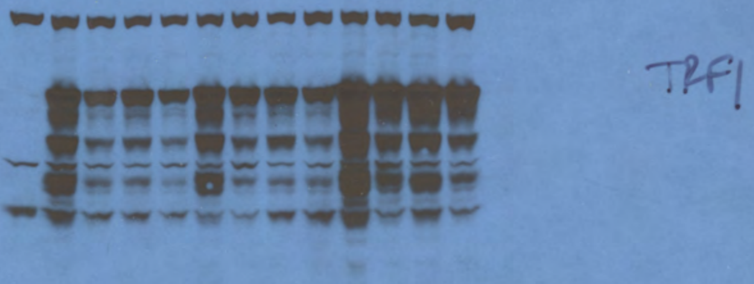

Fig 4A
Scanned image of film
Lanes labeled as indicated above

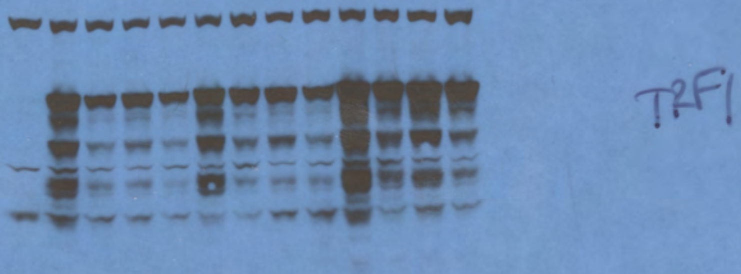

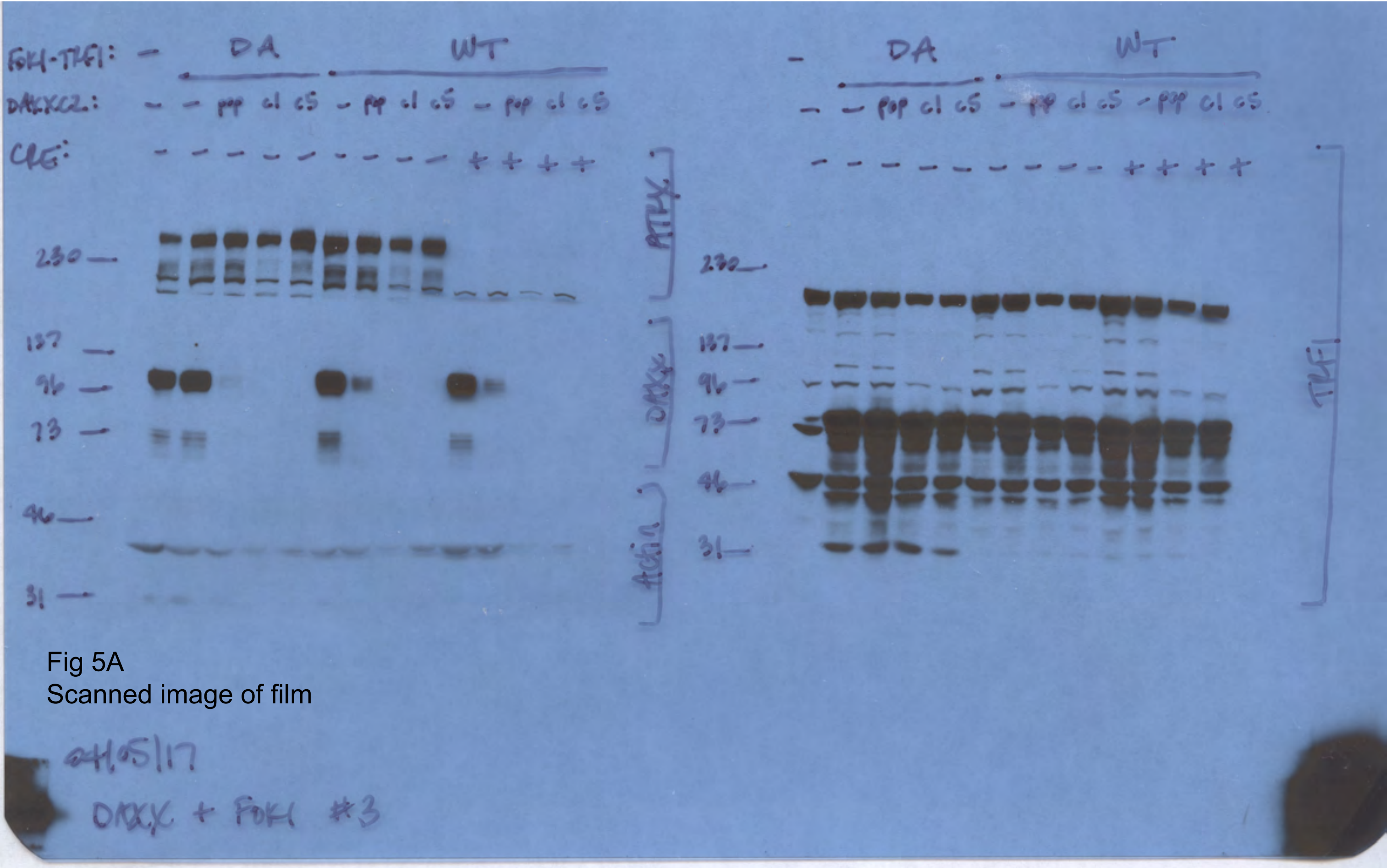

Fig 5A
Scanned image of film

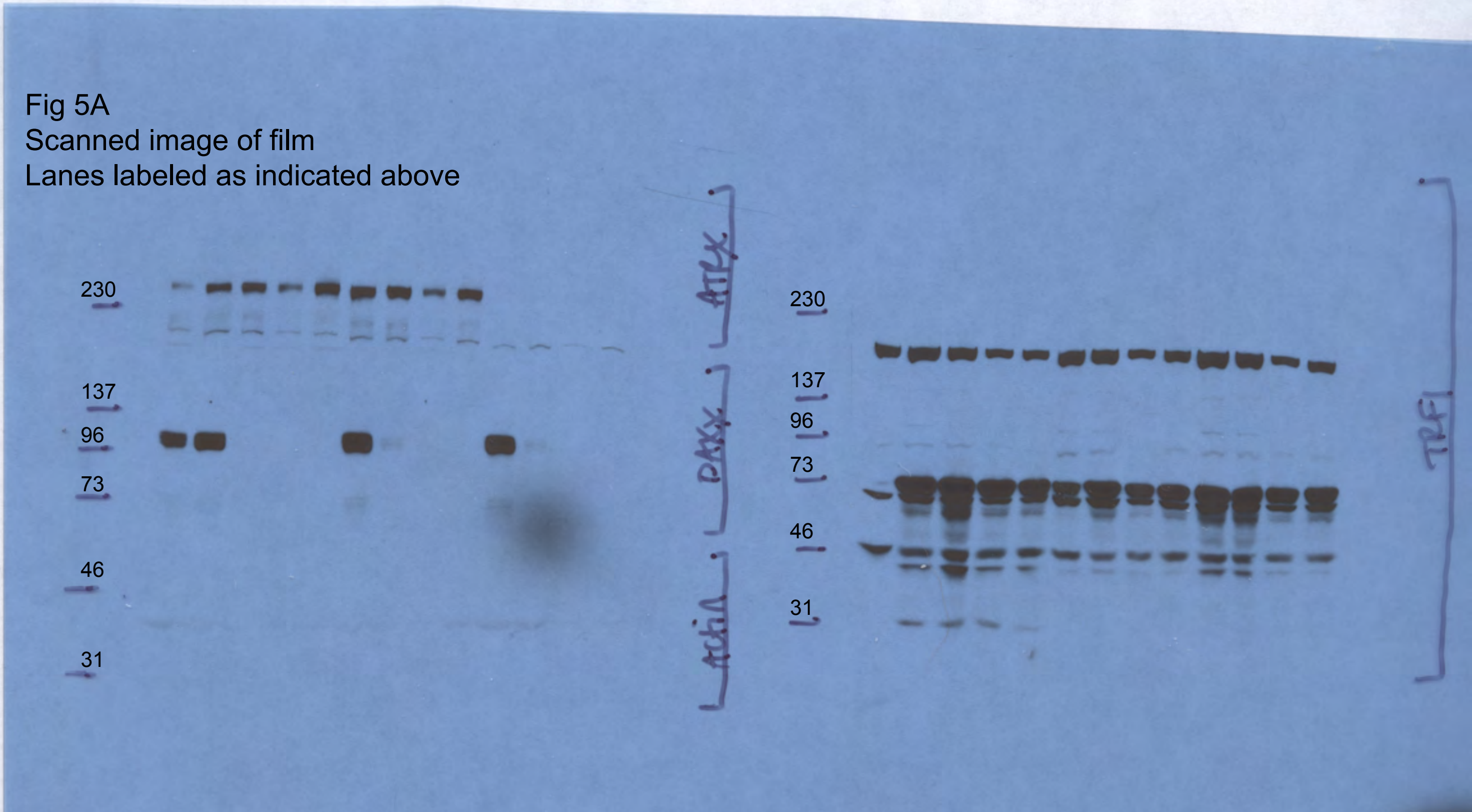

Fig 5A
Scanned image of film
Lanes labeled as indicated above

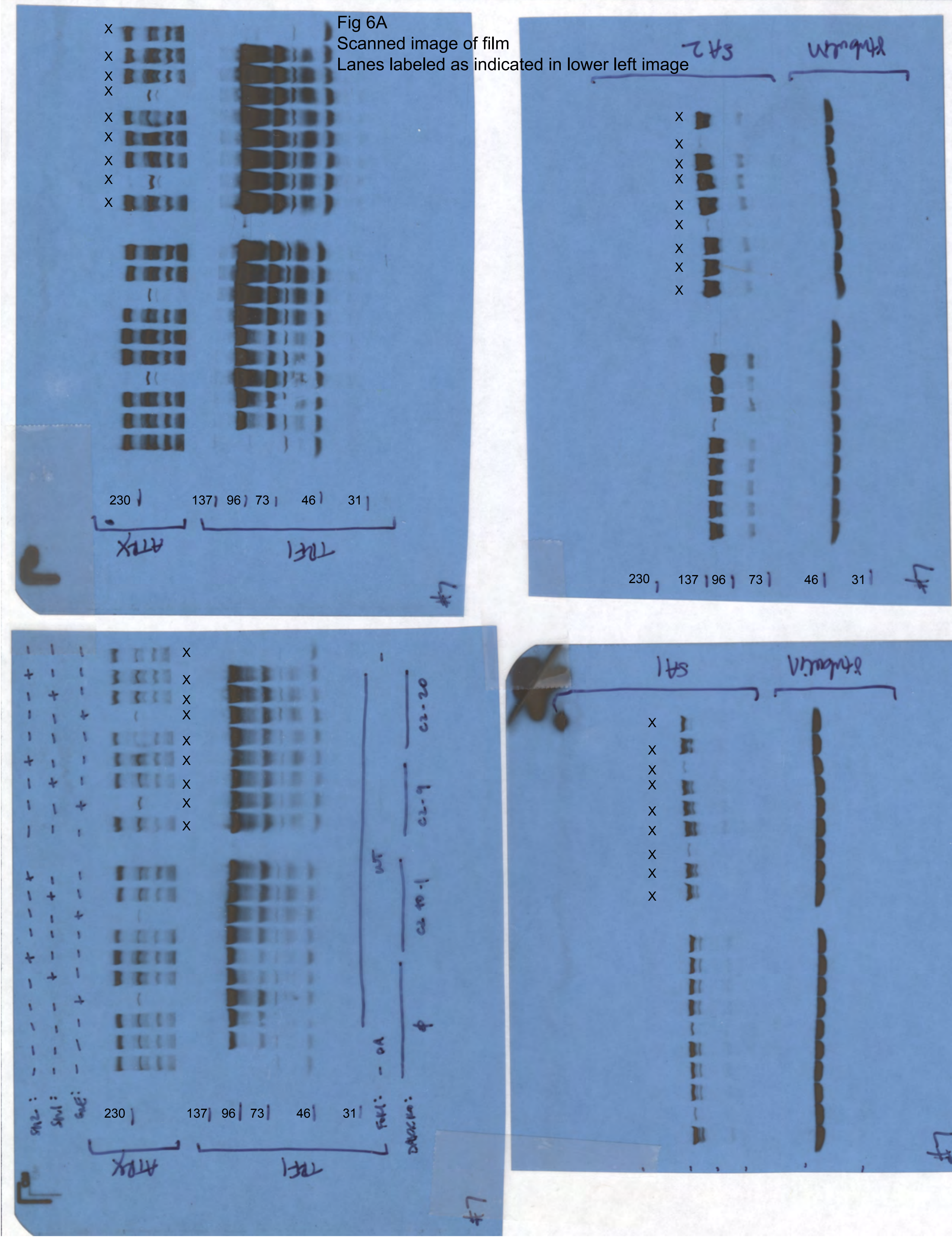

Fig 6A
Scanned image of film
Lanes labeled as indicated in lower left image

Fig 6A
Scanned image of film
Lanes labeled as shown above

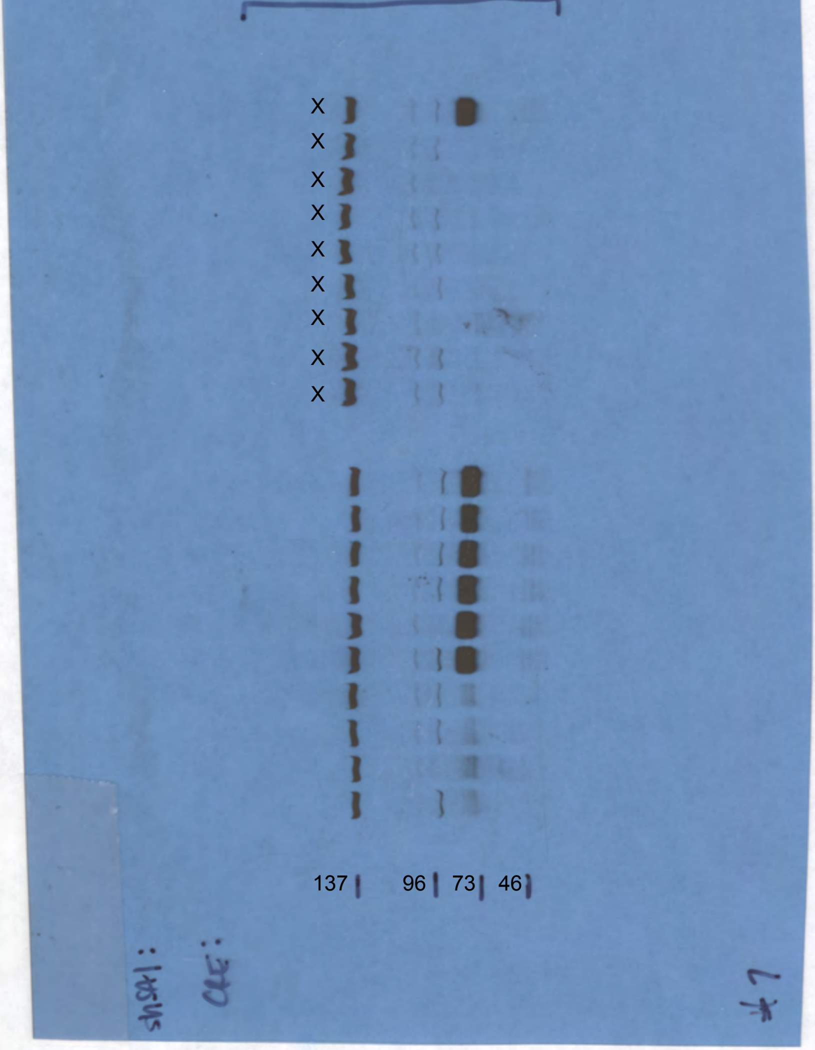

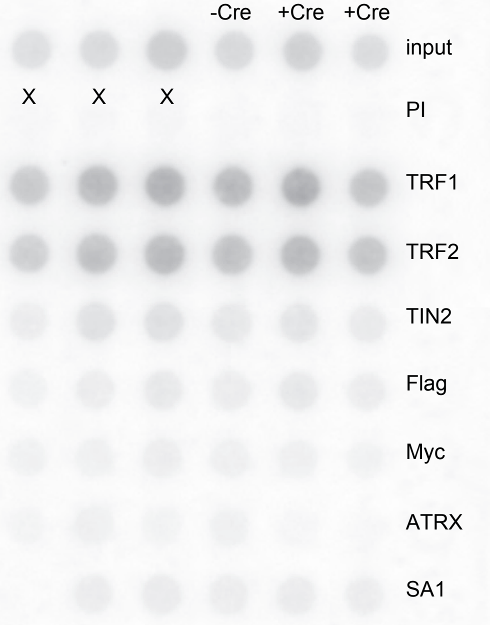

Fig S2D
Phosphoimager scan of telomeric ChIP blot

Fig S2E
Scanned image of film

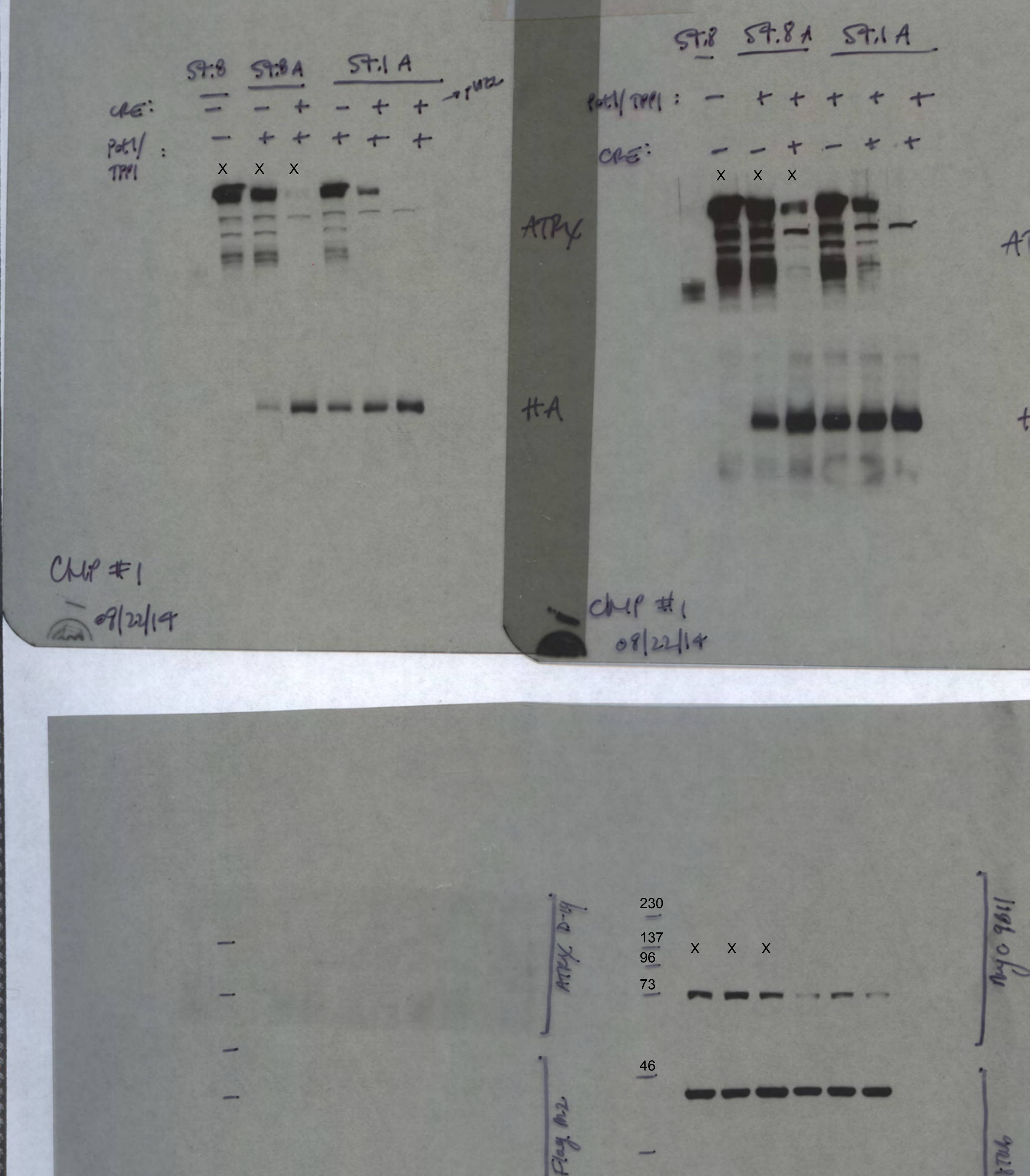

Fig S2H
Scanned image of film

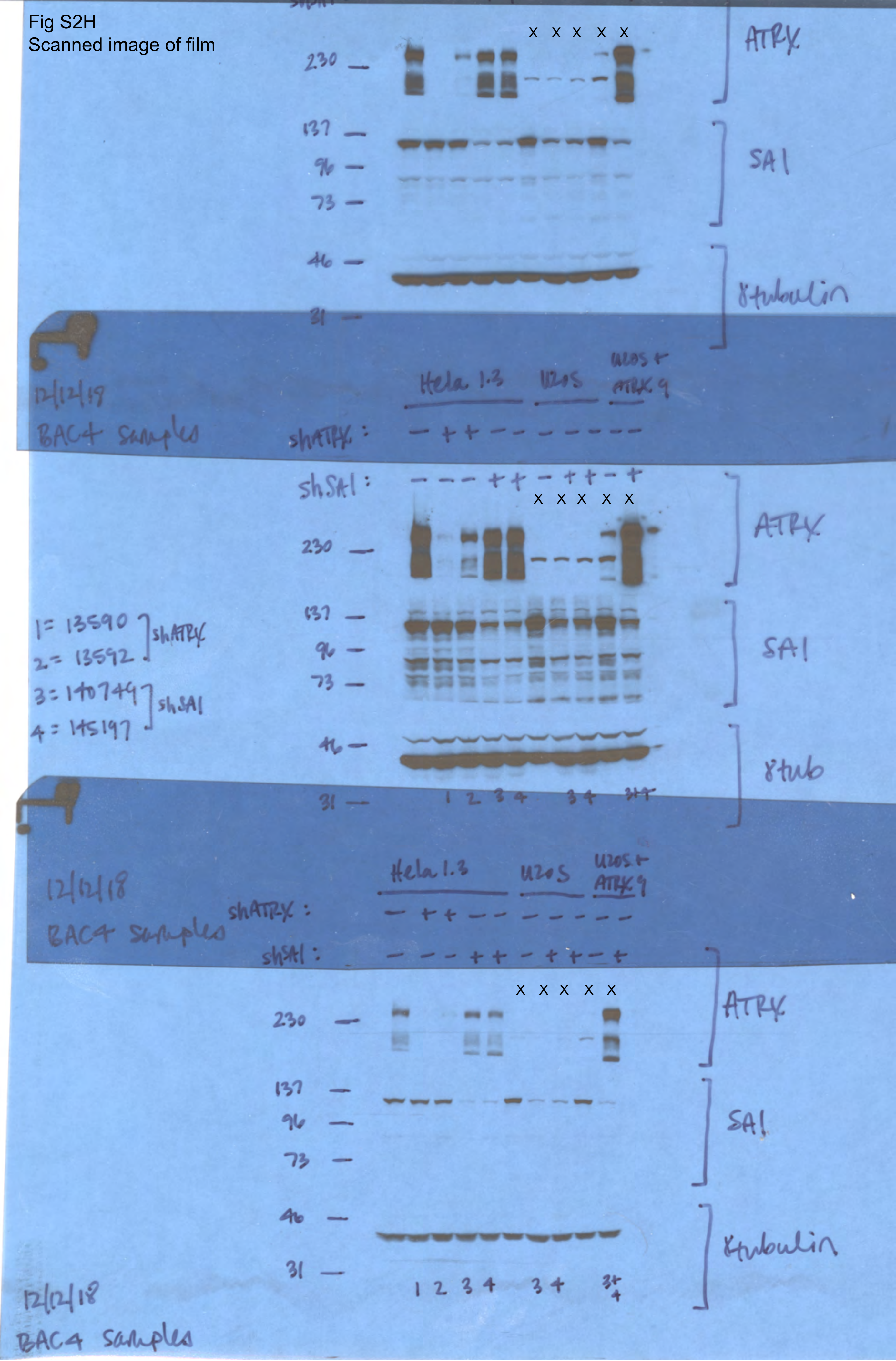

Fig S7C
Scanned image of film
Lanes labeled as indicated below

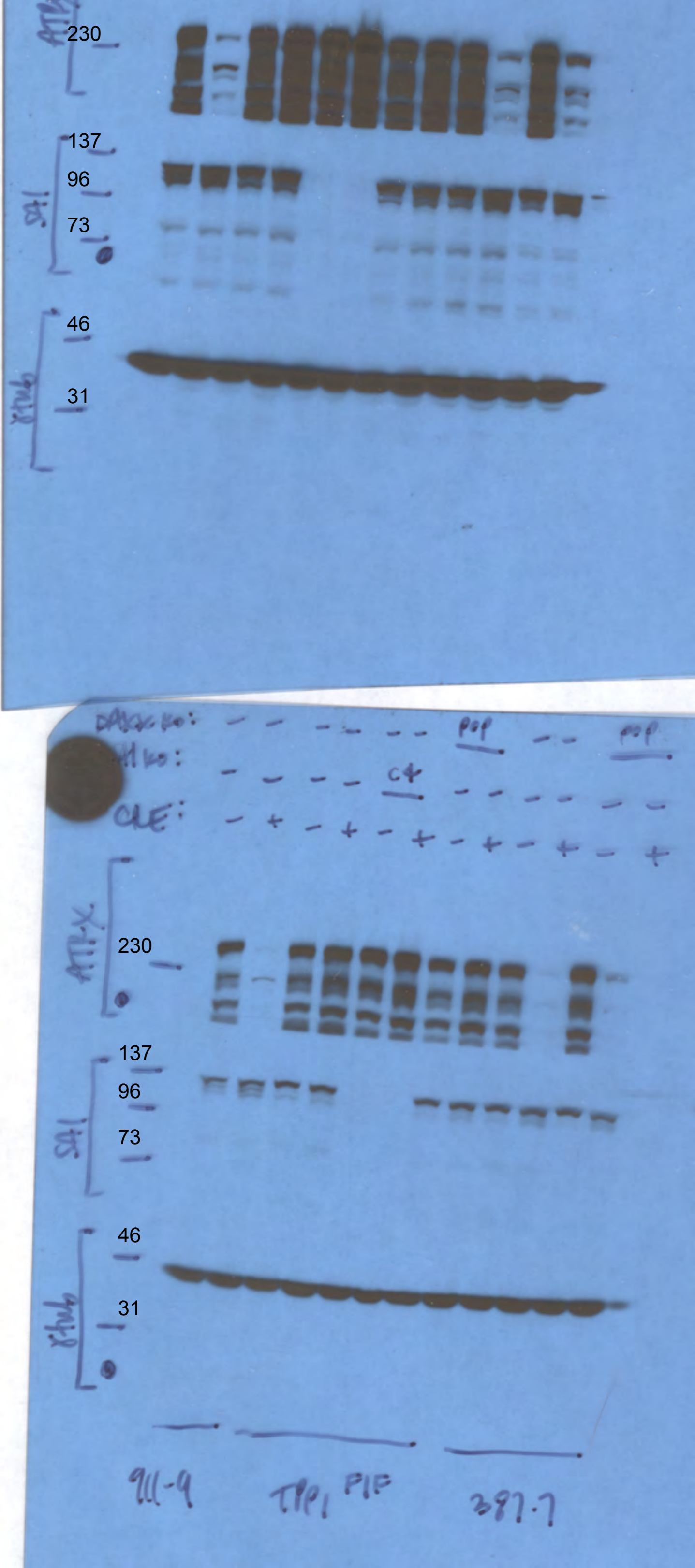

Fig S7C

Scanned image of film

Lanes labeled as indicated above

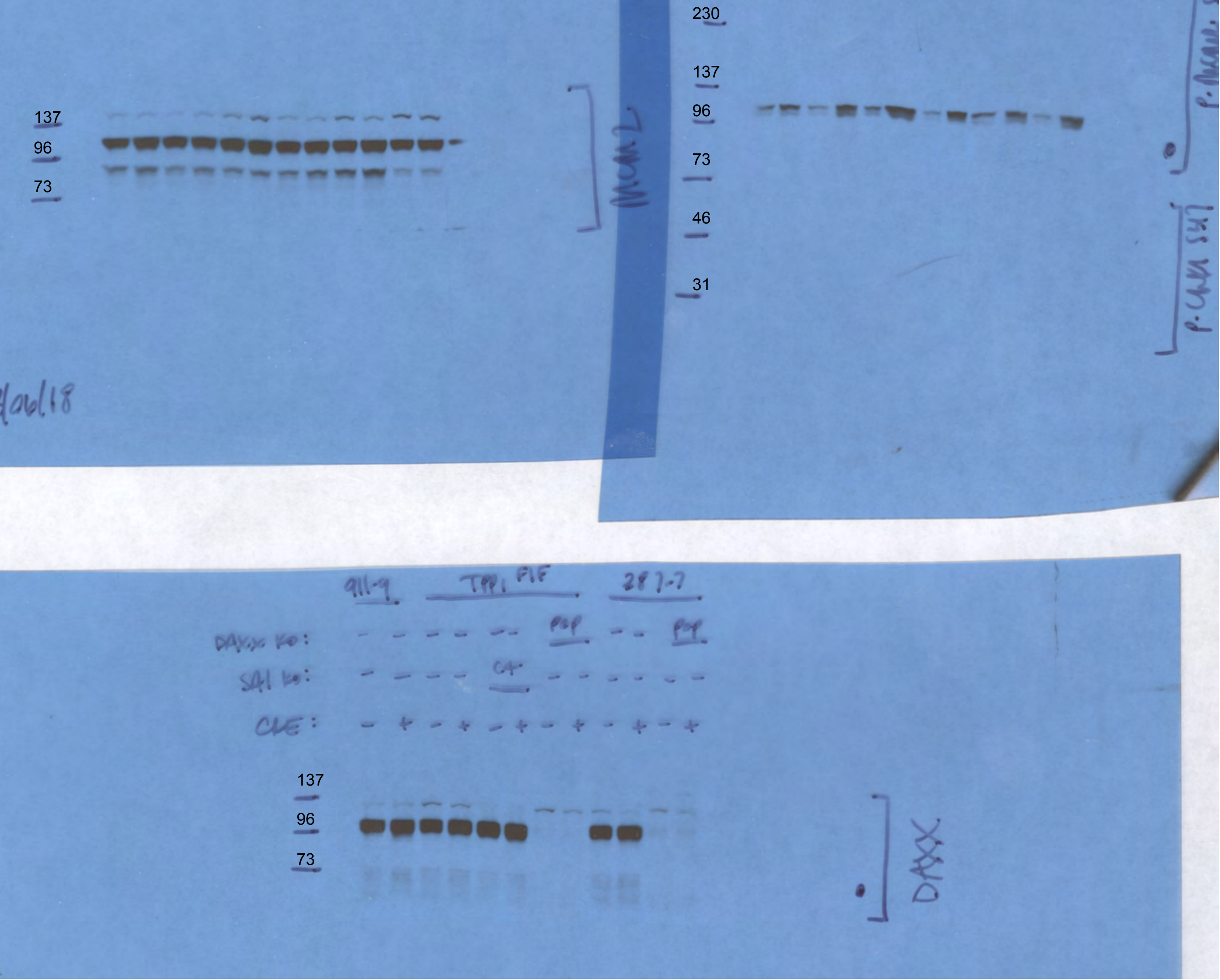

Supplement: S1 Raw Images — (PDF) [file pbio.3000594.s001.pdf]
