# Supplementary figures and images for: ATRX affects the repair of telomeric DSBs by promoting cohesion and a DAXX-dependent activity
Source: PLoS Biol. 2020 Jan 2;18(1):e3000594. doi: 10.1371/journal.pbio.3000594 (PMC6959610; doi:10.1371/journal.pbio.3000594)

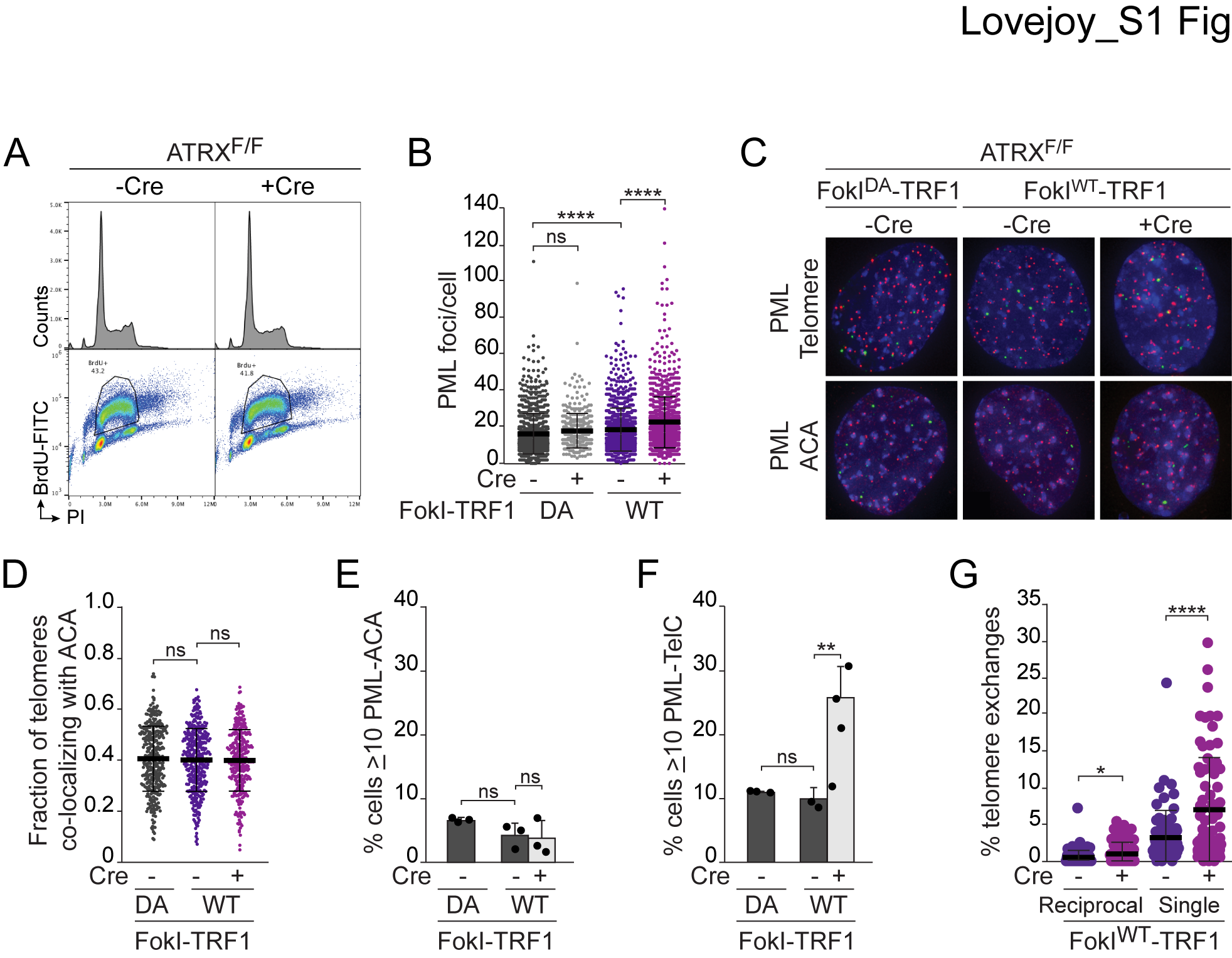

Supplement: S1 Fig — (A) FACS profiles of ATRXF/F MEFs treated with BrdU 4 days after Cre to monitor the S phase index. (B) Quantification of PML foci per cell in ATRXF/F MEFs, as assayed in Fig 1E. Bars: means and SDs of >400 cells. (C) Co-localizations in ATRXF/F MEFs detected by IF-FISH for PML (IF, green) and telomeres (FISH, red), and IF for PML (green) and centromeres (anti-centromere antibody, ACA, red). DNA was stained with DAPI. (D) Quantification of the fraction of telomeres co-localizing with ACA, as assayed in (C). Each data point represents the fraction of telomeres co-localizing with ACA in one cell. Bars: means and SDs of >300 cells. (E-F) Quantification of the percentage of cells with ≥10 PML-ACA (E) or PML-TelC (F) co-localizations, as assayed in (C). Bars: means and SDs of 3 experiments of >100 cells each. (G) Quantification of chromosome ends with reciprocal or single telomere exchanges in ATRXF/F MEFs detected by CO-FISH, from Fig 1G. Pairwise comparisons in panel G were derived from a two-tailed, unpaired t test. All other p-values were derived from a one-way ANOVA with Tukey correction. Symbols as in Fig 1. The underlying numerical data and statistical analysis for each figure panel can be found in S1 Data. ATRX, alpha thalassemia/mental retardation syndrome X-linked chromatin remodeler; ATRXF/F, female embryo with two floxed ATRX alleles; BrdU, bromodeoxyuridine; Cre, recombinase acting on Lox sites; FACS, fluorescence-activated cell sorting; FISH, fluorescence in situ hybridization; FokI-TRF1, FokI nuclease domain and telomeric repeat binding factor 1 fusion protein; IF, immunofluorescence; MEF, mouse embryonic fibroblast; PML, promyelocytic leukemia; SD, standard deviation; TelC, C-rich telomere probe [CCCTAA]3 (TIF) [file pbio.3000594.s003.tif]

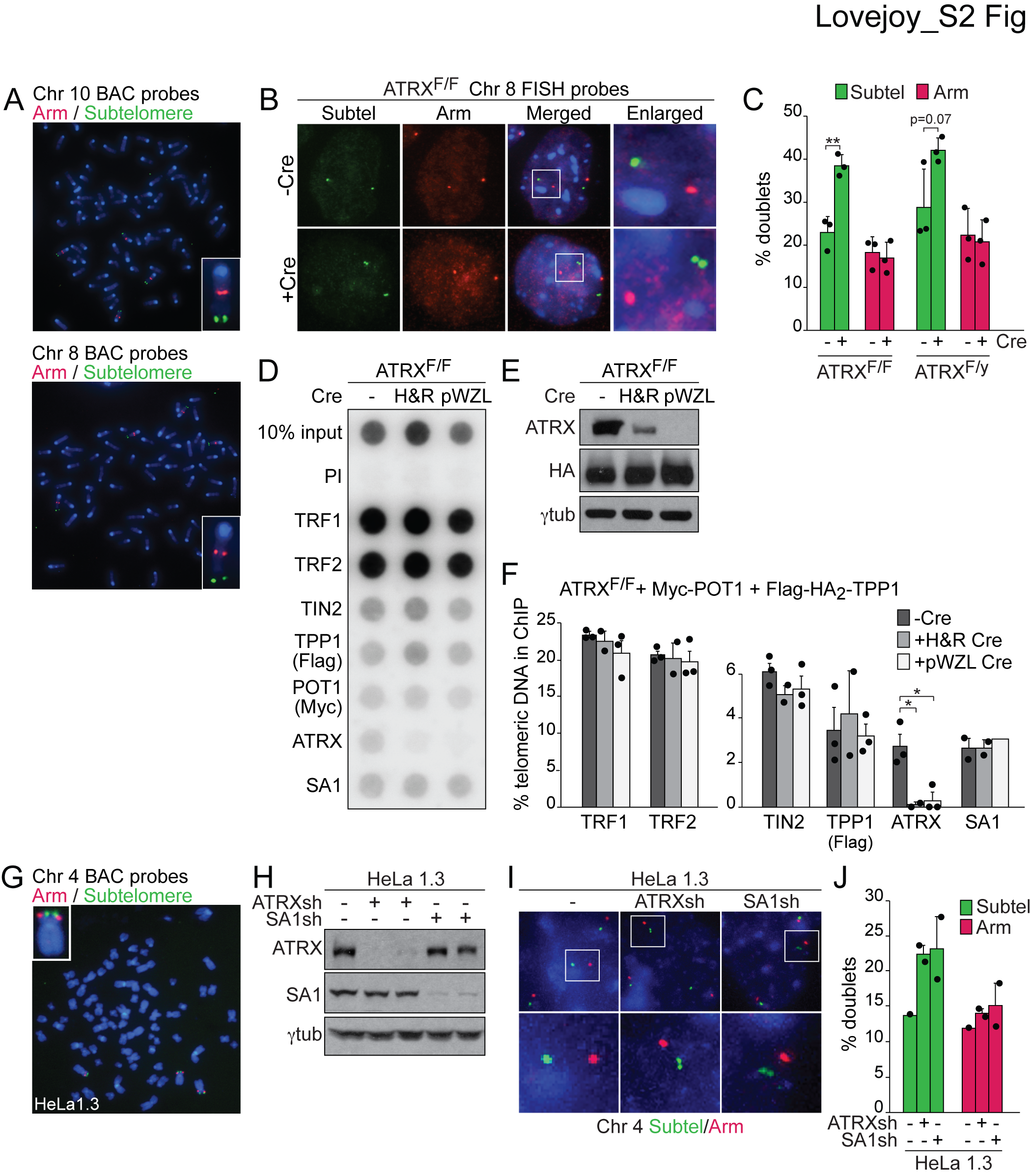

Supplement: S2 Fig — (A) FISH of the arm (red) and subtelomeric (green) probes for mouse Chromosomes 10 and 8 on metaphase spreads from ATRX conditional KO MEFs. (B) FISH of the arm (red) and subtelomeric (green) probes on Chromosome 8 in interphase cells from MEFs described in Fig 2A. (C) Quantification of the Chromosome 8 FISH signals observed as doublets. Bars: means and SDs of 3 experiments. (D) Telomeric ChIP in ATRXF/F MEFs expressing Flag-HA2-TPP1 (anti-Flag) and Myc-POT1 (anti-Myc). PI, pre-immune serum. (E) Immunoblots for ATRX and TPP1 (HA) 4 d after Hit&Run Cre or approximately 40 PDs after pWZL-Cre. γtubulin serves as a loading control. (F) Quantification of the telomeric ChIP signals from 2–3 independent experiments, as assayed in (D). Bars: means and SEMs. The background (PI) was subtracted and ChIP signals were normalized to the input. (G) FISH of the arm (red) and subtelomeric (green) probes on human Chromosome 4 in a metaphase spread from HeLa 1.3 cells. (H) Immunoblot for ATRX and SA1 after shRNA depletion in HeLa 1.3. γtubulin serves as a loading control. (I) FISH of the arm (red) and subtelomeric (green) probes on Chromosome 4 in interphase cells described in (H). (J) Quantification of the Chromosome 4 FISH signals observed as doublets. Bars: means and SEMs from 2 independent shRNAs for both ATRX and SA1. Pairwise comparisons in panel (C) were derived from a two-tailed, unpaired t test. All other p values were derived from a one-way ANOVA with Tukey correction. Symbols as in Fig 1. The underlying numerical data and statistical analysis for each figure panel can be found in S1 Data. ATRX, alpha thalassemia/mental retardation syndrome X-linked chromatin remodeler; ATRXF/F, female embryo with two floxed ATRX alleles; ChIP, chromatin immunoprecipitation; Cre, recombinase acting on Lox sites; FISH, fluorescence in situ hybridization; Flag-HA2-TPP1, epitope-tagged ACD shelterin complex subunit and telomerase recruitment factor; KO, knockout; MEF, mouse embryonic fibroblast [file pbio.3000594.s004.tif]

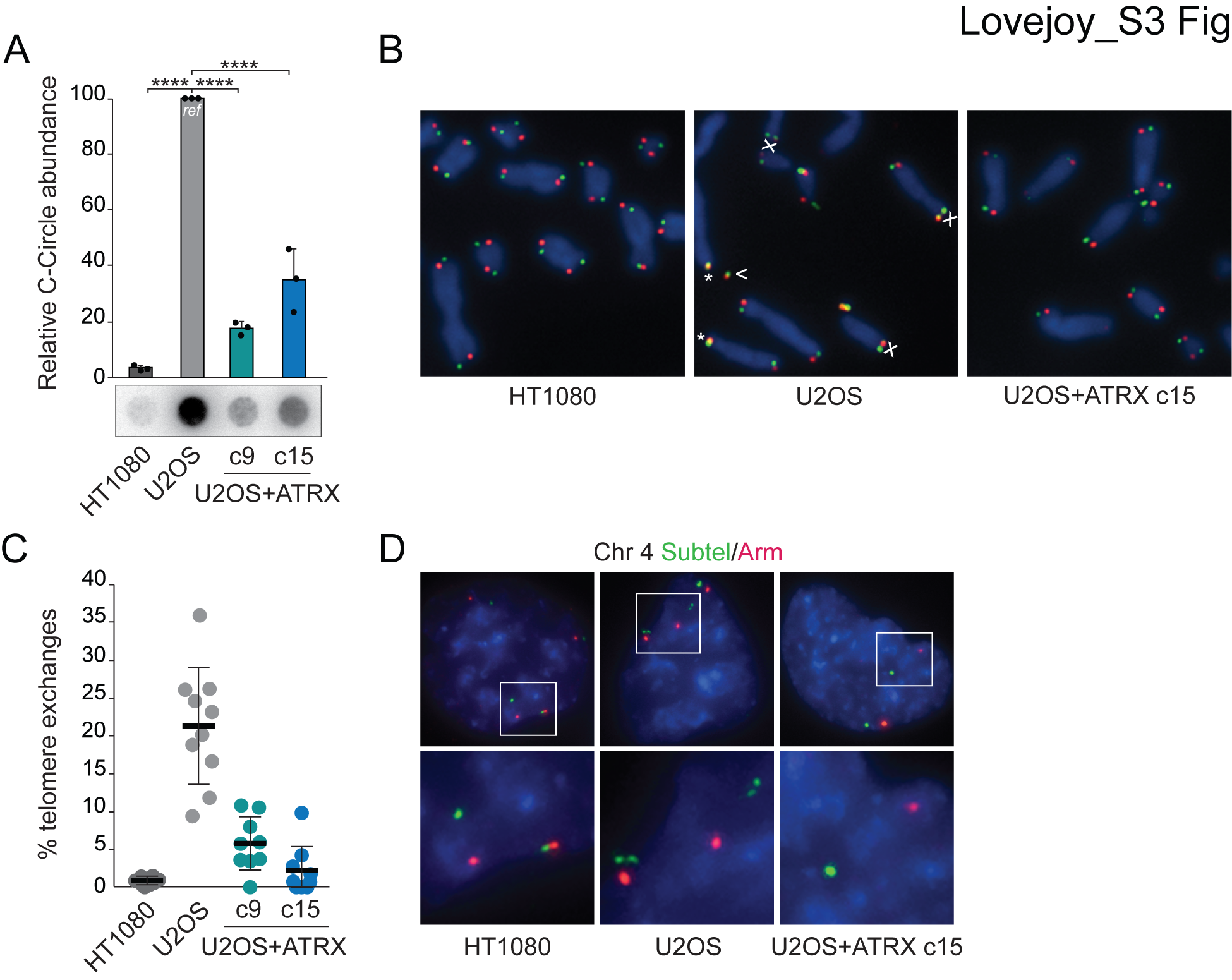

Supplement: S3 Fig — (A) Representative dot blot detecting C-circles with an end-labeled 32P-[CCCTAA]4 probe, and quantification of C-circle abundance in cells described in Fig 2I. Values are presented relative to U2OS (set at 100). Bars: means and SDs of 3 experiments. All p values were derived from a one-way ANOVA with Tukey correction. Symbols as in Fig 1. (B) CO-FISH staining on metaphase spreads from the indicated cell lines, as in Fig 1I. Chromosome ends displaying telomere exchanges are indicated with an x, ECTSs are marked by an arrow, and sister associations are denoted by an asterisk. (C) Quantification of telomere exchanges detected by CO-FISH. Each data point represents the percentage of chromosome ends with telomere exchanges in one metaphase spread. Bars: means and SDs. (D) FISH staining of cell lines described in Fig 2I with probes targeting the arm (red) and subtelomeric (green) regions of Chromosome 4. The underlying numerical data and statistical analysis for each figure panel can be found in S1 Data. ALT, alternative lengthening of telomeres; ATRX, alpha thalassemia/mental retardation syndrome X-linked chromatin remodeler; C-circle, extrachromosomal, circular telomeric DNA with an intact C-rich strand; CO-FISH, chromosome orientation fluorescence in situ hybridization; ECTS, extrachromosomal telomeric signal; FISH, fluorescence in situ hybridization; SD, standard deviation; U2OS, human osteosarcoma cell line. (TIF) [file pbio.3000594.s005.tif]

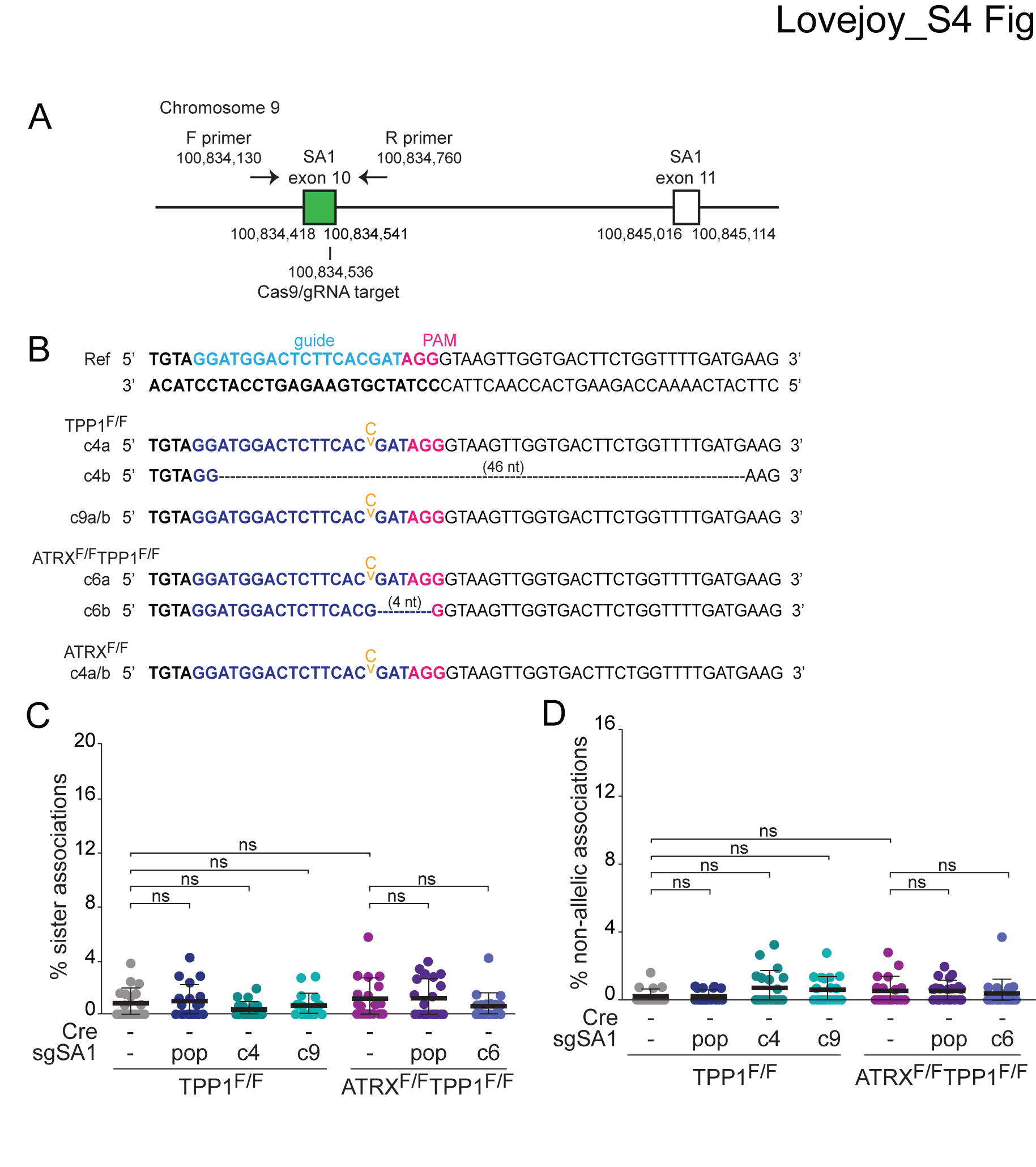

Supplement: S4 Fig — (A) Schematic of the mouse SA1 locus, identifying features relevant to CRISPR/Cas9-mediated gene editing. (B) DNA sequences of the edited SA1 alleles in CRISPR/Cas9-derived KO clones obtained by TOPO (Thermo Fisher Scientific) cloning of PCR products using the primers shown in (A). Edits associated with each allele are specified. Bold text denotes the exon 10 sequence and regular text identifies the intron sequence. (C-D) Quantification of sister (C) and nonallelic (D) telomere associations in control and SA1 KO cells (no Cre) detected by CO-FISH (as in Fig 3). Data points represent the percentage of long arm chromosome ends displaying sister associations and the percentage of all chromatids associated with nonallelic telomeres in one metaphase spread. Bars: means and SDs of 19–20 metaphases from 2 experiments. All p-values were derived from a one-way ANOVA with Tukey correction. Symbols as in Fig 1. The underlying numerical data and statistical analysis for each figure panel can be found in S1 Data. Cas9, CRISPR associated protein 9; CO-FISH, chromosome orientation fluorescence in situ hybridization; Cre, recombinase acting on Lox sites; CRISPR, clustered regularly interspaced short palindromic repeats; KO, knockout; MEF, mouse embryonic fibroblast; ns, not significant; PCR, polymerase chain reaction; SA1, stromal antigen 1; SD, standard deviation. (TIF) [file pbio.3000594.s006.tif]

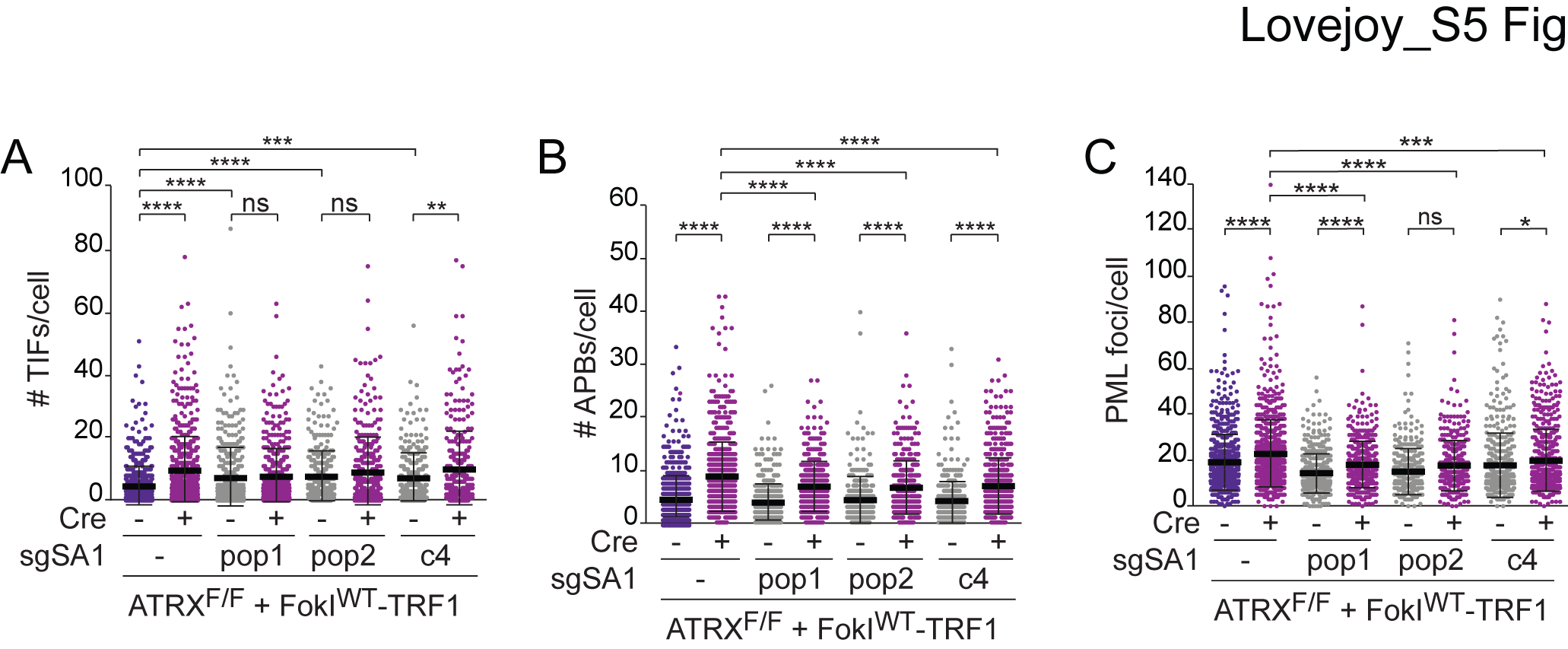

Supplement: S5 Fig — (A-C) Quantification of the number of TIFs per cell (A), telomere-PML co-localizations (APBs) per cell (B), or PML foci per cell (C) in MEFs expressing FokIWT-TRF1, as assayed in Fig 4. Bars: means and SDs of >300 cells. All p-values were derived from a one-way ANOVA with Tukey correction. Symbols as in Fig 1. The underlying numerical data and statistical analysis for each figure panel can be found in S1 Data. ALT, alternative lengthening of telomeres; APB, ALT-associated PML body; FokIWT-TRF1, wild-type FokI nuclease domain and telomeric repeat binding factor 1 fusion protein; KO, knockout; ns, not significant; MEF, mouse embryonic fibroblast; PML, promyelocytic leukemia; SA1, stromal antigen; SD, standard deviation; TIF, telomere dysfunction–induced foci. (TIF) [file pbio.3000594.s007.tif]

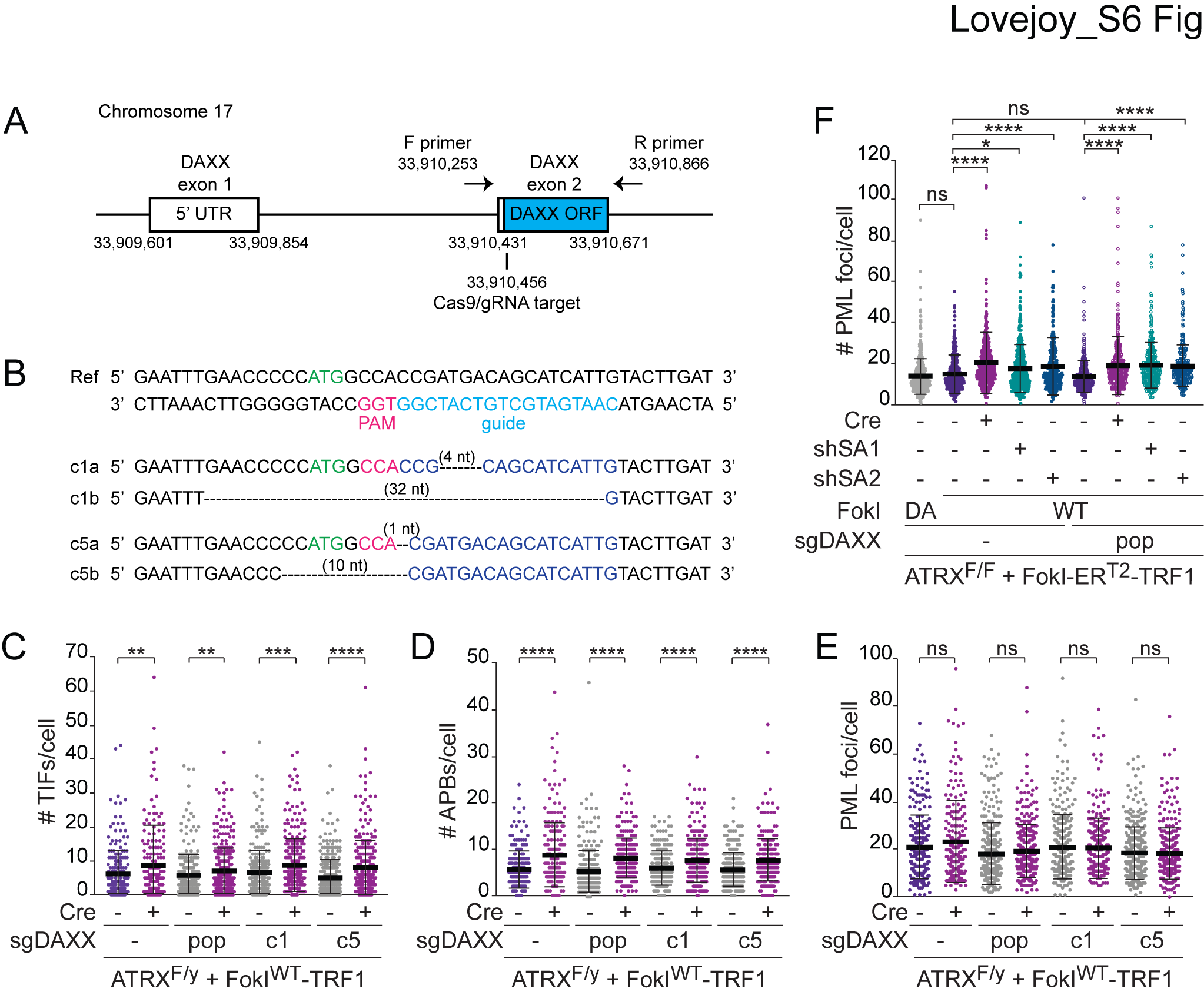

Supplement: S6 Fig — (A) Schematic of the mouse DAXX locus, identifying features relevant to CRISPR/Cas9-mediated gene editing. (B) DNA sequences of the edited DAXX alleles in 2 CRISPR/Cas9-derived ATRXF/y KO clones obtained by TOPO (Thermo Fisher Scientific) cloning of PCR products using the primers shown in (A). Deletions associated with each allele are specified. (C-E) Quantification of the number of TIFs per cell (C), telomere-PML co-localizations (APBs) per cell (D), or PML foci per cell (E) in FokIWT-TRF1–expressing cells, as assayed in Fig 5. Bars: means and SDs of approximately 300 cells for DAXX KO MEFs and approximately 200 cells for control MEFs. (F) Quantification of the number of PML foci per cell in control and a DAXX-targeted population of cells expressing FokI-ERT2-TRF1, after Cre-mediated deletion of ATRX or shRNA depletion of SA1/SA2 (as assayed in Fig 6D). Bars: means and SDs of >330 cells. Pairwise comparisons in panels C, D, and E were derived from a two-tailed, unpaired t test. All other p-values were derived from a one-way ANOVA with Tukey correction. Symbols as in Fig 1. The underlying numerical data and statistical analysis for each figure panel can be found in S1 Data. APB, ALT-associated PML body; ATRX, alpha thalassemia/mental retardation syndrome X-linked chromatin remodeler; ATRXF/y, male embryo with a single floxed allele; Cas9, CRISPR associated protein 9; Cre, recombinase acting on Lox sites; CRISPR, clustered regularly interspaced short palindromic repeats; DAXX, death domain-associated protein; FokI-ERT2-TRF1, tamoxifen-inducible FokI-TRF1 fusion protein; FokIWT-TRF1, wild-type FokI nuclease domain and telomeric repeat binding factor 1 fusion protein; KO, knockout; MEF, mouse embryonic fibroblast; ns, not significant; PCR, polymerase chain reaction; PML, promyelocytic leukemia; SA1, stromal antigen 1; SA2, stromal antigen 2; SD, standard deviation; shRNA, short hairpin RNA; TIF, telomere dysfunction–induced foci. (TIF) [file pbio.3000594.s008.tif]

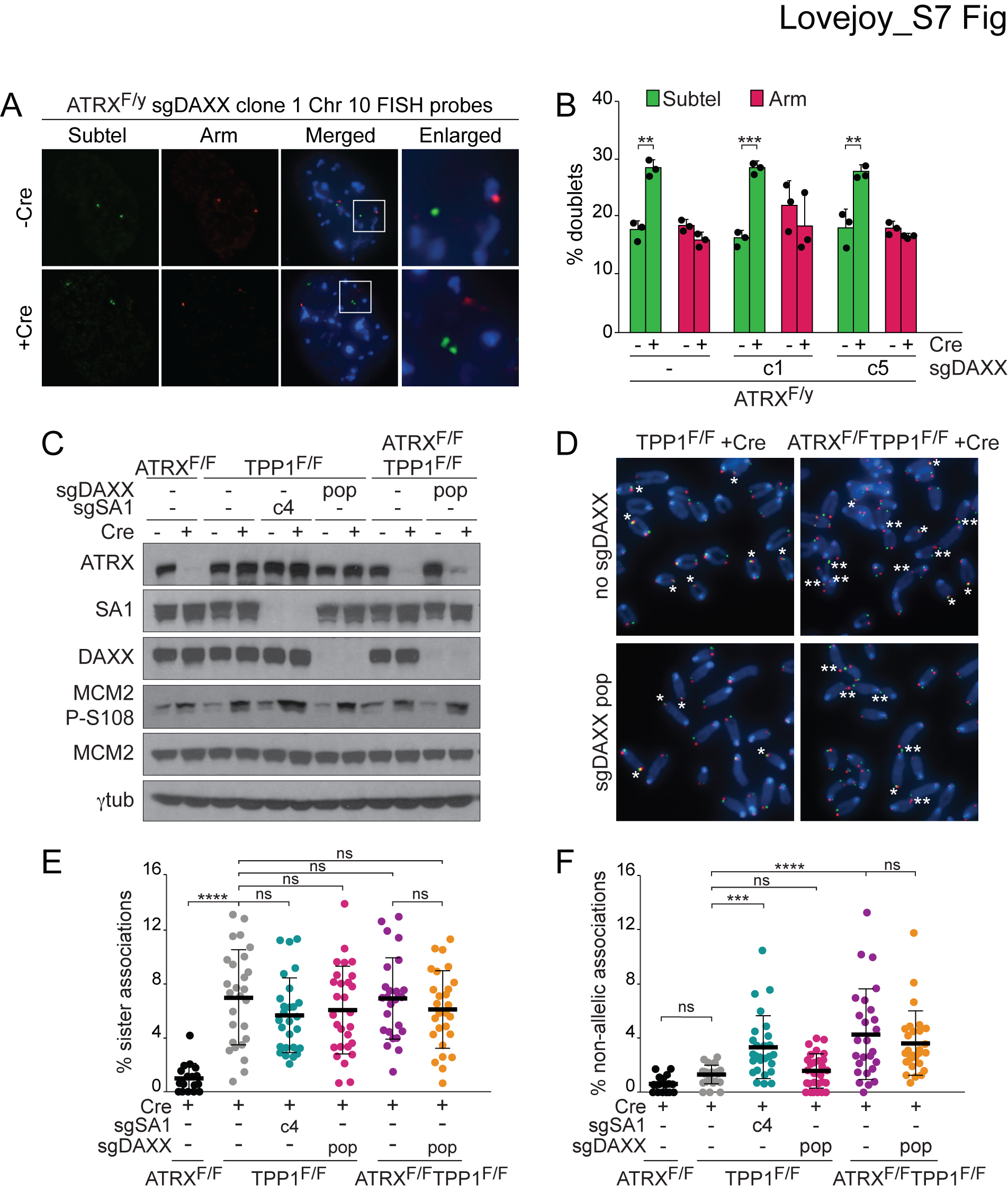

Supplement: S7 Fig — (A) FISH of the arm (red) and subtelomeric (green) probes on Chromosome 10 in interphase cells from DAXX KO MEFs described in Fig 5 and S6 Fig. (B) Quantification of the Chromosome 10 FISH signals observed as doublets. Bars: means and SDs of 3 experiments. (C) Immunoblots showing phosphorylation of MCM2 S108 in TPP1-deficient cells, representative of DNA damage signaling and efficient deletion of TPP1. Cre-mediated deletion of ATRX and CRISPR/Cas9-targeting of SA1 and DAXX are also shown. γtubulin serves as a loading control. (D) Representative images of sister and nonallelic telomere associations detected by CO-FISH staining (as in Fig 1G) on metaphase spreads. Telomere associations are marked by asterisks (*, sister association; **, nonallelic association). (E-F) Quantification of sister (E) and nonallelic (F) telomere associations detected by CO-FISH as in (D). Data points represent the percentage of long arm chromosome ends displaying sister associations and the percentage of all chromatids associated with nonallelic telomeres in one metaphase spread. Bars: means and SDs from 3 experiments. Pairwise comparisons in panel B were derived from a two-tailed, unpaired t test. All other p-values were derived from a one-way ANOVA with Tukey correction. Symbols as in Fig 1. The underlying numerical data and statistical analysis for each figure panel can be found in S1 Data. ATRX, alpha thalassemia/mental retardation syndrome X-linked chromatin remodeler; Cas9, CRISPR associated protein 9; CO-FISH, chromosome orientation fluorescence in situ hybridization; Cre, recombinase acting on Lox sites; CRISPR, clustered regularly interspaced short palindromic repeats; DAXX, death domain-associated protein; FISH, fluorescence in situ hybridization; KO, knockout; MCM2, minichromosome maintenance complex component 2; MEF, mouse embryonic fibroblast; ns, not significant; SA1, stromal antigen 1; SD, standard deviation; TPP1, ACD shelterin complex subunit and telomerase recruitment fact [file pbio.3000594.s009.tif]
